# Passerini Reactions on Biocatalytically Derived Chiral Azetidines

**DOI:** 10.3390/molecules21091153

**Published:** 2016-08-30

**Authors:** Lisa Moni, Luca Banfi, Andrea Basso, Andrea Bozzano, Martina Spallarossa, Ludger Wessjohann, Renata Riva

**Affiliations:** 1 Department of Chemistry and Industrial Chemistry, University of Genova, via Dodecaneso, 31-16146 Genova, Italy; lisa.moni@unige.it (L.M.); banfi@chimica.unige.it (L.B.); andrea.basso@unige.it (A.B.); 3342868@studenti.unige.it (A.B.); martina.spallarossa@gmail.com (M.S.); 2 Leibniz-Institut für Pflanzenbiochemie, Weinberg 3, 06120 Halle (Saale), Germany; wessjohann@ipb-halle.de

**Keywords:** multicomponent reactions, isocyanides, biocatalysis, azetidines

## Abstract

The purpose of this study was to explore a series of Passerini reactions on a biocatalytically derived enantiopure azetidine-2-carboxyaldehyde in order to obtain, in a diastereoselective manner, polyfunctionalised derivatives having the potential to be cyclized to chiral bridged bicyclic nitrogen heterocycles. While diastereoselectivity was poor under classical Passerini conditions, a significant increase of diastereoselectivity (up to 76:24) was gained by the use of zinc bromide as promoter. The methodology has a broad scope and yields are always good.

## 1. Introduction

Isocyanide-based multicomponent reactions [1,2] represent a very powerful tool in diversity-oriented synthesis, allowing the introduction of several diversity inputs in a single step. However, the use of these reactions for the stereoselective obtainment of non-aromatic chiral heterocycles endowed with several stereogenic centres remains challenging. In principle, this task may be reached by using chiral substrates and by combining the multicomponent step with a subsequent cyclization process. However, the diastereochemical control of the new stereogenic centre by the pre-existing ones is in most cases very poor, unless an intramolecular variant is exploited. So far only chiral amines (for the Ugi reaction) [3,4,5,6,7,8] and very specific carboxylic acids (for the Passerini reaction) [9] have brought about significant levels of diastereoselection. On the contrary, little or no success has been obtained with chiral carbonyl compounds. Moreover, racemization/epimerization is usually observed in the Ugi reaction with aldehydes possessing an α-stereogenic carbon [10,11], probably because of imine-enamine equilibria. From this point of view, the Passerini reaction seems more useful, since it is highly stereoconservative, even with stereochemical labile α-chiral aldehydes [12].

**Scheme 1:**
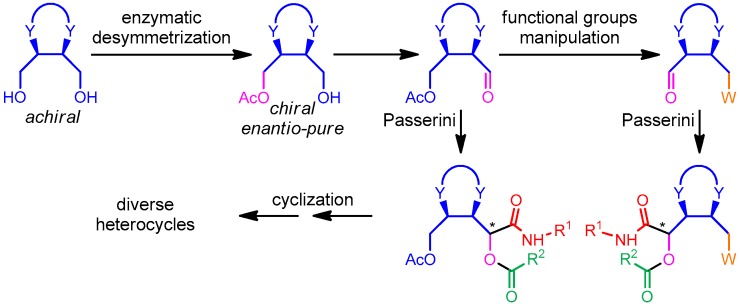
General strategy of combining biocatalysis with the Passerini reaction. “*” represents the new stereogenic centre.

We have devised a general strategy, depicted in Scheme 1, to access chiral enantio-pure heterocycles in a diversity-oriented manner [13]. We mean to exploit, in Passerini reactions, a series of α-chiral aldehydes derived from enzymatic desymmetrization of *meso* cyclic diols. This strategy offers various benefits: (1) the chiral aldehydes are accessible in a very convenient way and high e.e. using low-cost and “green” catalysts; (2) both enantiomers are accessible, using the complementary acylation and hydrolysis processes; (3) apart from the different rings, diversity can also be achieved by introducing, through functional group manipulations, a variety of alternative appendages, indicated as W in Scheme 1 and (4) the additional arm (a masked CH_2_OH group or a CH_2_W group) can be exploited for post-MCR cyclizations leading to a variety of non-aromatic heterocyclic scaffolds (exploration of scaffold diversity). These cyclizations may involve not only the CH_2_OAc or CH_2_W, but also the secondary amide NH and other possible additional moieties installed into the carboxylic or isonitrile components. We have recently published a first “proof of concepts” of this strategy, using erythritol derived building blocks [14].

**Scheme 2:**
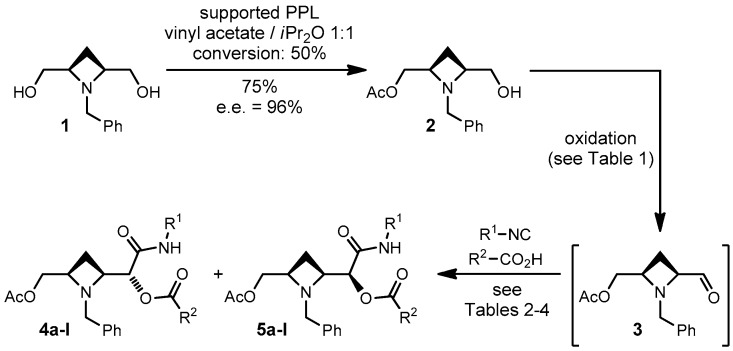
Diastereoselective Passerini reactions on aldehyde **3**.

In this paper we report our preliminary results involving a different *meso* diol, that is 2,4-azetidine-dimethanol **1** (Scheme 2).

## 2. Results

### 2.1. Optimization of Oxidation-Passerini on a Model Compound

*Meso* diol **1** was prepared in four steps from glutaric acid as previously described by us [15], and efficiently desymmetrized with pig pancreatic lipase supported on Celite [15]. The resulting monoacetate **2** was converted into aldehyde **3**, which was not isolated but immediately subjected to a Passerini reaction in CH_2_Cl_2_. For the first optimization studies we used *t*-butyl isocyanide and acetic acid as the other two components.

Oxidation of **2** was found out to be troublesome, probably because of the presence of a tertiary amine. In previous works [10,14] we have found that the method employing catalytic TEMPO (2,2,6,6-tetramethylpiperidin-1-yl)oxyl radical) and stoichiometric BAIB (*bis*-acetoxyiodosobenzene) was particularly useful for generating aldehydes to be used in “one-pot” oxidative Passerini reactions [16]. The only drawback is that the acid component is necessarily acetic acid, generated during the oxidation. However, in this case, this system proved to be unfit (Table 1). The yields of Passerini reaction were unsatisfactory and substantial amounts of unoxidized alcohol **2** were recovered. By increasing the length of the oxidation step (entries 1–3) we could not avoid the residual presence of **2** while the overall yield dropped. Thus we explored other oxidation methods. Also IBX (2-iodoxybenzoic acid), another reagent often used for one-pot oxidative Passerini reactions [16,17,18,19,20], in our hands performed only poorly (entries 4–5) again failing to quantitatively convert **2**.

Eventually we found out that the Swern oxidation was in this case the best one (entries 6–8). After extractive work-up, the resulting crude aldehyde **3** is pure enough to be used as such without any purification, and no epimerization was detected, even using Et_3_N as the base. Furthermore, this oxidation method allows to use any carboxylic acid as input in the Passerini reaction and, contrary to one-pot methodologies, consents to change solvent and other reaction conditions of the Passerini reaction.

Unfortunately, although the overall yield was good, diastereoselectivity was only poor. In all instances the **4a**:**5a** ratio was only 57:43 and this ratio was not improved by lowering the temperature.

Thus we undertook a deep study of alternative reaction conditions in order to improve the d.r. of the reaction. In particular we thought that a Lewis acid could be able to impose a more rigid transition state through its chelation by the basic nitrogen and the carbonyl oxygen. Table 2 shows a screening of Lewis acids. In some cases (entries 2, 4, 5, 11) the diastereomeric ratio was increased, but at the expense of reaction cleanness. Interestingly, with SnCl_4_ (entry 5) a reversal of diastereoselectivity was obtained. On the other hand, zinc (II) derivatives afforded full conversions and clean crude products, although with moderate diastereoselectivity (entries 12–15). However, comparison of entries 12 and 13 made us think that prompt solubility of the Lewis acid may be an important issue. The only difference between these two experiments is that in entry 13 we used a preformed solution of Lewis acid.

Thus we selected zinc bromide (more soluble than ZnCl_2_) and performed a solvent screening (Table 3). As expected, the d.r. was significantly higher in solvent where fast dissolution of ZnBr_2_ takes place, like CH_3_CN or THF. Finally, a decrease of the operating temperature brought about a slight increase in yield and d.r. We selected conditions of entry 8 as the best ones.

### 2.2. Scope of the Reaction

The scope of the reaction was then examined (Table 4), using different isocyanides and carboxylic acids. For comparison, about half of the products were also prepared using the standard Passerini conditions (reaction in CH_2_Cl_2_ at room temperature without any Lewis acid). As can be seen, yields are always excellent under both conditions, although slightly better without the Lewis acid.

On the other hand, the diastereomeric ratios are persistently improved by the addition of ZnBr_2_, reaching values up to 76:24. These ratios may be considered good, if one takes into account the typical poor diastereoselectivity of Passerini reactions and the low steric requirements of linear isocyanides. We performed also two experiments with chiral, enantiomerically pure acids (the two enantiomers of *N*-Fmoc valine) in order to see if diastereoselectivity could be controlled by the chiral acid as well. The results (entries 10, 11) indicate a very little effect of the absolute configuration of the acid on the d.r. and confirms once again the incapacity of chiral carboxylic acids, with very few exceptions [9], to direct the stereochemical course of isocyanide-based multicomponent reactions. In nearly all cases the two diastereomers **4** and **5** could be separated by chromatography (Δ*R*_f_ > 0.05), with the exception of **4**–**5h**. Thus, despite the not exceptional diastereoselectivity, this allows the obtainment of these multifunctionalised adducts possessing 3 stereogenic centres in high e.e. and diastereomeric purity.

### 2.3. Establishment of Relative Configuration

**Scheme 3:**
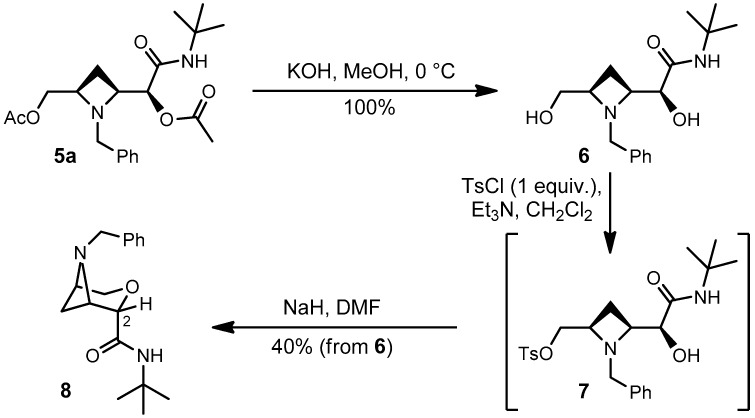
Cyclization of Passerini adduct **5a** to bridged bicyclic system **8**.

It is our aim to exploit adducts **4a**–**l** and **5a**–**l** for a variety of cyclization reactions affording interesting bridged bicyclic heterocycles, but this is outside the scope of the present paper. However, we report here a very preliminary (unoptimized) cyclization, which has allowed to demonstrate the relative configuration of major and minor adducts **4a** and **5a**. Compound **5a** has been first hydrolysed in quantitative yield to diol **6** (Scheme 3). The latter was reacted with *p*-toluenesulphonyl chloride leading to selective tosylation. ^1^H-NMR of the crude showed that tosylation had involved only the primary alcohol. Treatment of the crude tosylate **7** with NaH gave, in moderate yield, compound **8** as a first example of strained bicyclic system. The relative configuration of **8** was demonstrated by nOe experiments. In particular, a 5.9%nOe between the benzylic CH_2_ and H-2 is particularly diagnostic, being not possible for the other diastereomer. From the relative configuration of **8** we have deduced the configuration of **5a**, in the reasonable assumption that the configuration was retained.

Finally, similarities at tlc and ^1^H-NMR indicate that the major product is always **4a**–**l**. Actually, apart from **4**–**5h** (no separation), a comparison between the thin layer chromatographies revealed the *R*_f_ of **5** to be always higher than the one of **4**. Furthermore, in the proton spectra, δ of C*H*OAc is always downfield for **5** with respect to **4** (0.45 > Δδ > 0.18), whereas δ of both diastereotopic C*H*_2_OAc is always downfield for **4** compared to **5**.

## 3. Discussion

**Scheme 4:**
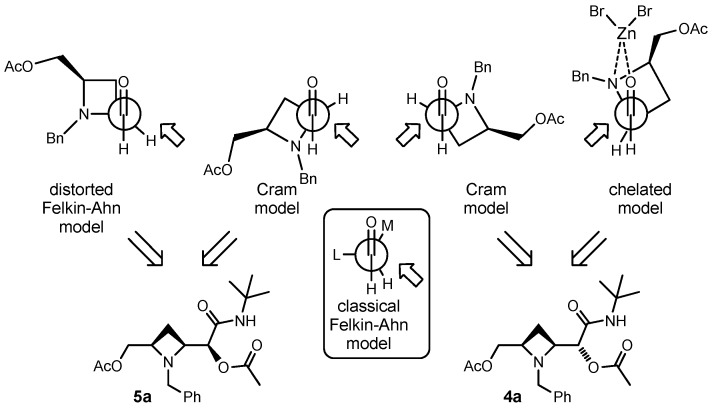
Models for rationalisation of results.

Having established the relative configuration, we may try to rationalise the results. In the absence of Lewis acid, taking into account the typical asymmetric induction achieved with α-aminoaldehydes, one would have expected a diastereoselection dictated by the Felkin-Ahn model, where the nitrogen plays the role of “large” group (Scheme 4). Applying this model, **5**, and not **4**, would have been the major adduct. However, while in acyclic situations the angles among the three bonds in the background are 109.5° (that become 120° in Newman projection), the presence of the azetidine ring alters the situation, since the bond angles inside the ring are expected to be around 90°. Thus, if the C–N bond is orthogonal to the C=O bond, as predicted by Felkin-Ahn model for stereoelectronic reasons, the C-C bond of azetidine should be eclipsed with the C=O, leading to unfavourable steric interactions. In this case, the two Cram models depicted in Scheme 4 seem more reasonable. The one on the right should prevail, since the steric bias around the carbonyl oxygen is less for a trivalent nitrogen than for a tetravalent carbon. Note that in the commonly accepted mechanism of Passerini reaction, protonation of the aldehyde oxygen by the carboxylic acid takes place concurrently with isocyanide addition. Anyway, the competition between the two Cram conformations and the Felkin-Ahn one can explain, together with the low bulkiness of incoming isocyanide, the low diastereoselection achieved.

In the presence of zinc bromide, coordination of the metal by the nitrogen and by the carbonyl oxygen should result in a chelated model, which correctly predicts **4** as the major product, and this may explain the increase in diastereoselectivity.

Although the final diastereomeric ratios achieved are still only moderate, we have demonstrated, once again, the possibility, at least with chiral aldehydes bearing heteroatoms, to increase the diastereoselectivity using Lewis acids as catalysts or mediators. The high yields, the possibility, in nearly all cases, to separate the two diastereomers, and the high potential of adducts **4**–**5** for undergoing a variety of cyclization reactions leading to new, bridged, chiral heterocycles (such as **8**), make the here presented methodology quite valuable for the synthesis of libraries to be applied in medicinal chemistry. Researches directed towards the obtainment of such heterocycles in a diversity-oriented way are in progress and will be reported in due course. It should be noted that the tertiary amine may be either cleaved to a secondary amine by debenzylation or alkylated to a quaternary cation. Therefore, we think that the bridged rigid systems that can be obtained through cyclization protocols may be useful also as chiral organocatalysts in the form of secondary amines (imminium-enamine catalysis), tertiary amines (base catalysis) or quaternary salts (phase-transfer catalysis).

## 4. Materials and Methods

### 4.1. General Remarks

Column chromatographies were done with the “flash” methodology using 220–400 mesh silica. Petroleum ether (40–60 °C) is abbreviated as PE. All reactions using dry solvents were carried out under a nitrogen (or argon if specified) atmosphere. Diol **1** and alcohol **2** were prepared as previously described [15]. Characterization of all new compounds is reported in the Supplementary Material.

### 4.2. Synthesis of ((2R,4S)-1-Benzyl-4-formylazetidin-2-yl)methyl Acetate (***3***)

A solution of DMSO (212 μL, 2.99 mmol) in DCM (1.42 mL) was added over 15 min to a stirred solution of oxalyl chloride (133 μL, 1.57 mmol) in DCM (2.90 mL) at −78 °C. Upon completion of the addition, the mixture was stirred at −78 °C for 15 min, followed by addition of a solution of alcohol (2*R*,4*S*) **2** (314 mg, 1.26 mmol) in DCM (0.8 mL) over 10 min at −78 °C and the resulting mixture was stirred for 15 min. Then Et_3_N (350 μL, 2.52 mmol) was added dropwise over 10 min. The resulting mixture was allowed to warm to 0 °C and stirred at 0 °C for 30 min. The reaction was quenched by addition of water (10 mL). The layers were separated and the aqueous phase was extracted with DCM (2 × 10 mL). The combined organic layers were washed with aqueous NaHCO_3_ (5%) (20 mL) extracted 3 times (10 mL DCM each) and finally with brine, dried over sodium sulphate and concentrated to afford 322 mg of a yellow orange oil. The crude was directly employed in the next Passerini reactions.

### 4.3. General Procedure for Passerini Reaction under Classical Conditions

A solution of crude aldehyde **3** in dry CH_2_Cl_2_ (0.5 M) was treated with the carboxylic acid (1.2 eq) at r.t. After 2 min the isocyanide (1.2 eq) was added. The resulting solution was stirred at r.t. for 1 h, and then quenched with aqueous NaHCO3 (5%). Extraction with CH_2_Cl_2_ (3 × 10 mL), drying with Na_2_SO_4_, and concentration gave a crude product that was purified by flash column chromatography on silica gel with PE/Et_2_O or *n*-hexane/Et_2_O mixtures.

### 4.4. General Procedure for Passerini Reaction with ZnBr_2_

A solution of ZnBr_2_ (1.0 eq) in dry THF (0.2 M) was placed at −20 °C under nitrogen atmosphere and the isocyanide (1.1 eq) was added. After stirring for 10 min, a solution of the crude aldehyde (1.0 eq) and carboxylic acid (1.1 eq) in THF (0.2 M) was added dropwise. The resulting solution (0.4 M) was stirred at −20 °C for 18 h. The reaction was quenched with aqueous NaHCO_3_ (5%) (10 mL) and the organic phase was extracted with ethyl acetate (2 × 10 mL), dried over sodium sulphate, and concentrated. The residue was purified by flash column chromatography on silica gel with PE/Et_2_O or *n*-hexane/Et_2_O mixtures.

### 4.5 Synthesis of (2S)-2-((2S,4R)-1-Benzyl-4-(hydroxymethyl)azetidin-2-yl)-N-(tert-butyl)-2-hydroxyacetamide (***6***)

A solution of **5a** (170 mg, 0.44 mmol) in MeOH (870 μL) was treated, at 0 °C with a solution of KOH (73 mg, 1.31 mmol) in MeOH (1.3 mL). After stirring for 1 h, the reaction was quenched with aqueous NH_4_Cl (5%) (4 mL) and concentrated under vacuum, in order to remove methanol. The mixture was taken up with saturated aqueous NH_4_Cl and extracted with AcOEt (10 mL × 3). The organic phase was washed with brine, dried over Na_2_SO_4_, and concentrated under vacuum to afford 137 mg of yellow-white, analytically pure, crystals (quant. yield).

### 4.6 Synthesis of (1S,2S,5R)-6-Benzyl-N-(tert-butyl)-3-oxa-6-azabicyclo[3.1.1]heptane-2-carboxamide (***8***)

A solution of diol **6** (50 mg, 0.16 mmol) in CH_2_Cl_2_ (5 mL) was cooled to 0 °C and treated with Et_3_N (82 μL, 0.60 mmol), *N*,*N*-dimethylaminopyridine (DMAP) (2 mg, 16 µmol) and 4-toluenesulphonyl chloride (31 mg, 0.16 mmol). The mixture was allowed to reach room temperature for 24 h, then it was treated with saturated aqueous NaHCO_3_ (5%, 20 mL) and extracted with CH_2_Cl_2_ (50 + 25 mL). The combined organic phases were washed with brine (20 mL), dried (Na_2_SO_4_), and concentrated to give the crude tosylate **7** as a yellow oil. *R*_f_ = 0.48 (PE/Et_2_O 1:3). The crude product could be used in the next step without further purification. To a solution of crude **7** in dry DMF (8 mL) at r.t., sodium hydride (8 mg, 60% of a dispersion in paraffine, 0.20 mmol) was added. The mixture was stirred for 1 h at r.t., then treated with saturated aqueous NH_4_Cl (40 mL) and extracted with Et_2_O (50 mL × 2). The combined organic phases were washed with brine (10 mL), dried (Na_2_SO_4_), and concentrated. The crude product was eluted from a column of silica gel with PE/Et_2_O 1:3 to give **8** (19 mg, 40%) as an amorphous solid.

## Figures and Tables

**Table 1:** Optimization of oxidation conditions ^1^.

Entry	Oxidant	Temp.	Solvent	Time ^2^	Work-Up ^3^	Yield ^4^	Note
1	TEMPO-BAIB	r.t.	CH_2_Cl_2_	1 h	none	58%	residual 2
2	TEMPO-BAIB	r.t.	CH_2_Cl_2_	3 h	none	37%	residual 2
3	TEMPO-BAIB	r.t.	CH_2_Cl_2_	16 h	none	<15%	residual 2
4	IBX	r.t.	THF	24 h	none	23%	residual 2
5	IBX	r.t.	THF	96 h	none	23%	residual 2
6	Swern (*i*Pr_2_NEt)	−78 °C	CH_2_Cl_2_	8 h	extraction	75%	
7	Swern (Et_3_N)	−78 °C	CH_2_Cl_2_	80 min	extraction	82%	
8	Swern (Et_3_N)	−78 °C	CH_2_Cl_2_	30 min	extraction	84%	

^1^ After oxidation, crude aldehyde **3** was immediately reacted, for 1 h at r.t., with *t*-butyl isocyanide (1.2 equiv.) and acetic acid (1.2 equiv.) in the same solvent used for oxidation. D.r. was always 57:43; ^2^ Oxidation time; ^3^ Work-up after oxidation; ^4^ Isolated yield of **4a** + **5a** after chromatography.

**Table 2:** Effect of various Lewis acids on diastereoselectivity ^1^.

Entry	Lewis Acid ^2^	Conversion ^3^	Notes	4a:5a Ratio ^3^
1	none	100%		57:43
2	FeCl_3_	100%	partially unclean	63:37
3	Ti(OiPr)_3_Cl	98%	very unclean	59:41
4	TiCl_4_	100%	very unclean	66:34
5	SnCl_4_	100%	partially unclean	37:63
6	SnCl_2_	100%		57:43
7	AlCl_3_	89%		60:40
8	MgBr_2_	100%		57:43
9	Cu(OTf)_2_	<5%		-
10	CuBr_2_	80%	very unclean	58:42
11	BF_3_·Et_2_O	80%	partially unclean	70:30
12	ZnCl_2_	100%		58:42
13	ZnCl_2_^4^	100%		63:37
14	ZnBr_2_	100%		59:41
15	ZnI_2_	100%		59:41

^1^ Crude aldehyde **3**, obtained through Swern oxidation, was immediately reacted, for 1 h at room temperature, with *t*-butyl isocyanide (1.2 equiv.) and acetic acid (1.2 equiv.) in CH_2_Cl_2_; ^2^ 1 equiv. of Lewis acid was used; ^3^ Conversion and d.r., were determined by ^1^H-NMR of the crude; ^4^ A preformed 1 M solution of ZnCl_2_ was used.

**Table 3:** Optimization of ZnBr_2_ mediated Passerini ^1^.

Entry	Solvent	Temp.	Time	Yield ^2^	4a:5a Ratio ^3^
1	*i*Pr_2_O	25 °C	1 h	76%	56:44
2	Et_2_O	25 °C	1 h	56%	59:41
3	Bu_2_O	25 °C	1 h	59%	54:46
4	*t*BuMeO	25 °C	1 h	43%	65:35
5	MeCN	25 °C	1 h	76%	67:33
6	THF	25 °C	1 h	83%	68:32
7	THF	−20 °C	2 h	87%	70:30
8	THF	−20 °C	18 h	86%	70:30
9	THF	−40 °C	18 h	50%	69:31

^1^ Crude aldehyde **3**, obtained through Swern oxidation, was mixed with acetic acid (1.1 equiv.), and added to a solution of *t*-butyl isocyanide (1.1 equiv.) and ZnBr_2_ (1.1 equiv.). Then it was allowed to react for the indicated time; ^2^ Determined by ^1^H-NMR of the crude, using benzyl benzoate as internal standard; ^3^ Determined by ^1^H-NMR of the crude.

**Table 4:** Scope of Passerini reaction of aldehyde **3**.

Entry	Products	R ^1^	R ^2^	With ZnBr_2_^1^		Classic Conditions ^2^	
				Yield ^3^	4:5 Ratio ^4^	Yield ^3^	4:5 Ratio ^4^
1	**4**,**5a**	*t*Bu	CH_3_	86%	70:30	84%	57:43
2	**4**,**5b**	*t*Bu	*t*Bu	75%	70:30	84%	57:43
3	**4**,**5c**	*cy*Hex	*t*Bu	76%	68:32	81%	57:43
4	**4**,**5d**	*t*Bu	*n*Bu	73%	76:24	83%	56:44
5	**4**,**5e**	*cy*Hex	*n*Bu	77%	75:25	85%	58:42
6	**4**,**5f**	*t*Bu	Ph	72%	69:31	81%	56:44
7	**4**,**5g**	*cy*Hex	Ph	72%	72:28	72%	57:43
8	**4**,**5h**	CH_2_CO_2_Et	3-BrC_6_H_4_	71%	66:34	- ^5^	-
9	**4**,**5i**	2,6-diMeC_6_H_3_	5-Cl-2-thienyl	67%	69:31	- ^5^	-
10	**4**,**5j**	*n*Bu	(*S*)FmocVal	73%	70:30	- ^5^	-
11	**4**,**5k**	*n*Bu	(*R*)FmocVal	69%	73:27	- ^5^	-
12	**4**,**5l**	Bn	CH_2_OMe	64%	70:30	- ^5^	-

^1^ For conditions see entry 8 of Table 3; ^2^ Reactions performed in CH_2_Cl_2_ at 25 °C for 1 h with no additive; ^3^ Isolated yields; ^4^ Determined by ^1^H-NMR of the crude; ^5^ Reaction not performed.

## Supplementary Materials

The following are available online at: http://www.mdpi.com/1420-3049/21/9/1153/s1. Full characterization of new compounds and copies of ^1^H- and ^13^C-NMR spectra.
