# Supplementary material for: Passerini Reactions on Biocatalytically Derived Chiral Azetidines
Source: Molecules. 2016 Aug 30;21(9):1153. doi: 10.3390/molecules21091153 (PMC6273022; doi:10.3390/molecules21091153)
Supplement: Supplementary file 1 [file molecules-21-01153-s001.pdf]

Supplementary Materials: Passerini Reactions on Biocatalytically Derived Chiral Azetidines

Lisa Moni, Luca Banfi, Andrea Basso, Andrea Bozzano, Martina Spallarossa, Ludger Wessjohann and Renata Riva

Table of Contents

Contents

General remarks S1
Characterization of New Compounds S1
Copies of ^{1}H- and ^{13}C-NMR spectra of compounds S11

Experimental Procedures and Spectroscopic Characterization of New Compounds

1. General Remarks

NMR spectra were taken in $CDCl_3$ at 300 MHz or 400 MHz (1H), and 75 MHz or 100 MHz (^{13}C), using, as internal standard, TMS (^{1}H-NMR in $CDCl_3$; 0.000 ppm) or the central peak of $CDCl_3$ (^{13}C in $CDCl_3$; 77.02 ppm). Chemical shifts are reported in ppm (δ scale), coupling constants are reported in Hertz. Peak assignments were also made with the aid of gCOSY and gHSQC experiments. In an ABX system, the proton A is considered upfield and the B proton is considered downfield. IR spectra were recorded directly on solid, oil, or foamy samples, with the ATR (attenuated total reflectance) technique. TLC analyses were carried out on silica gel plates, viewed at UV (λ = 254 nm) and developed with Hanessian stain (dipping into a solution of $(NH_4)_4MoO_4\cdot 4H_2O$ (21 g) and $Ce(SO_4)_2\cdot 4H_2O$ (1 g) in H_2SO_4 (31 mL) and H_2O (469 mL) and warming), or with Iodine stain (leaving the TLC in a chamber containing solid iodine and leaving it for a few minutes until spots appear) or Dragendorff stain (dipping into a solution derived from mixing solution A of bismuth oxynitrate (1.7 g), acetic acid (40 mL) and water (80 mL) with solution B of potassium iodide (36 g) and water (100 mL); solution A:solution B mix is 1:1). R_f values were measured after an elution of 7–9 cm. HRMS were performed employing an ESI+ ionization method with a Thermo Fisher Scientific Orbitrap Elite (ion trap mode—high resolution) Velos Pro (ionisation) Ultimate 3000 (UPLC System for injection).

2. Characterization of All New Compounds

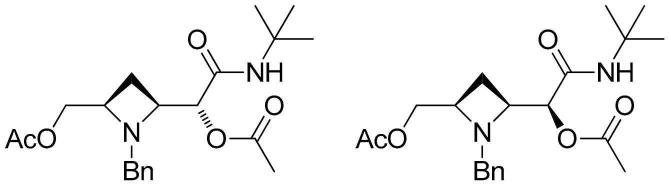

4a **5a**

(1R) and (1S) 1-((2S,4R)-4-(Acetoxymethyl)-1-benzylazetidin-2-yl)-2-(tert-butylamino)-2-oxoethyl acetates **4a** *and* **5a**

4a: R_f = 0.51, *n*Hex/Et_2O 1:4 developed with Dragendorff stain; $[\alpha]_D$ = −69.3 (*c* = 0.45, $CHCl_3$); ^{1}H-NMR (300 MHz, $CDCl_3$): δ 7.36–7.24 (m, 5H; H arom), 6.74 (broad s, 1H; NH), 4.79 (d, *J* = 4.3 Hz, 1H; C*H*OAc), 3.92 and 3.89 (AB part of ABX syst., J_{AB} = 11.2, J_{AX} = 5.4, J_{BX} = 6.5 Hz, 2H; CH_2OAc), 3.77 and 3.66 (AB syst., J_{AB} = 13.2 Hz, 2H; CH_2 of Bn), 3.57 (td, *J* = 8.1, 4.6 Hz, 1H; H-2), 3.36 (tt, *J* = 8.1, 6.3 Hz, 1H; H-4), 2.21 and 1.74 (AB part of ABX syst., J_{AB} = 10.4, J_{AX} = 7.9, J_{BX} = 7.9 Hz, 2H; CH_2-3), 2.10 (s, 3H; OAc), 1.92 (s, 3H; OAc), 1.35 (s, 9H; *t*Bu). ^{13}C-NMR (75 MHz, $CDCl_3$): δ 170.7 (C=O), 169.8 (C=O),

166.3(C=O), 137.6 (Cq), 129.1(2 CH arom), 128.5 (2 CH arom), 127.6 (CH arom), 73.5 (CHOAc), 67.4 (CH_2OAc), 61.7 (CH-2), 61.6 (CH_2 of Bn), 60.2 (CH-4), 51.4 (Cq), 28.7 (CH_3 of *t*Bu), 23.3 (CH_2-3), 20.8 (OAc), 20.7 (OAc). HRMS (ESI) calcd for $C_{21}H_{31}N_2O_5$ $[M + H]^+$ 391.2227; found 39.2230.

5a: R_f = 0.59, *n*Hex/Et_2O 1:4 developed with Dragendorff stain; $[\alpha]_D$ = −77.1 (*c* = 0.5, $CHCl_3$); ^{1}H-NMR (300 MHz, $CDCl_3$) δ 7.32–7.20 (m, 5H; H arom), 5.98 (broad s, 1H; NH), 5.11 (d, *J* = 3.6 Hz, 1H; C*H*OAc), 3.81 and 3.50 (AB syst., J_{AB} = 12.9 Hz, 2H, CH_2 of Bn), 3.74 and 3.66 (AB part of ABX syst., J_{AB} = 11.3, J_{AX} = 5.7, J_{BX} = 5.7 Hz, 2H; C*H*$_2$OAc), 3.64 (dt, *J* = 8.1, 3.6 Hz, 1H; H-2), 3.27 (tt, *J* = 8.1, 6.0 Hz, 1H; H-4), 2.18 (s, 3H, OAc), 2.13–2.07 (m, 1H; H-3a), 1.88–1.79 (m, 1H; H-3b), 1.86 (s, 3H; OAc), 1.35 (s, 9H; *t*Bu). ^{13}C-NMR (75 MHz, $CDCl_3$) δ 170.8 (C=O), 169.3 (C=O), 166.7(C=O), 138.1 (Cq), 129.1(2 CH arom), 128.2 (2 CH arom), 127.3 (CH arom), 73.7 (CHOAc), 67.7 (CH_2OAc), 62.1 (CH-2), 60.9 (CH_2 of Bn), 59.7 (CH-4), 51.4 (Cq), 28.7 (CH_3 of *t*Bu), 21.6 (CH_2-3), 21.2 (OAc), 20.7 (OAc). IR: υ (cm^{-1}) 2967, 2874, 2803, 2159, 2025, 1969, 1738, 1673, 1523, 1480, 1454, 1366, 1216, 1088, 1032, 975, 916, 750, 699, 666. HRMS (ESI) calcd for $C_{21}H_{31}N_2O_5$ $[M + H]^+$ 391.2227; found 39.2229.

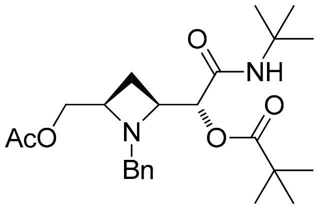

4b

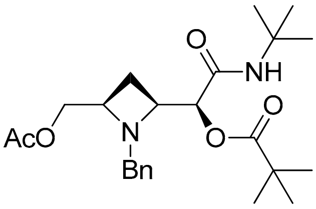

5b

(1R) and (1S) 1-((2S,4R)-4-(Acetoxymethyl)-1-benzylazetidin-2-yl)-2-(tert-butylamino)-2-oxoethyl 2,2-dimethylpropanoates **4b** *and* **5b**

4b: R_f = 0.28, *n*Hex/Et_2O 5:4 developed with I_2; $[\alpha]_D$ = −3.85 (*c* = 1.9, $CHCl_3$); ^{1}H-NMR (400 MHz, $CDCl_3$) δ 7.40–7.19 (m, 5H; H arom), 6.40 (broad s, 1H; NH), 4.89 (d, *J* = 4.0 Hz, 1H; C*H*O(C=O)), 3.90–3.77 (m, 3H; CH_2OAc and 1 H of CH_2Ph), 3.71 (td, *J* = 8.2, 4.1 Hz, 1H; H-2), 3.59 (d, *J* = 13.6 Hz, 1H; 1 H of CH_2Ph), 3.32 (tt, *J* = 7.6, 6.4 Hz, 1H; H-4), 2.19 (dt, *J* = 11.0, 7.9 Hz, 1H; H-3a), 1.84 (s, 3H; OAc), 1.74 (dt, *J* = 11.0, 8.4 Hz, 1H; H-3b), 1.30 (s, 18H; 2 *t*Bu); ^{13}C-NMR (100 MHz, $CDCl_3$) δ 177.1 (C=O), 170.7 (C=O), 166.7 (C=O), 137.7 (Cq), 129.0 (2 CH arom), 128.4 (2 CH arom), 127.4 (CH arom), 73.9 (CHO(C=O)), 67.6 (CH_2OAc), 61.31 (H-2), 61.29 (CH_2 of Bn), 60.2 (H-4), 51.3 (Cq), 38.9 (Cq), 28.6 (*t*Bu), 27.2 (*t*Bu), 23.0 (CH_2-3), 20.6 (OAc). IR: υ (cm^{-1}) 2967, 2934, 2873, 2161, 2025, 1737, 1683, 1515, 1495, 1480, 1455, 1394, 1365, 1227, 1138, 1034, 896, 752, 699, 666. HRMS (ESI) calcd for $C_{24}H_{37}N_2O_5$ $[M + H]^+$ 433.2697; found 433.2692.

5b: R_f = 0.37, *n*Hex/Et_2O 5:4 developed with I_2; $[\alpha]_D$ = −39.7 (*c* = 1.6, $CHCl_3$); ^{1}H-NMR (400 MHz, $CDCl_3$) δ 7.35–7.16 (m, 5H; H arom), 5.98 (broad s, 1H; NH), 5.10 (d, *J* = 3.5 Hz, 1H; C*H*O(C=O)), 3.87 and 3.48 (AB syst., J_{AB} = 13.0 Hz, 2H; CH_2 of Bn), 3.76 and 3.64 (AB part of ABX syst., J_{AB} = 11.2, J_{AX} = 5.9, J_{BX} = 5.8 Hz, 2H; C*H*$_2$OAc), 3.67 (dt, *J* = 8.2, 3.5 Hz, 1H; H-2), 3.25 (tt, *J* = 7.7, 6.1 Hz, 1H; H-4), 2.10 (dt, *J* = 10.5, 7.7 Hz, 1H; H-3a), 1.89–1.80 (m, 1H; H-3b), 1.84 (s, 3H; OAc), 1.34 (s, 9H; *t*Bu), 1.31 (s, 9H; *t*Bu); ^{13}C-NMR (100 MHz, $CDCl_3$) δ 176.5 (C=O), 170.9 (C=O), 167.1 (C=O), 138.3 (Cq), 129.3 (2 CH arom), 128.4 (2 CH arom), 127.4 (CH arom), 73.0 (CHO(C=O)), 67.8 (CH_2OAc), 62.4 (CH-2), 61.1 (CH_2 of Bn), 60.4 (CH-4), 51.4 (Cq), 39.0 (Cq), 28.8 (*t*Bu), 27.5 (*t*Bu), 22.3 (CH_2-3), 20.8 (OAc). IR: υ (cm^{-1}) 2967, 2934, 2873, 2161, 2025, 1737, 1683, 1515, 1495, 1480, 1455, 1394, 1365, 1227, 1138, 1034, 896, 752, 699, 666. HRMS (ESI) calcd for $C_{24}H_{37}N_2O_5$ $[M + H]^+$ 433.2697; found 433.2696.

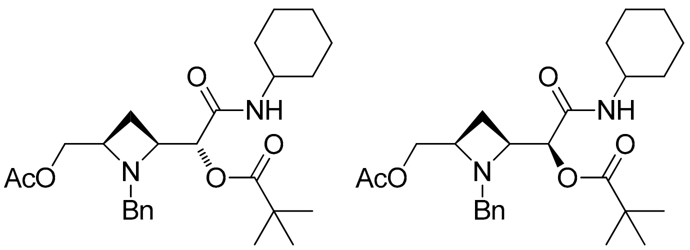

4c **5c**

(1R) and (1S) 1-((2S,4R)-4-(Acetoxymethyl)-1-benzylazetidin-2-yl)-2-(cyclohexylamino)-2-oxoethyl 2,2-dimethylpropanoates **4c** *and* **5c**

4c: R_f = 0.35, *n*Hex/Et_2O 1:4 developed with Dragendorff stain; $[\alpha]_D$ = −9.6 (*c* = 2.2, $CHCl_3$); ^{1}H-NMR (400 MHz, $CDCl_3$) δ 7.39–7.18 (m, 5H; H arom of Bn), 6.48 (broad d, *J* = 7.6 Hz, 1H; NH), 4.94 (d, *J* = 4.1 Hz, 1H; CHO(C=O)), 3.91–3.78 (m, 2H; CH_2OAc), 3.82 and 3.60 (AB syst., J_{AB} = 13.6 Hz, 2H; CH_2 of Bn), 3.78–3.66 (m, 2H; H-2 and CH of Cy), 3.33 (centre of m, 1H; H-4), 2.18 (dt, *J* = 10.6, 7.9 Hz, 1H; H-3a), 1.96–1.55 (m, 5H; H-3b and 4H of Cy), 1.87 (s, 3H; OAc), 1.43–0.99 (m, 6H; Cy), 1.29 (s, 9H; *t*Bu); ^{13}C-NMR (100 MHz, $CDCl_3$) δ 177.1 (C=O), 170.6 (C=O), 166.7 (C=O), 137.6 (Cq), 129.1 (2 CH arom), 128.4 (2 CH arom), 127.4 (CH arom), 73.6 (CHO(C=O)), 67.4 (CH_2OAc), 61.2 (CH-2), 60.0 (CH-4), 48.0 (CH of Cy), 38.9 (CH_2 of Bn), 33.1 (CH_2 of Cy), 32.8 (CH_2 of Cy), 27.3 (Cq), 27.2 (*t*Bu), 25.4 (CH_2 of Cy), 24.7 (CH_2 of Cy), 24.7 (CH_2 of Cy), 23.1 (CH_2-3), 20.6 (OAc). HRMS (ESI) calcd for $C_{26}H_{39}N_2O_5$ $[M + H]^+$ 459.2853; found 459.2851.

5c: R_f = 0.42, *n*Hex/Et_2O 1:4 developed with Dragendorff stain; $[\alpha]_D$ = −50.1 (*c* = 1.5, $CHCl_3$); ^{1}H-NMR (400 MHz, $CDCl_3$) δ 7.33–7.19 (m, 5H; H arom of Bn), 6.04 (broad d, *J* = 7.7 Hz, 1H; NH), 5.20 (d, *J* = 3.6 Hz, 1H; C*H*O(C=O)), 3.89 and 3.49 (AB syst., J_{AB} = 13.0 Hz, 2H; CH_2 of Bn), 3.84–3.73 (m, 1H; CH of Cy), 3.76 and 3.63 (AB part of ABX syst., J_{AB} = 11.3, J_{AX} = 6.0, J_{BX} = 5.9 Hz, 2H, CH_2OAc), 3.69 (td, *J* = 8.2, 3.6 Hz, 1H; H-2), 3.26 (tt, *J* = 7.6, 6.1 Hz, 1H; H-4), 2.10 (dt, *J* = 10.5, 7.7 Hz, 1H; H-3a), 1.96–1.80 (m, 3H; H-3b and 2H of Cy), 1.84 (s, 3H; OAc), 1.74–1.53 (m, 3H; Cy), 1.44–1.05 (m, 5H; Cy), 1.32 (s, 9H; *t*Bu); ^{13}C-NMR (100 MHz, $CDCl_3$) δ 176.4 (C=O), 170.7 (C=O), 166.8 (C=O), 137.9 (Cq), 129.2 (2 CH arom), 128.2 (2 CH arom), 127.3 (CH arom), 72.6 (CHO(C=O)), 67.5 (CH_2OAc), 62.2 (CH-2), 60.8 (CH_2 of Bn), 60.3 (CH-4), 47.7 (CH of Cy), 38.9 (Cq), 33.1 (CH_2 of Cy), 32.8 (CH_2 of Cy), 27.3 (*t*Bu), 25.4 (CH_2 of Cy), 24.6 (CH_2 of Cy), 22.1 (CH_2 of Cy), 20.6 (OAc). IR: υ (cm^{-1}) 3437, 3329, 2932, 2855, 2364, 2160, 2024, 1971, 1732, 1666, 1519, 1479, 1452, 1396, 1366, 1232, 1135, 1032, 892, 751, 699, 666. HRMS (ESI) calcd for $C_{26}H_{39}N_2O_5$ $[M + H]^+$ 459.2853; found 459.2852.

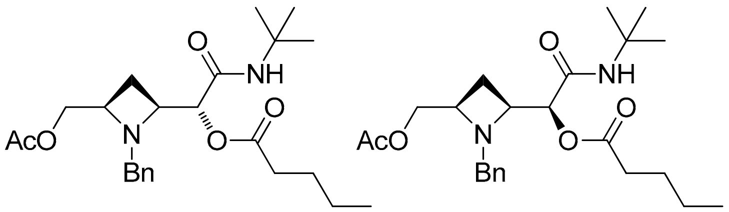

4d **5d**

(1R) and (1S) 1-((2S,4R)-4-(Acetoxymethyl)-1-benzylazetidin-2-yl)-2-(tert-butylamino)-2-oxoethyl pentanoates **4d** *and* **5d**

4d: R_f = 0.31, *n*Hex/Et_2O 1:1 developed with Dragendorff stain; $[\alpha]_D$ = −81.1 (*c* = 0.33, $CHCl_3$); ^{1}H-NMR (400 MHz, $CDCl_3$) δ 7.39–7.28 (m, 5H; H arom), 6.80 (broad s, 1H; NH), 4.95 (d, *J* = 4.2 Hz, 1H; C*H*O(C=O)), 3.99 (d, *J* = 5.8 Hz, 2H; CH_2OAc), 3.91and 3.73 (AB syst., J_{AB} = 13.5 Hz, 2H; CH_2 of Bn), 3.86–3.77 (m, 1H; H-2), 3.54–3.45 (m, 1H; H-4), 2.51–2.31 (m, 2H; CH_2 of *n*Bu), 2.21 (dt, *J* = 11.3, 8.1 Hz, 1H; H-3a), 1.94 (s, 1H; OAc), 1.92–1.81 (m, 1H; H-3b), 1.71–1.59 (m, 2H; CH_2 of *n*Bu), 1.46–1.29 (m, 2H; CH_2 of *n*Bu), 1.35 (s, 9H; *t*Bu), 0.92 (t, *J* = 7.4 Hz, 3H; CH_3 of *n*Bu.^{13}C-NMR (100 MHz, $CDCl_3$) δ 172.6

(C=O), 170.6 (C=O), 166.2 (C=O), 133.4 (Cq), 129.8 (2 CH arom), 128.7 (2 CH arom), 128.1 (CH arom), 73.3 (CHO(C=O)), 66.4 (CH_2OAc), 61.4 (CH), 60.4 (CH_2), 59.7 (CH), 51.6 (Cq), 33.7 (CH_2), 28.7 (*t*Bu), 26.9 (CH_2), 23.1 (CH_2), 22.2 (CH_2), 20.6 (CH_3), 13.7 (CH_3). IR: υ (cm^{-1}) 2962, 2933, 2873, 2159, 2024, 1969, 1739, 1681, 1523, 1454, 1392, 1365, 1226, 1166, 1108, 1094, 1036, 977, 907, 750, 699, 666. HRMS (ESI) calcd for $C_{24}H_{37}N_2O_5$ $[M + H]^+$ 433.2697; found 433.2694.

5d: R_f = 0.37, *n*Hex/Et_2O 1:1 developed with Dragendorff stain; $[\alpha]_D$ = −30.0 (*c* = 0.6, $CHCl_3$); ^{1}H-NMR (400 MHz, $CDCl_3$) δ 7.33–7.23 (m, 5H; CH arom), 6.04 (broad s, 1H; NH), 5.14 (d, *J* = 3.8 Hz, 1H; C*H*O(C=O)), 3.85 and 3.54 (AB syst., J_{AB} = 13.0 Hz, 2H; CH_2 of Bn), 3.78 (dd, *J* = 11.3, 5.7 Hz, 1H; 1 H of C*H*$_2$OAc), 3.74–3.64 (m, 2H; H-2 and 1 H of C*H*$_2$OAc), 3.37–3.26 (m, 1H; H-4), 2.42 (t, *J* = 7.5 Hz, 2H; CH_2 of *n*Bu), 2.13 (dt, *J* = 10.8, 7.9 Hz, 1H; H-3a), 1.93–1.82 (m, 1H; H-3b), 1.87 (s, 3H; OAc), 1.71–1.63 (m, 2H; CH_2 of *n*Bu), 1.46–1.36 (m, 2H; CH_2 of *n*Bu), 1.34 (s, 9H; *t*Bu), 0.95 (t, *J* = 7.3 Hz, 3H; CH_3 of *n*Bu); ^{13}C-NMR (100 MHz, $CDCl_3$) δ 172.0 (C=O), 170.7 (C=O), 166.8 (C=O), 137.6 (Cq), 129.3 (2 CH arom), 128.3 (2 CH arom), 127.4 (CH arom), 73.3 (CHO(C=O)), 67.4 (CH_2OAc), 62.1 (H-2), 60.7 (CH_2 of Bn), 59.9 (H-4), 51.4 (Cq), 34.0 (CH_2 of *n*Bu), 28.7 (*t*Bu), 27.0 (CH_2 of *n*Bu), 22.3 (CH_2 of *n*Bu), 21.9 (CH_2-3), 20.7 (OAc), 13.7 (CH_3 of *n*Bu). HRMS (ESI) calcd for $C_{24}H_{37}N_2O_5$ $[M + H]^+$ 433.2697; found 433.2694.

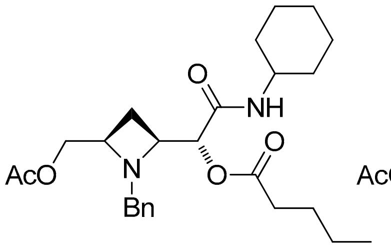

4e

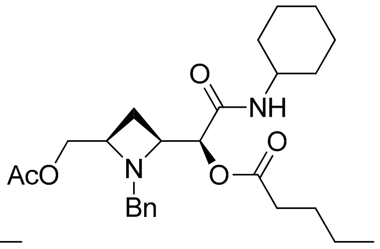

5e

(1R) and (1S) 1-((2S,4R)-4-(Acetoxymethyl)-1-benzylazetidin-2-yl)-2-(cyclohexylamino)-2-oxoethyl pentanoates **4e** *and* **5e**

4e: R_f = 0.31, *n*Hex/Et_2O 1:1 developed with Dragendorff stain; $[\alpha]_D$ = −9.7 (*c* = 1.0, $CHCl_3$); ^{1}H-NMR (400 MHz, $CDCl_3$) δ 7.36–7.23 (m, 5H; H arom of Bn), 6.57 (broad d, *J* = 7.7 Hz, 1H; NH), 4.90 (d, *J* = 4.6 Hz, 1H; CHO(C=O)), 3.91 and 3.86 (AB part of ABX syst., J_{AB} = 11.4, J_{AX} = 5.5, J_{BX} = 6.4 Hz, 2H; CH_2OAc), 3.76 and 3.65 (AB syst., J_{AB} = 13.2 Hz, 2H; CH_2 of Bn), 3.77–3.69 (m, 1H), 3.66–3.59 (m, 1H), 3.34 (tt, *J* = 7.9, 6.2 Hz, 1H; H-4), 2.43–2.24 (m, 2H; CH_2 of *n*Bu), 2.20 (dt, *J* = 11.0, 7.8 Hz, 1H; H-3a), 1.92 (s, 3H; OAc), 1.98–1.81 (m, 2H; CH_2 of Cy), 1.75–1.55 (m, 6H; H-3b, CH_2 of *n*Bu, 3 H of Cy), 1.44–1.25 (m, 4H; CH_2 of *n*Bu and 2 H of Cy), 1.24–1.06 (m, 3H; 3 H of Cy), 0.91 (t, *J* = 7.3 Hz, 3H; CH_3 of *nBu*); ^{13}C-NMR (100 MHz, $CDCl_3$) δ 172.7 (C=O), 170.7 (C=O), 166.5 (C=O), 137.6 (Cq), 129.1 (2 CH arom), 128.4 (2 CH arom), 127.5 (CH arom), 73.8 (CHO(C=O)), 67.4 (CH_2OAc), 61.6 (CH-2), 61.4 (CH_2 of Bn), 60.0 (CH-4), 48.2 (CH of Cy), 33.8 (CH_2), 33.2 (CH_2), 32.9 (CH_2), 27.0 (CH_2), 25.5 (CH_2), 24.8 (CH_2), 23.2 (CH_2), 22.2 (2 CH_2), 20.7 (OAc), 13.7 (CH_3 of *n*Bu). IR: υ (cm^{-1}) 3301, 2931, 2855, 2363, 2159, 2025, 1738, 1657, 1530, 1452, 1367, 1231, 1164, 1093, 1033, 970, 891, 751, 699, 666. HRMS (ESI) calcd for $C_{26}H_{39}N_2O_5$ $[M + H]^+$ 459.2853; found 459.2856.

5e: R_f = 0.37, *n*Hex/Et_2O 1:1 developed with Dragendorff stain; $[\alpha]_D$ = −33.9 (*c* = 0.8, $CHCl_3$); ^{1}H-NMR (400 MHz, $CDCl_3$) δ 7.32–7.18 (m, 5H; H arom of Bn), 6.01 (broad d, *J* = 8.0 Hz, 1H; NH), 5.23 (d, *J* = 3.5 Hz, 1H; CHO(C=O)), 3.83 and 3.50 (AB syst., J_{AB} = 12.9 Hz, 2H; CH_2 of Bn), 3.81–3.70 (m, 2H), 3.65 (m, 2H), 3.33–3.19 (m, 1H; H-4), 2.43 (t, *J* = 7.4 Hz, 2H; CH_2 of *n*Bu), 2.10 (dt, *J* = 10.7, 7.9 Hz, 1H; H-3a), 1.96–1.79 (m, 3H; H-3b, 2 H of Cy), 1.86 (s, 3H; OAc), 1.76–1.56 (m, 5H; CH_2 of *n*Bu, 3 H of Cy), 1.48–1.30 (m, 4H; CH_2 of *n*Bu, 2 H of Cy), 1.25–1.04 (m, 3H; Cy), 0.95 (t, *J* = 7.3 Hz, 3H; CH_3 of *n*Bu); ^{13}C-NMR (100 MHz, $CDCl_3$) δ 172.0 (C=O), 170.8 (C=O), 166.7 (C=O), 138.2 (Cq), 129.2 (2 CH arom), 128.2 (2 CH arom), 127.2 (CH arom), 73.1 (CHO(C=O)), 67.6 (CH_2OAc), 62.2 (CH-2), 60.9 (CH_2 of Bn), 59.8 (CH-4), 47.9 (CH of Cy), 34.0 (CH_2), 33.1 (CH_2), 32.9 (CH_2), 27.0 (CH_2), 25.5 (CH_2), 24.7 (CH_2), 22.3 (2 CH_2), 21.7 (CH_2), 20.6 (OAc), 13.7 (CH_3). HRMS (ESI) calcd for $C_{26}H_{39}N_2O_5$ $[M + H]^+$ 459.2853; found 459.2857.

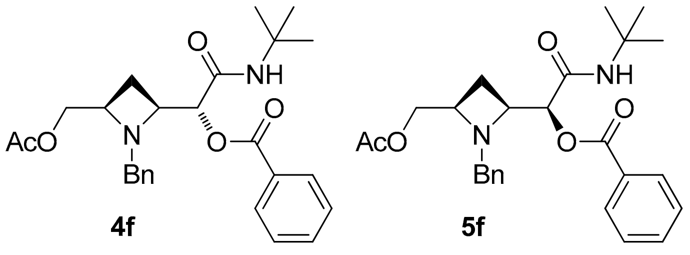

4f **5f**

(1R) and (1S) 1-((2S,4R)-4-(Acetoxymethyl)-1-benzylazetidin-2-yl)-2-(tert-butylamino)-2-oxoethyl benzoates **4f** *and* **5f**

4f: R_f = 0.41, *n*Hex/Et_2O 1:1 developed with Dragendorff stain; $[\alpha]_D$ = −23.3 (*c* = 1.7, $CHCl_3$); ^{1}H-NMR (400 MHz, $CDCl_3$) δ 8.11 (d, *J* = 7.4 Hz, 2H; H arom), 7.59 (t, *J* = 7.4 Hz, 1H; H arom), 7.47 (t, *J* = 7.4 Hz, 2H; H arom), 7.35–7.20 (m, 5H; H arom), 6.7 (broad s, 1H; NH), 5.08 (d, *J* = 4.1 Hz, 1H; CHOBz), 3.89 (d, *J* = 5.9 Hz, 2H; CH_2OAc), 3.84 and 3.68 (AB syst., J_{AB} = 13.2 Hz, 2H; CH_2 of Bn), 3.78 (td, *J* = 8.1, 4.1 Hz, 1H; H-2), 3.38 (tt, *J* = 7.9, 6.0 Hz, 1H; H-4), 2.27 (dt, *J* = 11.1, 7.9 Hz, 1H; H-3a), 1.91–1.80 (m, 3H; H-3b), 1.87 (s, 3H; OAc), 1.34 (s, 9H; *t*Bu); ^{13}C-NMR (100 MHz, $CDCl_3$) δ 170.7 (C=O), 166.4 (C=O), 165.4 (C=O), 137.7 (Cq), 133.4 (CH arom), 129.8 (2 CH arom), 129.4 (Cq), 129.1 (2 CH arom), 128.6 (2 CH arom), 128.5 (2 CH arom), 127.6 (CH arom), 74.1 (CHOBz), 67.4 (CH_2OAc), 61.7 (CH-2), 61.6 (CH_2 of *n*Bu), 60.3 (CH-4), 51.4 (Cq), 28.7 (*t*Bu), 23.2 (CH_2-3), 20.6 (OAc). HRMS (ESI) calcd for $C_{26}H_{33}N_2O_5$ $[M + H]^+$ 453.2384; found 433.2381.

5f: R_f = 0.49, *n*Hex/Et_2O 1:1 developed with Dragendorff stain; $[\alpha]_D$ = −40.9 (*c* = 1.7, $CHCl_3$); ^{1}H-NMR (400 MHz, $CDCl_3$) δ 8.08 (d, *J* = 7.2 Hz, 2H; H arom), 7.63 (t, *J* = 7.4 Hz, 1H; H arom), 7.51 (t, *J* = 7.7 Hz, 2H; H arom), 7.24–7.18 (m, 5H; H arom of Bn), 6.11 (broad s, 1H; NH), 5.35 (d, *J* = 3.6 Hz, 1H; CHOBz), 3.89 and 3.53 (AB syst., J_{AB} = 13.1 Hz, 2H; CH_2 of Bn), 3.79 (td, *J* = 8.1, 3.6 Hz, 1H; H-2), 3.73 and 3.56 (AB part of ABX syst., J_{AB} = 11.3, J_{AX} = 5.7, J_{BX} = 5.9 Hz, 2H, CH_2OAc), 3.28 (tt, *J* = 7.8, 5.8 Hz, 1H; H-4), 2.19 (dt, *J* = 10.6, 7.8 Hz, 1H; H-3a), 2.00 (dt, *J* = 10.6, 8.3 Hz, 1H; H-3b), 1.77 (s, 3H; OAc), 1.36 (s, 9H; *t*Bu); ^{13}C-NMR (75 MHz, $CDCl_3$) δ 170.7 (C=O), 166.8 (C=O), 164.9 (C=O), 138.1 (Cq), 133.5 (CH arom), 129.7 (Cq), 129.7 (2 CH arom), 129.0 (2 CH arom), 128.7 (2 CH arom), 128.2 (2 CH arom), 127.2 (CH arom), 74.1 (CHOBz), 67.5 (CH_2OAc), 62.3 (CH-2), 60.8 (CH_2 of Bn), 60.0 (CH-4), 51.4 (Cq), 28.7 (*t*Bu), 22.0 (CH_2-3), 20.6 (OAc). IR: υ (cm^{-1}) 3436, 3342, 3063, 2967, 2874, 2362, 2159, 2025, 1723, 1674, 1520, 1452, 1393, 1365, 1226, 1176, 1095, 1069, 1026, 976, 907, 753, 709, 666. HRMS (ESI) calcd for $C_{26}H_{33}N_2O_5$ $[M + H]^+$ 453.2384; found 433.2383.

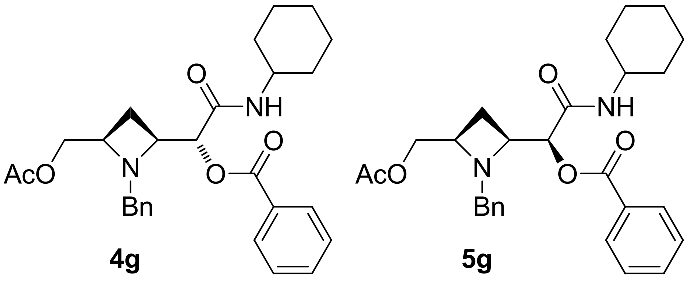

4g **5g**

(1R) and (1S) 1-((2S,4R)-4-(Acetoxymethyl)-1-benzylazetidin-2-yl)-2-(cyclohexylamino)-2-oxoethyl benzoates **4g** *and* **5g**

4g: R_f = 0.32, *n*Hex/Et_2O 1:4 developed with Dragendorff stain; $[\alpha]_D$ = −17.2 (*c* = 0.5, $CHCl_3$); ^{1}H-NMR (400 MHz, $CDCl_3$) δ 8.10 (d, *J* = 7.4 Hz, 2H; H arom of Bz), 7.60 (t, *J* = 7.4 Hz, 1H; H arom of Bz), 7.47 (t, *J* = 7.4 Hz, 2H; H arom of Bz), 7.33–7.18 (m, 5H; H arom of Bn), 6.57 (broad d, *J* = 7.7 Hz, 1H; NH), 5.16 (d, *J* = 4.3 Hz, 1H; CHOBz), 3.82 and 3.67 (AB syst., *J* = 13.3 Hz, 2H; CH_2 of Bn), 3.92–3.71 (m, 4H; CH_2OAc, H-2 and CH of Cy), 3.42–3.32 (m, 1H; H-4), 2.26 (dt, *J* = 11.1, 7.9 Hz, 1H; H-3a), 1.98–1.80 (m, 2H; H of Cy), 1.88 (s, 3H; OAc), 1.74–1.54 (m, 4H; H of Cy), 1.40–1.10 (m, 4H; H of Cy); ^{13}C-NMR

(100 MHz, $CDCl_3$) δ 170.7 (C=O), 166.5 (C=O), 165.5 (C=O), 137.5 (Cq), 133.5 (CH arom), 129.8 (2 CH arom), 129.4 (Cq), 129.2 (2 CH arom), 128.6 (2 CH arom), 128.5 (2 CH arom), 127.5 (CH arom), 74.4 (CHOAc), 67.4 (CH_2OBz), 61.6 (CH), 61.5 (CH_2 of Bn), 60.1 (CH), 48.3 (CH-4), 33.1 (CH_2), 32.9 (CH_2), 29.7 (CH_2), 25.5 (CH_2), 24.8 (CH_2), 23.2 (CH_2), 20.7 (OAc). IR: υ (cm^{-1}) 3305, 2931, 2854, 2362, 1722, 1658, 1602, 1531, 1451, 1366, 1316, 1240, 1176, 1096, 1069, 1027, 966, 891, 844, 803, 750, 708, 666. HRMS (ESI) calcd for $C_{28}H_{35}N_2O_5$ $[M + H]^+$ 479.2540; found 479.2537.

5g: R_f = 0.38, *n*Hex/Et_2O 1:4 developed with Dragendorff stain; $[\alpha]_D = -12.7$ (c = 0.4, $CHCl_3$); ^{1}H-NMR (400 MHz, $CDCl_3$) δ 8.13–8.06 (m, 2H, H arom of Bz), 7.64 (t, J = 7.4 Hz, 1H; H arom of Bz), 7.51 (t, J = 7.7 Hz, 2H; H arom of Bz), 7.21 (broad s, 5H; H arom of Bn), 6.14 (broad d, J = 7.1 Hz, 1H; NH), 5.45 (d, J = 3.3 Hz, 1H; CHOBz), 3.90 and 3.54 (AB syst., J_{AB} = 13.0 Hz, 2H; CH_2 of Bn), 3.86–3.76 (m, 2H), 3.72 (dd, J = 11.3, 5.6 Hz, 1H), 3.57–3.48 (m, 1H); 3.33–3.20 (m, 1H, H-4), 2.18 (dt, J = 10.4, 7.8 Hz, 1H; H-3a), 2.06–1.82 (m, 3H; H-3b and 2 H of Cy), 1.77 (s, 3H; OAc), 1.75–1.51 (m, 2H; H of Cy), 1.47–1.02 (m, 6H; H of Cy); ^{13}C-NMR (100 MHz, $CDCl_3$) δ 170.7 (C=O), 166.6 (C=O), 165.0 (C=O), 138.0 (Cq), 133.6 (CH arom), 129.7 (2 CH arom), 129.7 (Cq), 129.1 (2 CH arom), 128.7 (2 CH arom), 128.2 (2 CH arom), 127.2 (CH arom), 73.1 (CHOBz), 67.4 (CH_2OAc), 62.3 (CH-2), 60.7 (CH_2 of Bn), 60.0 (CH-4), 48.1 (CH of Cy), 33.2 (CH_2), 32.9 (CH_2), 25.5 (CH_2), 24.8 (CH_2), 24.8 (CH_2), 22.0 (CH_2), 20.6 (OAc). HRMS (ESI) calcd for $C_{28}H_{35}N_2O_5$ $[M + H]^+$ 479.2540; found 479.2539.

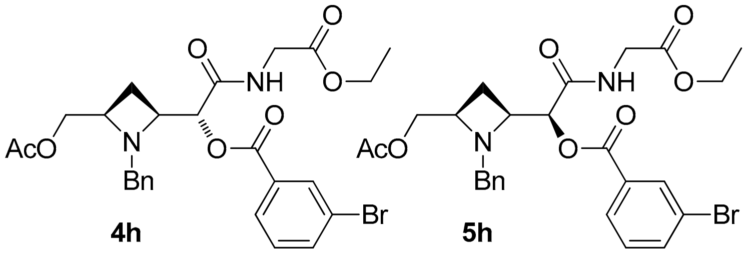

(1R) and (1S) 1-((2S,4R)-4-(Acetoxymethyl)-1-benzylazetidin-2-yl)-2-((2-ethoxy-2-oxoethyl)amino)-2-oxoethyl 3-bromobenzoates **4h** and **5h**: R_f = 0.44, PE/Et_2O 1:4 developed with I_2 (in this case the two isomers were not separated; characterizations have been made on the mixture); ^{1}H-NMR (300 MHz, $CDCl_3$) (Selected data of mixture of isomers **4h**(M) + **5h**(m)) δ 8.25 (t, J = 1.7 Hz, 1H; H arom (m)), 8.16 (t, J = 1.7 Hz, 1H; H arom (M)), 8.07–8.02 (m, 1H; H arom (m)), 8.01–7.95 (m, 1H; H arom (M)), 7.88 (broad t, J = 5.2 Hz, 1H; NH (M)), 7.76 (ddd, J = 8.1, 1.9, 1.1 Hz, 1H; H arom (m)), 7.69 (ddd, J = 8.1, 1.9, 1.1 Hz, 1H; H arom (M)), 7.40 (t, J = 7.9 Hz, 1H; H arom (m)), 6.87 (broad t, J = 5.1 Hz, 1H; NH (m)), 5.47 (d, J = 3.4 Hz, 1H; CHO(C=O) (m)), 5.04 (d, J = 4.4 Hz, 1H; CHO(C=O) (M)), 1.96 (s, 3H; OAc (M)), 1.80 (s, 3H; OAc (m)), 1.28 (t, J = 7.1 Hz, 6H; CH_3 of Et (M + m)); ^{13}C-NMR (75 MHz, $CDCl_3$, mixture of isomers M + m) δ 170.9 (C=O (M)), 170.7 (C=O (m)), 169.5 (C=O (M)), 169.3 (C=O (m)), 167.6 (C=O (m)), 167.4 (C=O (M)), 164.0 (C=O (M)), 163.7 (C=O (m)), 137.7 (Cq (m)), 137.1 (Cq (M)), 136.6 (CH (m)), 136.3 (CH (M)), 132.8 (CH (M)), 132.7 (CH (m)), 131.3 (Cq (m)), 131.1 (Cq (M)), 130.3 (CH (m)), 130.0 (CH (M)), 129.1 (2 CH (M)), 129.0 (2 CH (m)), 128.6 (2 CH (M)), 128.4 (2 CH (m)), 128.24 (CH (m)), 128.22 (CH (M)), 127.7 (CH (M)), 127.3 (CH (m)), 122.8 (Cq (m)), 122.5 (Cq (M)), 73.9 (CHO(C=O) (m)), 73.4 (CHO(C=O) (M)), 67.2 (CH_2OAc (m)), 66.9 (CH_2OAc (M)), 62.1 (CH-2 (m)), 61.8 (CH_2 of Bn (M)), 61.7 (CH_2 of Et (m)), 61.5 (CH_2 of Et (M)), 61.5 (CH-2 (M)), 60.6 (CH_2 of Bn (m)), 60.4 (CH-4 (M)), 59.8 (CH-4 (m)), 41.2 (CH_2NH (M)), 41.0 (CH_2NH (m)), 22.9 (CH_2-3 (M)), 21.8 (CH_2-3 (m)), 20.8 (OAc (M)), 20.6 (OAc (m)), 14.1 (CH_3 of Et (M + m)). IR: υ (cm^{-1}) 3309, 2959, 1731, 1663, 1570, 1537, 1494, 1454, 1438, 1422, 1374, 1334, 1305, 1288, 1254, 1236, 1207, 1169, 1137, 1126, 1083, 1068, 1034, 1000, 974, 913, 898, 887, 863, 841, 807, 770, 747, 728, 702, 672, 637, 615, 606. HRMS (ESI) calcd for $C_{26}H_{30}BrN_2O_7$ $[M + H]^+$ 561.1236; found 561.1244.

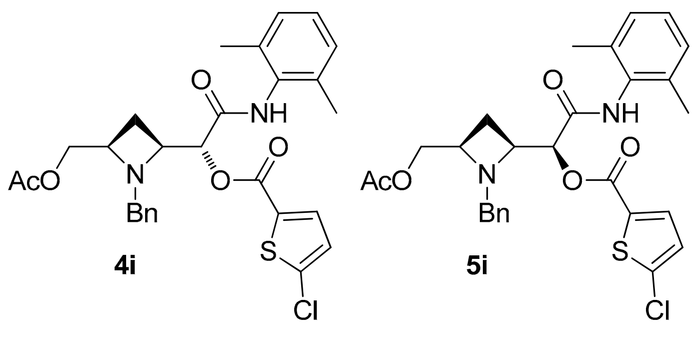

(1R) and (1S) 1-((2S,4R)-4-(Acetoxymethyl)-1-benzylazetidin-2-yl)-2-((2,6-dimethylphenyl)amino)-2-oxoethyl 5-chlorothiophene-2-carboxylates **4i** *and* **5i**

4i: R_f = 0.25, PE/Et_2O 1:1 developed with Hanessian stain; $[\alpha]_D$ = +6.6 (*c* = 0.87, $CHCl_3$); ^{1}H-NMR (300 MHz, $CDCl_3$) δ 7.77 (broad s, 1H; NH), 7.63 (d, *J* = 4.1 Hz, 1H; CH thiophene), 7.34–7.17 (m, 5H; H arom), 7.13–7.00 (m, 3H; H arom), 6.94 (d, *J* = 4.1 Hz, 1H; CH thiophene), 5.32 (d, *J* = 6.2 Hz, 1H; CHO(C=O)), 3.94 and 3.87 (AB part of ABX syst., J_{AB} = 11.5, J_{AX} = 5.0, J_{BX} = 5.8 Hz, 2H; CH_2OAc), 3.90 and 3.75 (AB syst., J_{AB} = 13.0 Hz, 2H; CH_2 of Bn), 3.85–3.78 (m, 1H; H-2), 3.41 (tt, *J* = 8.1, 5.4 Hz, 1H; H-4), 2.33 (dt, *J* = 11.0, 7.8 Hz, 1H; H-3a), 2.18 (s, 6H; 2 CH_3), 2.08 (dt, *J* = 11.0, 8.1 Hz, 1H; H-3b), 1.86 (s, 3H; OAc). ^{13}C-NMR (75 MHz, $CDCl_3$) δ 170.8 (C=O), 165.4 (C=O), 160.1 (C=O), 138.5 (Cq), 137.4 (Cq), 135.2 (2 Cq), 134.1 (CH arom), 132.9 (Cq), 130.3 (Cq), 129.1 (2 CH arom), 128.5 (2 CH arom), 128.3 (2 CH arom), 127.6 (CH arom), 127.5 (CH arom), 127.49 (CH arom), 76.5 (CHO(C=O)), 67.0 (CH_2OAc), 61.6 (CH_2 of Bn), 61.4 (CH-2), 60.2 (CH-4), 23.5 (CH_2-3), 20.6 (OAc), 18.6 (2 CH_3). IR: υ (cm^{-1}) 3270, 3028, 2958, 2870, 1715, 1663, 1629, 1532, 1470, 1454, 1423, 1366, 1335, 1237, 1172, 1088, 1060, 1035, 962, 918, 840, 811, 791, 741. HRMS (ESI) calcd for $C_{28}H_{30}ClN_2O_5S$ $[M + H]^+$ 541.1564; found 541.1551.

5i: R_f = 0.36, PE/Et_2O 1:1 developed with Hanessian stain; $[\alpha]_D$ = −42.6 (*c* = 1.0, $CHCl_3$); ^{1}H-NMR (300 MHz, $CDCl_3$) δ 7.78 (d, *J* = 4.1 Hz, 1H; CH thiophene), 7.54 (broad s, 1H; NH), 7.32–7.20 (m, 5H; H arom), 7.16–7.06 (m, 3H; H arom), 7.05 (d, *J* = 4.1 Hz, 1H; CH thiophene), 5.58 (d, *J* = 2.9 Hz, 1H; CHO(CO)), 3.93 and 3.58 (AB syst., J_{AB} = 13.0 Hz, 2H; CH_2 of Bn), 3.90 (td, *J* = 8.1, 2.9 Hz, 1H; H-2), 3.76 and 3.60 (AB part of ABX syst., J_{AB} = 11.3, J_{AX} = 5.8, J_{BX} = 5.7 Hz, 2H; CH_2OAc), 3.32 (tt, *J* = 7.8, 5.7 Hz, 1H; H-4), 2.30–2.17 (m, 7H; H-3a, 2 CH_3), 2.15–2.04 (m, 1H; H-3b), 1.81 (s, 3H: OAc). ^{13}C-NMR (75 MHz, $CDCl_3$) δ 170.7 (C=O), 165.5 (C=O), 159.4 (C=O), 138.6 (Cq), 137.6 (Cq), 135.2 (2 Cq), 134.1 (CH), 132.6 (Cq), 130.6 (Cq), 129.2 (2 CH), 128.3 (2 CH), 128.3 (2 CH), 127.9 (CH), 127.7 (CH), 127.4 (CH), 73.8 (CHO(C=O)), 67.5 (CH_2OAc), 62.1 (CH-2), 60.4 (CH_2 of Bn), 59.7 (CH-4), 21.9 (CH_2-3), 20.5 (OAc), 18.5 (2 CH_3). HRMS (ESI) calcd for $C_{28}H_{30}ClN_2O_5S$ $[M + H]^+$ 541.1564; found 541.1557.

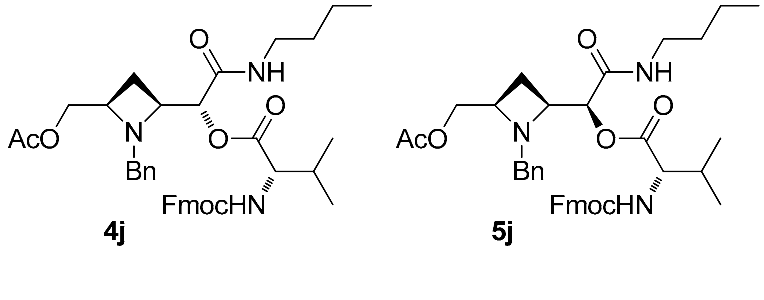

(1R) and (1S) 1-((2S,4R)-4-(Acetoxymethyl)-1-benzylazetidin-2-yl)-2-(butylamino)-2-oxoethyl (2s)-2-((((9h-fluoren-9-yl)methoxy)carbonyl)amino)-3-methylpropanoates **4j** *and* **5j**

4j: R_f = 0.39, PE/Et_2O 3:7 developed with Hanessian stain; $[\alpha]_D$ = −13.6 (*c* = 1.0, $CHCl_3$); ^{1}H-NMR (300 MHz, $CDCl_3$) δ 7.77 (dd, *J* = 7.5, 0.6 Hz, 2H; H arom), 7.57 (dd, *J* = 6.8, 3.0 Hz, 2H; H arom), 7.41 (t, *J* = 7.4 Hz, 2H; H arom), 7.36–7.22 (m, 7H; H arom), 7.07 (broad t, *J* = 5.3 Hz, 1H; NH*n*Bu), 5.36 (broad d, *J* = 8.0 Hz, 1H; N*H*CH), 4.94 (d, *J* = 4.3 Hz, 1H; CHO(C=O)), 4.43 and 4.33 (AB part of ABX syst., J_{AB} = 10.5, J_{AX} = 7.1, J_{BX} = 7.1 Hz, 2H; CH_2 of Fmoc), 4.22 (m, 2H; C*H*NH, CH of Fmoc), 3.78 and 3.58

(AB syst., J_{AB} = 13.4 Hz, 2H; CH_2 of Bn), 3.92–3.66 (m, 3H; CH_2OAc, H-2), 3.38–3.02 (m, 3H; CH_2 of *n*Bu, H-4), 2.24–2.11 (m, 2H; CH of *i*Pr, H-3a), 1.89 (s, 3H; OAc), 1.87–1.74 (m, 1H; H-3b), 1.50–1.38 (m, 2H; CH_2 of *n*Bu), 1.36–1.20 (m, 2H; CH_2 of *n*Bu), 1.02 (t, *J* = 6.4 Hz, 6H; 2 CH_3 of *i*Pr), 0.83 (t, *J* = 7.2 Hz, 3H; CH_3 of *n*Bu). ^{13}C-NMR (75 MHz, $CDCl_3$) δ 171.7 (C=O), 170.7 (C=O), 167.0 (C=O), 156.6 (C=O), 143.6 (2 Cq), 141.3 (Cq), 141.2 (Cq), 137.5 (Cq), 129.2 (2 CH arom), 128.4 (2 CH arom), 127.8 (2 CH arom), 127.5 (CH arom), 127.1 (CH arom), 127.1 (CH arom), 125.0 (CH arom), 124.9 (CH arom), 120.0 (2 CH arom), 75.2 (CHO(C=O)), 67.3 (CH2 of Fmoc), 67.3 (CH_2OAc), 61.3 (CH_2 of Bn), 60.9 (CH-2), 60.0 (CH of Fmoc), 59.8 (CH-4), 47.1 (CHNH), 39.1 (CH_2 of *n*Bu), 31.3 (CH_2 of *n*Bu), 30.5 (CH of *i*Pr), 23.1 (CH_2-3), 20.7 (OAc), 20.1 (CH_2 of *n*Bu), 19.4 (CH_3 of *i*Pr), 18.2 (CH_3 of *i*Pr), 13.7 (CH_3 of *n*Bu). IR: υ (cm^{-1}) 3344, 2961, 2933, 2873, 1742, 1706, 1657, 1533, 1496, 1478, 1466, 1451, 1373, 1347, 1286, 1234, 1183, 1155, 1094, 1029, 909, 787, 758, 739. HRMS (ESI) calcd for $C_{39}H_{48}N_3O_7$ $[M + H]^+$ 670.3492; found 670.3502.

5j: R_f = 0.50, PE/Et_2O 3:7 developed with Hanessian stain; $[\alpha]_D$ = −41.9 (*c* = 0.76, $CHCl_3$); ^{1}H-NMR (300 MHz, $CDCl_3$) δ 7.77 (d, *J* = 7.5 Hz, 2H; H arom), 7.58 (d, *J* = 7.4 Hz, 2H; H arom), 7.40 (t, *J* = 7.2 Hz, 2H; H arom), 7.35–7.19 (m, 7H; H arom), 6.67 (broad t, *J* = 5.3 Hz, 1H; NH*n*Bu), 5.55 (broad d, *J* = 7.9 Hz, 1H; N*H*CH), 5.12 (d, *J* = 3.0 Hz, 1H; CHO(C=O)), 4.49 (dd, *J* = 10.3, 6.9 Hz, 1H; 1 H of CH_2 of Fmoc), 4.36–4.25 (m, 2H; 1 H of CH_2 of Fmoc, C*H*NH), 4.21 (t, *J* = 6.9 Hz, 1H; CH of Fmoc), 3.87–3.68 (m, 3H; CH_2OAc, H-2), 3.77 and 3.57 (AB syst., J_{AB} = 12.9 Hz, 2H; CH_2 of Bn), 3.28 (qd, *J* = 7.8, 6.2 Hz, 1H; H-4), 3.18 (dd, *J* = 12.9, 7.0 Hz, 2H; CH_2 of *n*Bu), 2.46–2.30 (m, 1H; CH of *i*Pr), 2.12 (dt, *J* = 10.9, 8.1 Hz, 1H; H-3a), 1.84 (s, 3H; OAc), 1.78 (dt, *J* = 10.9, 7.9 Hz, 1H; H-3b), 1.48–1.36 (m, 2H; CH_2 of *n*Bu), 1.34–1.22 (m, 2H; CH_2 of *n*Bu), 1.09 (d, *J* = 6.9 Hz, 3H; CH_3 of *i*Pr), 1.02 (d, *J* = 6.9 Hz, 3H; CH_3 of *i*Pr), 0.85 (t, *J* = 7.4 Hz, 3H; CH_3 of *n*Bu). IR: υ (cm^{-1}) 3329, 2960, 2933, 2873, 1739, 1718, 1657, 1532, 1478, 1466, 1451, 1366, 1317, 1271, 1231, 1186, 1169, 1092, 1031, 915, 758, 739. HRMS (ESI) calcd for $C_{39}H_{48}N_3O_7$ $[M + H]^+$ 670.3492; found 670.3498.

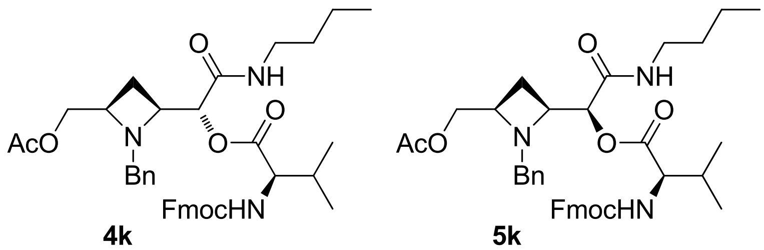

4k **5k**

(1R) and (1S) 1-((2S,4R)-4-(Acetoxymethyl)-1-benzylazetidin-2-yl)-2-(butylamino)-2-oxoethyl (2r)-2-((((9h-fluoren-9-yl)methoxy)carbonyl)amino)-3-methylpropanoates **4k** *and* **5k**

4k: R_f = 0.31, PE/Et_2O 3:7 developed with Hanessian stain; $[\alpha]_D$ = +1.87 (*c* = 1.0, $CHCl_3$); ^{1}H-NMR (300 MHz, $CDCl_3$) δ 7.78 (dd, *J* = 7.4, 4.1 Hz, 2H; H arom), 7.60 (dd, *J* = 7.4, 3.5 Hz, 2H; H arom), 7.49–7.27 (m, 5H; H arom), 7.24–7.13 (m, 5H; 4 H arom and NHDragendorff), 5.54 (d, *J* = 8.3 Hz, 1H; N*H*CH), 4.88 (d, *J* = 4.1 Hz, 1H; CHO(C=O)), 4.57–4.49 (m, 1H; 1 H of CH_2 of Fmoc), 4.34–4.19 (m, 3H; 1 H of CH_2 of Fmoc, C*H*NH and CH of Fmoc), 4.02 (dd, *J* = 11.2, 6.1 Hz, 1H; 1 H of CH_2OAc), 3.69 and 3.58 (AB syst., J_{AB} = 13.0 Hz, 2H; CH_2 of Bn), 3.70–3.59 (m, 2H; H-2 and 1 H of CH_2OAc), 3.37–3.18 (m, 3H; H-4 and C*H*$_2$NH), 2.42–2.32 (m, 1H; CH of *i*Pr), 2.25 (dt, *J* = 11.3, 8.2 Hz, 1H; H-3a), 1.92 (s, 3H; OAc), 1.75 (dt, *J* = 11.4, 8.0 Hz, 1H; H-3b), 1.57–1.45 (m, 2H; CH_2 of *n*Bu), 1.41–1.25 (m, 2H; CH_2 of *n*Bu), 1.06 (d, *J* = 6.7 Hz, 3H; CH_3 of *i*Pr), 0.98 (d, *J* = 6.9 Hz, 3H; CH_3 of *i*Pr), 0.90 (t, *J* = 7.4 Hz, 3H; CH_3 of *n*Bu). ^{13}C-NMR (75 MHz, $CDCl_3$) δ 170.85 (C=O), 170.82 (C=O), 167.0 (C=O), 157.1 (C=O), 143.7 (Cq), 143.7 (Cq), 141.3 (Cq), 141.2 (Cq), 137.6 (Cq), 128.9 (2 CH arom), 128.4 (CH arom), 127.80 (CH arom), 127.76 (CH arom), 127.72 (CH arom), 127.5 (CH arom), 127.1 (CH arom), 127.0 (CH arom), 125.1 (CH arom), 125.0 (CH arom), 120.00 (CH arom), 119.96 (CH arom), 74.3 (CHO(C=O)), 67.2 (CH_2 of Fmoc), 66.9 (CH_2OAc), 61.7 (CH_2 of Bn), 61.4 (CH-2), 59.8 (CH-4), 59.4 (CH of Fmoc), 47.1 (CHNH), 39.1 (CH_2 of *n*Bu), 31.4 (CH_2 of *n*Bu), 30.4 (CH of *i*Pr), 22.6 (CH_2-3), 20.7 (OAc), 20.1 (CH_2 of *n*Bu), 19.2 (CH_3 of *i*Pr), 17.3 (CH_3 of *i*Pr), 13.7 (CH_3 of *n*Bu). IR: υ (cm^{-1}) 3329, 2960, 2932, 2873, 1739, 1704, 1656, 1533, 1478,

1466, 1451, 1373, 1318, 1233, 1191, 1134, 1096, 1030, 909, 786, 759, 738. HRMS (ESI) calcd for $C_{39}H_{48}N_3O_7$ $[M + H]^+$ 670.3492; found 670.3487.

5k: R_f = 0.40, PE /Et_2O 3:7 developed with Hanessian stain; $[\alpha]_D$ = −21.8 (*c* = 0.4, $CHCl_3$); ^{1}H-NMR (300 MHz, $CDCl_3$) δ 7.77 (d, *J* = 7.5 Hz, 2H; H arom), 7.56 (dd, *J* = 7.4, 4.3 Hz, 2H; H arom), 7.41 (t, *J* = 7.5 Hz, 2H; H arom), 7.37–7.17 (m, 7H; H arom), 6.74 (broad t, *J* = 5.5 Hz, 1H; NH*n*Bu), 5.28 (d, *J* = 7.5 Hz, 1H; N*H*CH), 5.12 (d, *J* = 2.5 Hz, 1H; CHO(C=O)), 4.42 and 4.34 (AB part of ABX syst., J_{AB} = 10.5, J_{AX} = 7.1, J_{BX} = 7.2 Hz, 2H; CH_2 of Fmoc), 4.21 (t, *J* = 7.0 Hz, 1H; CH of Fmoc), 4.10 (t, *J* = 7.4 Hz, 1H; NHC*H*), 3.86–3.67 (m, 3H; H-2, CH_2OAc), 3.78 and 3.58 (AB syst., J_{AB} = 12.8 Hz, 2H; CH_2 of Bn), 3.33–3.15 (m, 2H; H-4, 1 H of C*H*$_2$NH), 3.15–3.00 (m, 1H; 1 H of C*H*$_2$NH), 2.22–2.03 (m, 2H; CH of *i*Pr, H-3a), 1.97–1.81 (m, 1H; H-3b), 1.89 (s, 3H; OAc), 1.49–1.36 (m, 2H; CH_2 of *n*Bu), 1.33–1.21 (m, 2H; CH_2 of *n*Bu), 1.13 (d, *J* = 6.8 Hz, 3H; CH_3 of *i*Bu), 1.09 (d, *J* = 6.8 Hz, 3H; CH_3 of *i*Bu), 0.82 (t, *J* = 7.2 Hz, 3H; CH_3 of *n*Bu). ^{13}C-NMR (75 MHz, $CDCl_3$) δ 171.7 (C=O), 170.8 (C=O), 167.0 (C=O), 156.7 (C=O), 143.6 (Cq), 143.5 (Cq), 141.3 (Cq), 141.2 (Cq), 137.8 (Cq), 129.3 (2 CH arom), 128.2 (2 CH arom), 127.8 (2 CH arom), 127.3 (CH arom), 127.2 (CH arom), 127.1 (CH arom), 124.9 (CH arom), 124.9 (CH arom), 120.1 (CH arom), 120.0 (CH arom), 73.7 (CHO(C=O)), 67.7 (CH_2OAc), 67.3 (CH_2 of Fmoc), 62.2 (CH-2), 61.2 (CH_2 of Bn), 60.4 (CHNH), 59.8 (CH-4), 47.1 (CH of Fmoc), 39.1 (CH_2 of *n*Bu), 31.3 (CH_2 of *n*Bu), 30.3 (CH of *i*Bu), 21.7 (CH_2-3), 20.7 (OAc), 20.0 (CH_2 of *n*Bu), 19.5 (CH_3 of *i*Bu), 18.6 (CH_3 of *i*Bu), 13.7 (CH_3 of *n*Bu). IR: υ (cm^{-1}) 3354, 2961, 2933, 2874, 1743, 1705, 1656, 1535, 1496, 1478, 1466, 1451, 1373, 1286, 1236, 1183, 1155, 1085, 1030, 918, 790, 757, 739. HRMS (ESI) calcd for $C_{39}H_{48}N_3O_7$ $[M + H]^+$ 670.3492; found 670.3490.

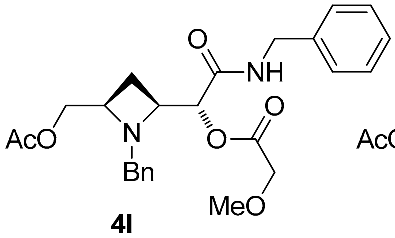

4l

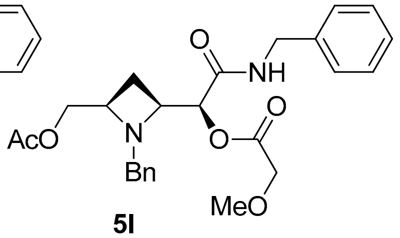

5l

(1R) and (1S) 1-((2S,4R)-4-(Acetoxymethyl)-1-benzylazetidin-2-yl)-2-(benzylamino)-2-oxoethyl 2-methoxyacetate **4l** *and* **5l**

4l: R_f = 0.26, PE/Et_2O 1:5 developed with Hanessian stain; $[\alpha]_D$ = −23.1 (*c* = 1.7, $CHCl_3$); ^{1}H-NMR (300 MHz, $CDCl_3$) δ 7.39–7.11 (m, 11H; 10H arom and NH), 4.92 (d, *J* = 4.7 Hz, 1H; CHO(C=O)), 4.52–4.34 (m, 2H; CH_2NH), 4.07 and 3.97 (AB syst., J_{AB} = 16.6 Hz, 2H; CH_2 of Bn), 3.80 and 3.74 (AB part of ABX syst., J_{AB} = 11.1, J_{AX} = 5.0, J_{BX} = 6.1 Hz, 2H; CH_2OAc), 3.65 (s, 2H; C*H*$_2$OMe), 3.64–3.54 (m, 1H; H-2), 3.42 (s, 3H; OMe), 3.40–3.28 (m, 1H; H-4), 2.23 (dt, *J* = 11.2, 7.9 Hz, 1H; H-3a), 1.92 (s, 3H; OAc), 1.76 (dt, *J* = 11.2, 8.4 Hz, 1H; H-3b). ^{13}C-NMR (75 MHz, $CDCl_3$) δ 170.6 (C=O), 169.3 (C=O), 166.8 (C=O), 137.7 (Cq), 137.3 (Cq), 128.9 (2 CH arom), 128.7 (2 CH arom), 128.6 (2 CH arom), 128.0 (2 CH arom), 127.6 (2 CH arom), 73.5 (CHO(C=O)), 69.4 (CH_2 of Bn), 66.9 (CH_2OAc), 61.6 (CH_2OMe), 61.6 (CH-2), 60.3 (CH-4), 59.4 (OMe), 43.4 (CH_2NH), 23.0 (CH_2-3), 20.7 (OAc). IR: υ (cm^{-1}) 3315, 3064, 3029, 3004, 2935, 2883, 2829, 1739, 1666, 1605, 1533, 1496, 1454, 1366, 1333, 1233, 1183, 1125, 1029, 976, 932, 910, 820, 750, 732. HRMS (ESI) calcd for $C_{25}H_{31}N_2O_6$ $[M + H]^+$ 455.2182; found 455.2171.

5l: R_f = 0.34, PE/Et_2O 1:5 developed with Hanessian stain; $[\alpha]_D$ = −28.8 (*c* = 0.6, $CHCl_3$); ^{1}H-NMR (300 MHz, $CDCl_3$) δ 7.39–7.16 (m, 10H; H arom), 6.51 (broad t, *J* = 5.7 Hz, 1H; NH), 5.37 (d, *J* = 3.4 Hz, 1H; CHO(C=O)), 4.51 and 4.40 (AB part of ABX syst., J_{AB} = 14.8, J_{AX} = 6.0, J_{BX} = 5.7 Hz, 2H; C*H*$_2$NH), 4.10 (s, 2H; C*H*$_2$OMe), 3.79 and 3.52 (AB syst., J_{AB} = 12.9 Hz, 2H; CH_2 of Bn), 3.75–3.63 (m, 3H; CH_2OAc and H-2), 3.46 (s, 3H; OMe), 3.30 (tt, *J* = 7.9, 5.6 Hz, 1H; H-4), 2.14 (dt, *J* = 10.8, 7.9 Hz, 1H; H-3a), 1.87 (s, 3H; OAc), 1.87 (dt, *J* = 10.9, 8.3 Hz, 1H; H-3b). ^{13}C-NMR (75 MHz, $CDCl_3$) δ 170.7 (C=O), 168.8 (C=O), 167.1 (C=O), 137.8 (Cq), 137.6 (Cq), 129.2 (2 CH arom), 128.8 (2 CH arom), 128.3 (2 CH arom), 127.8 (2 CH arom), 127.7 (CH arom), 127.4 (CH arom), 73.6 (CHO(C=O)), 69.8 (CH_2OMe), 67.4 (CH_2OAc),

62.1 (CH-2), 60.8 (CH_2 of Bn), 59.7 (CH-4), 59.5 (CH_3O), 43.2 (CH_2NH), 21.60 (CH_2-3), 20.7 (OAc). IR: υ (cm^{-1}) 3339, 3063, 3029, 2936, 2883, 2828, 1737, 1658, 1605, 1530, 1496, 1454, 1366, 1333, 1235, 1179, 1124, 1086, 1029,976, 927,910, 849, 733. HRMS (ESI) calcd for $C_{25}H_{31}N_2O_6$ $[M + H]^+$ 455.2182; found 455.2174.

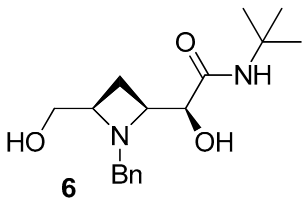

6

(1S)-2-((2S,4R)-1-Benzyl-4-(hydroxymethyl)azetidin-2-yl)-N-(tert-butyl)-2-hydroxyacetamide **6**: R_f = 0.36, *n*Hex/Et_2O 1:6 developed with Dragendorff stain; m.p. = 123.0–128.8 °C (AcOEt); $[\alpha]_D$ = −74.8 (*c* = 1.1, $CHCl_3$); ^{1}H-NMR (400 MHz, $CDCl_3$): δ 7.32–7.26 (m, 5H; CH arom), 6.58 (s, 1H; NH), 4.01 (s, 1H; OH), 3.76–3.69 (m, 3H; CH-2 and CH_2 of Bn), 3.58 (d, *J* = 3.7 Hz, 1H; CHCON), 3.42–3.35 (m, 2H; CH_2OH), 3.32 (tt, *J* = 7.8, 3.8 Hz, 1H; CH-4), 1.98–1.80 (m, 3H, CH_2-3 and OH), 1.31 (s, 9H; *t*Bu); ^{13}C-NMR (100 MHz, $CDCl_3$): δ 169.6 (C=O), 137.4 (Cq), 129.0 (CH arom), 128.7 (CH arom), 127.8 (CH arom), 70.1 (CHOH), 64.2 (CH_2OH), 63.1 (CH-2), 62.7 (CH-4), 60.3 (CH_2 of Bn), 50.8 (Cq), 28.7 (*t*Bu), 17.9 (CH_2-3). IR: υ (cm^{-1}) 3483, 3457, 3383, 2990, 2963, 2940, 2901, 2874, 2832, 1654, 1536, 1496, 1478, 1453, 1393, 1382, 1371, 1339, 1328, 1302, 1274, 1252, 1226, 1195, 1172, 1153, 1134, 1114, 1094, 1044, 1028, 995, 958, 931, 906, 893, 850, 793, 756, 732. HRMS (ESI) calcd for $C_{17}H_{27}N_2O_3$ $[M + H]^+$ 307.2022; found 307.2031.

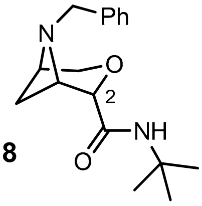

8

(1S,2S,5R)-6-Benzyl-N-(tert-butyl)-3-oxa-6-azabicyclo[3.1.1]heptane-2-carboxamide **8**: R_f = 0.28 (ETP/Et_2O 1:3); ^{1}H-NMR (300 MHz, $CDCl_3$) δ 7.46–7.14 (m, 5H; H arom), 6.71 (broad s, 1H; NH), 4.54 (d, *J* = 1.5 Hz, 1H; H-2), 4.30 and 3.82 (AB part of ABX syst., J_{AB} = 10.6, J_{AX} = 1.1, J_{BX} = 1.8 Hz, 2H; CH_2-6), 3.89 (ddd, *J* = 5.6, 3.5, 1.7 Hz, 1H; H-3), 3.84 (s, 2H; CH_2 of Bn), 3.55–3.44 (m, 1H; H-5), 2.60 (dt, *J* = 8.5, 6.2 Hz, 1H; H-4a), 1.62 (d, *J* = 8.8 Hz, 1H; H-4b), 1.37 (s, 9H; *t*Bu). ^{13}C-NMR (100 MHz, $CDCl_3$): δ 170.2 (C=O), 137.7 (Cq), 128.5 (2 CH arom), 128.3 (2 CH arom), 127.0 (CH arom), 71.1 (CH-2), 63.2 (CH_2-6), 62.5 (H-3), 59.7 (H-4), 50.8 (Cq), 49.9 (CH_2 of Bn), 28.8 (*t*Bu), 26.1 (CH_2-4).

3. Copies of ^{1}H- and ^{13}C-NMR Spectra of Compounds

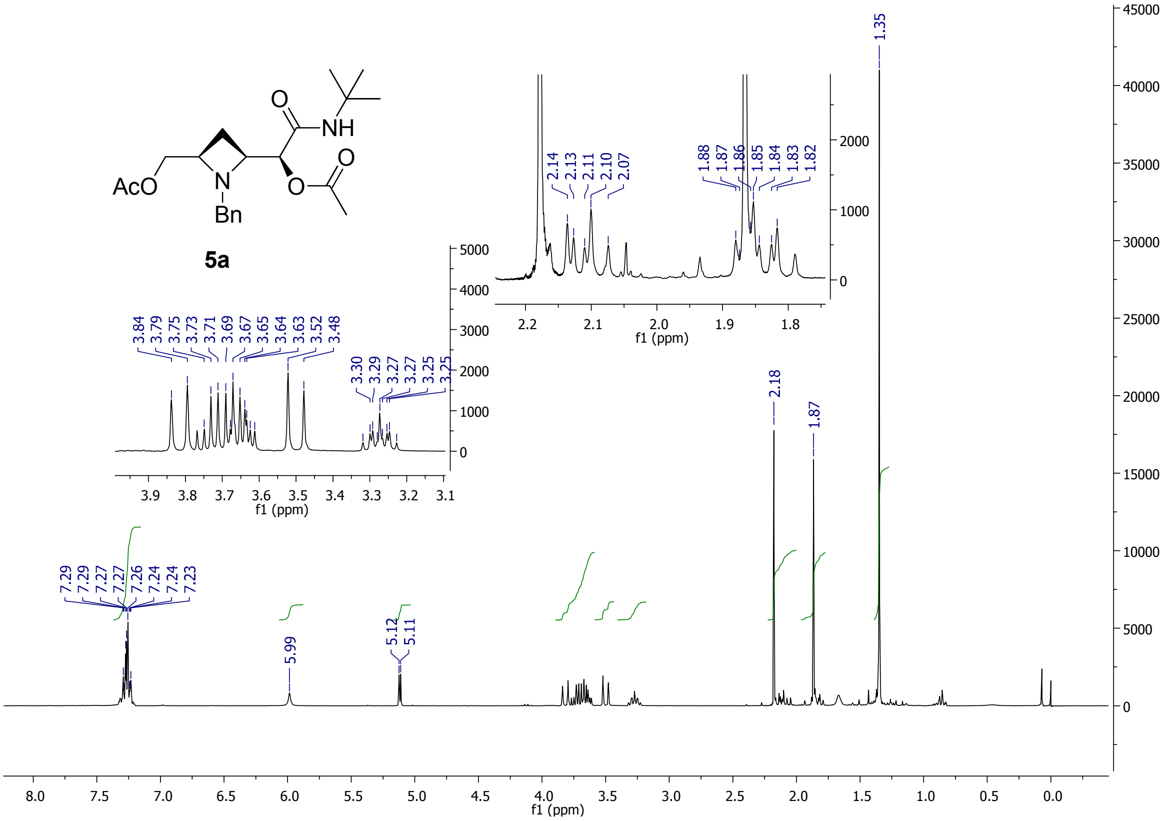

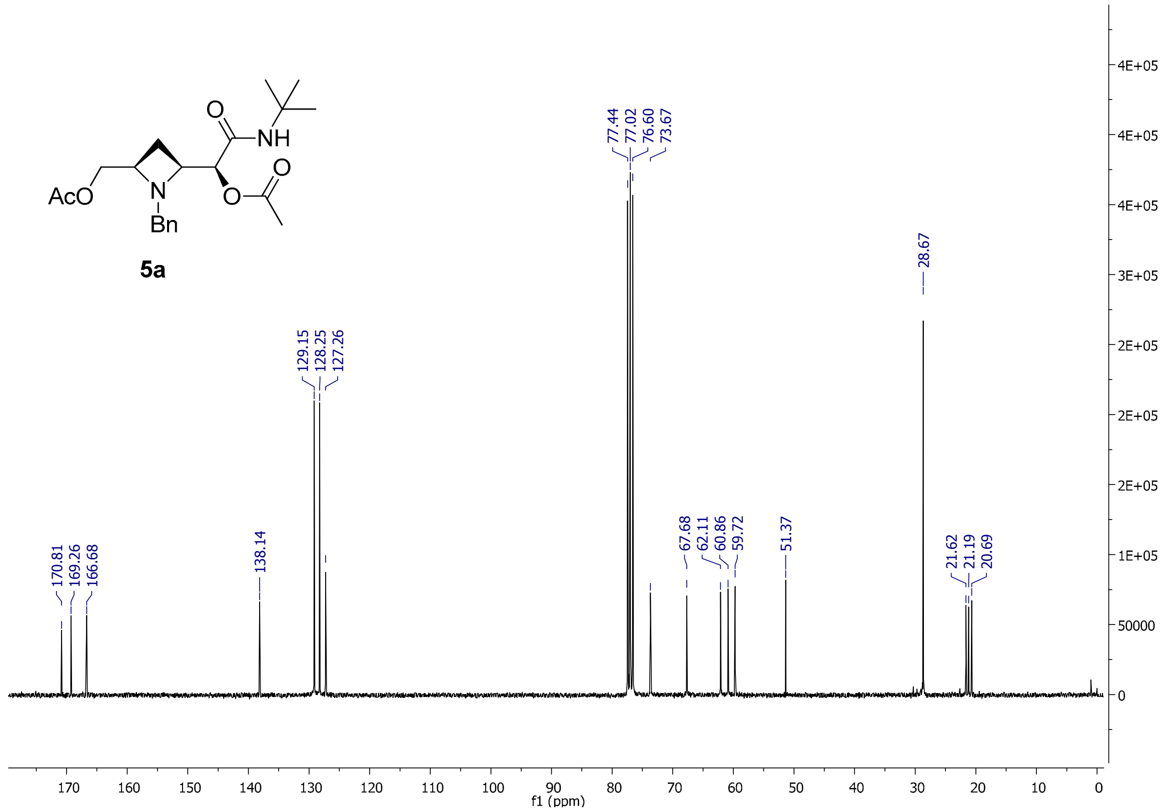

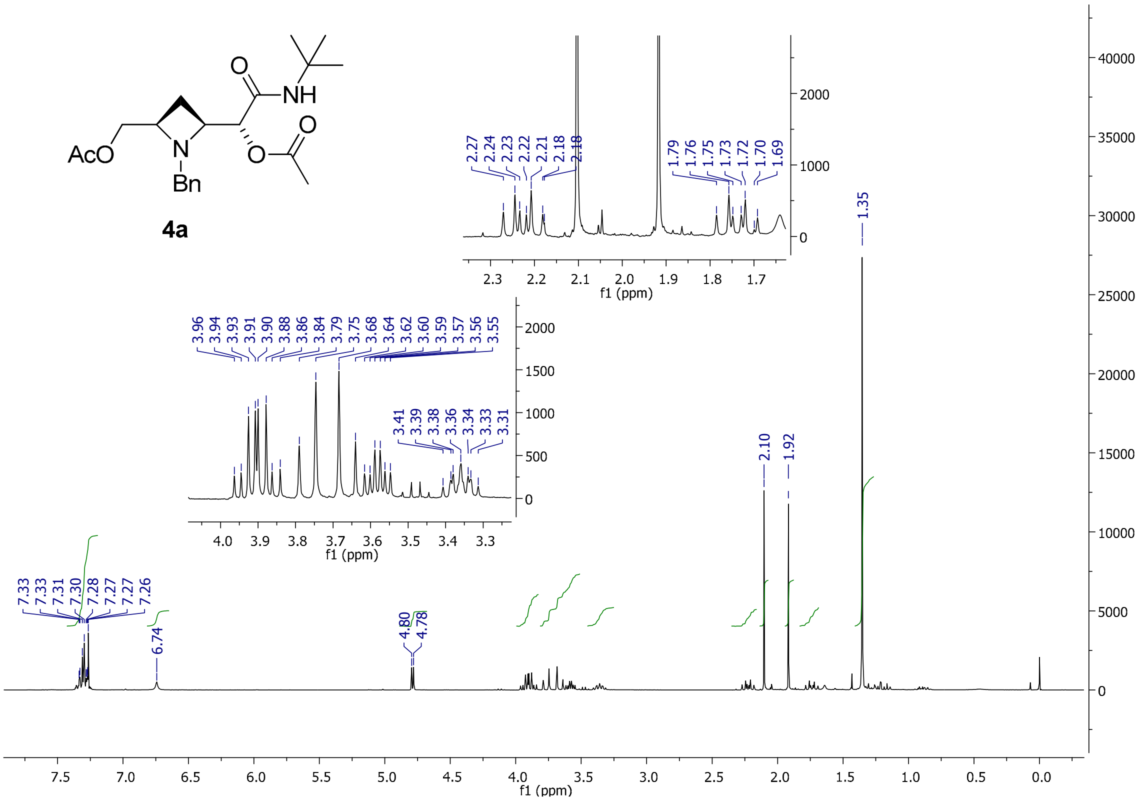
AcO
N
Bn
O
NH
4a
7.33 7.33 7.31 7.30 7.28 7.27 7.27 7.26
6.74
4.80 4.78
2.10
1.92
1.35
3.96 3.94 3.93 3.91 3.90 3.88 3.86 3.84 3.79 3.75 3.68 3.64 3.62 3.60 3.59 3.57 3.56 3.55
3.41 3.39 3.38 3.36 3.34 3.33 3.31
2.27 2.24 2.23 2.22 2.21 2.18 2.18
1.79 1.76 1.75 1.73 1.72 1.70 1.69
f1 (ppm)

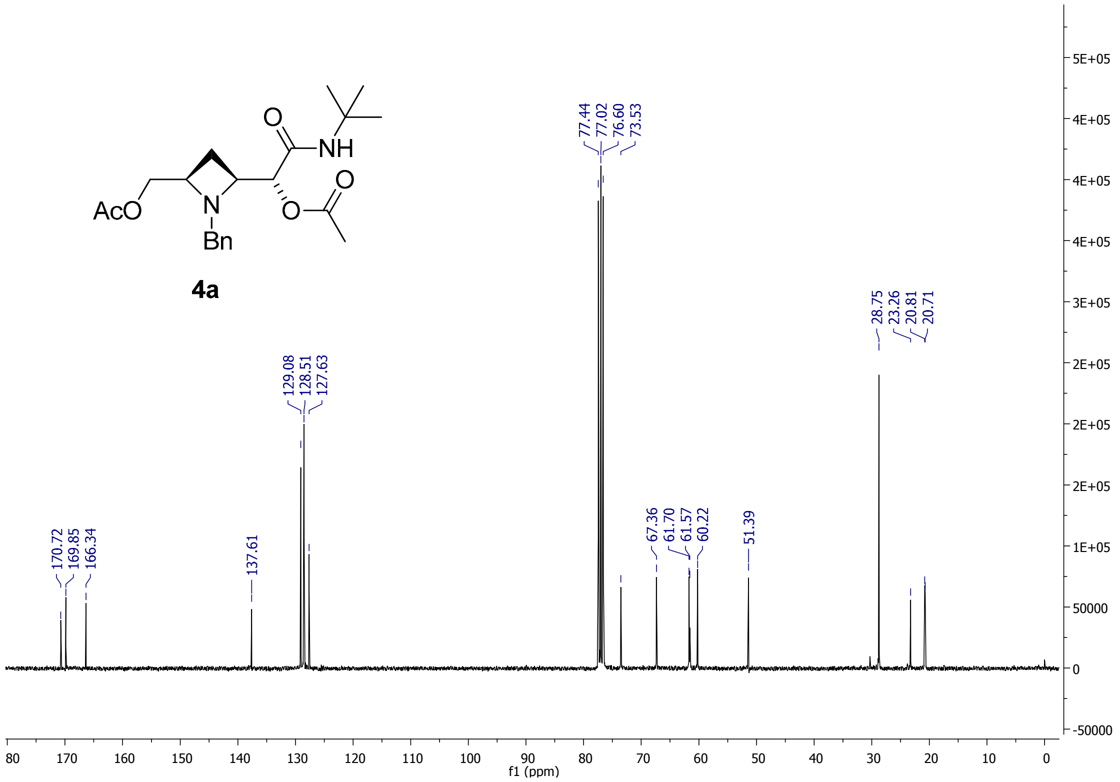
AcO
N
Bn
O
NH
4a
170.72 169.85 166.34
137.61
129.08 128.51 127.63
77.44 77.02 76.60 73.53
67.36 61.70 61.57 60.22
51.39
28.75 23.26 20.81 20.71
f1 (ppm)

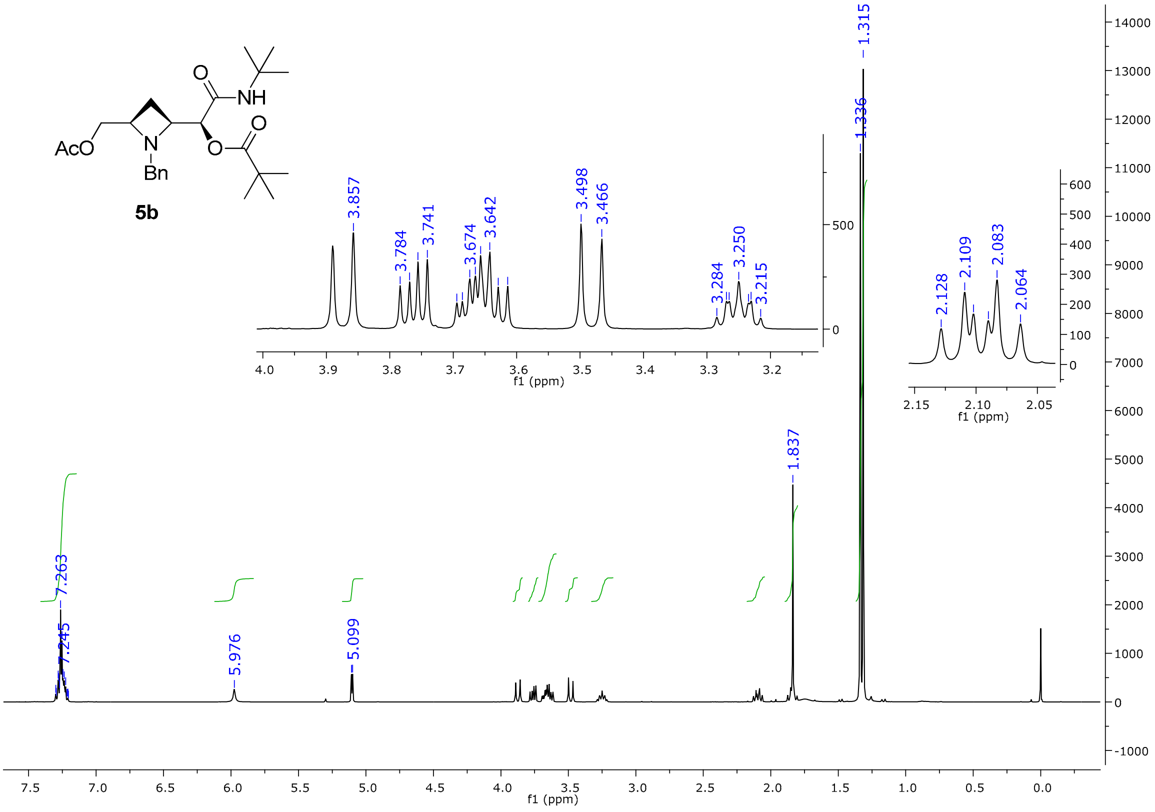
5b
AcO
N
Bn
O
NH
O
O
f1 (ppm)

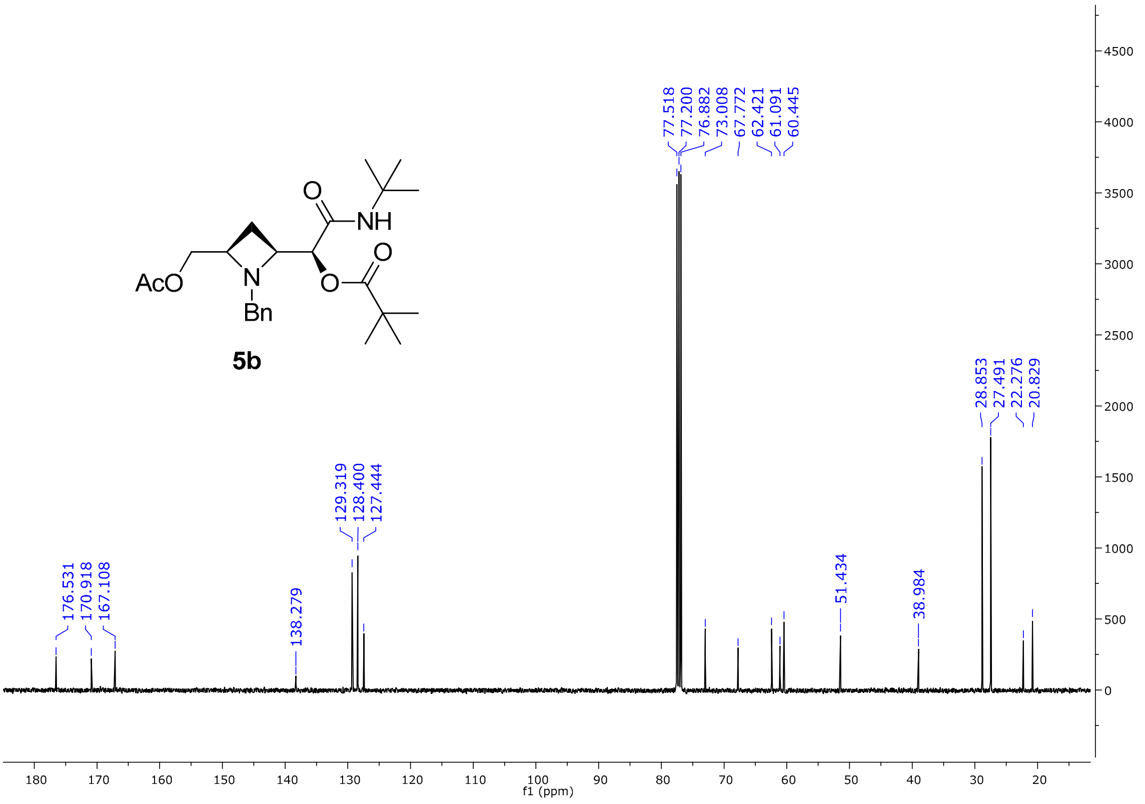
5b
AcO
N
Bn
O
NH
O
O
f1 (ppm)

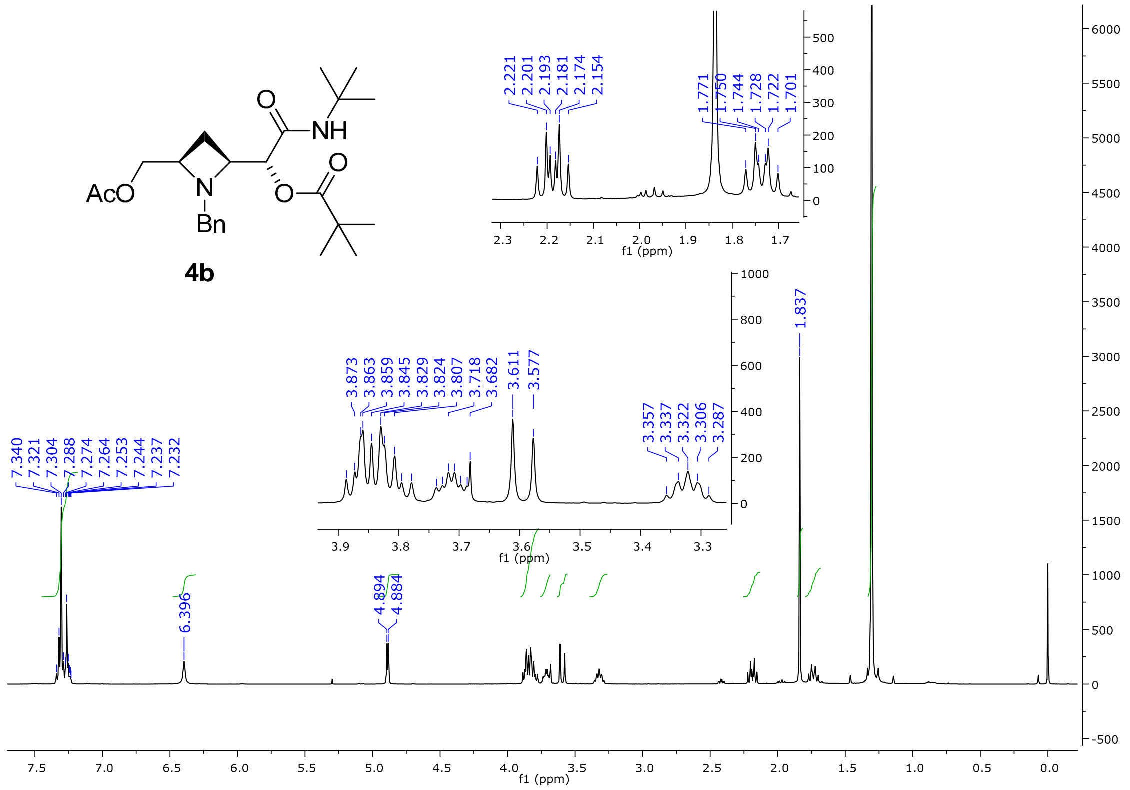
AcO
N
Bn
NH
O
O
4b
7.340
7.321
7.304
7.288
7.274
7.264
7.253
7.244
7.237
7.232
6.396
4.894
4.884
3.873
3.863
3.859
3.845
3.829
3.824
3.807
3.718
3.682
3.611
3.577
3.357
3.337
3.322
3.306
3.287
2.221
2.201
2.193
2.181
2.174
2.154
1.771
1.750
1.744
1.728
1.722
1.701
1.837
f1 (ppm)

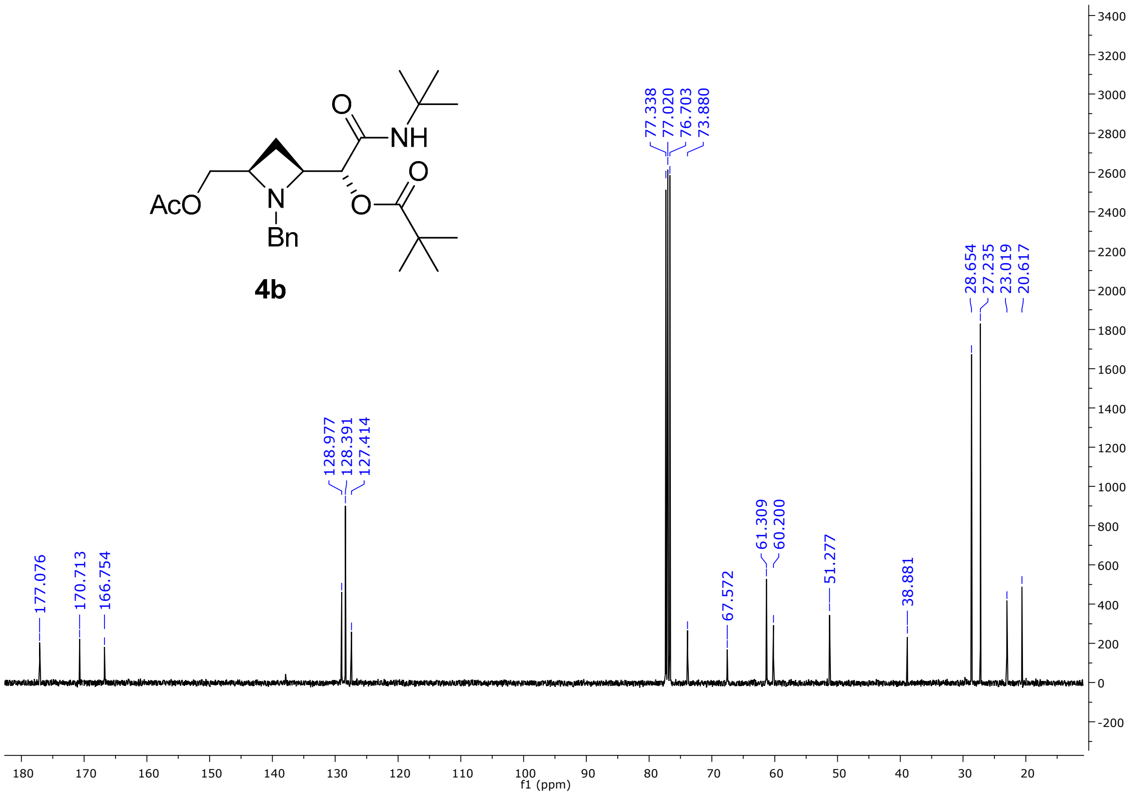
AcO
N
Bn
NH
O
O
4b
177.076
170.713
166.754
128.977
128.391
127.414
77.338
77.020
76.703
73.880
67.572
61.309
60.200
51.277
38.881
28.654
27.235
23.019
20.617
f1 (ppm)

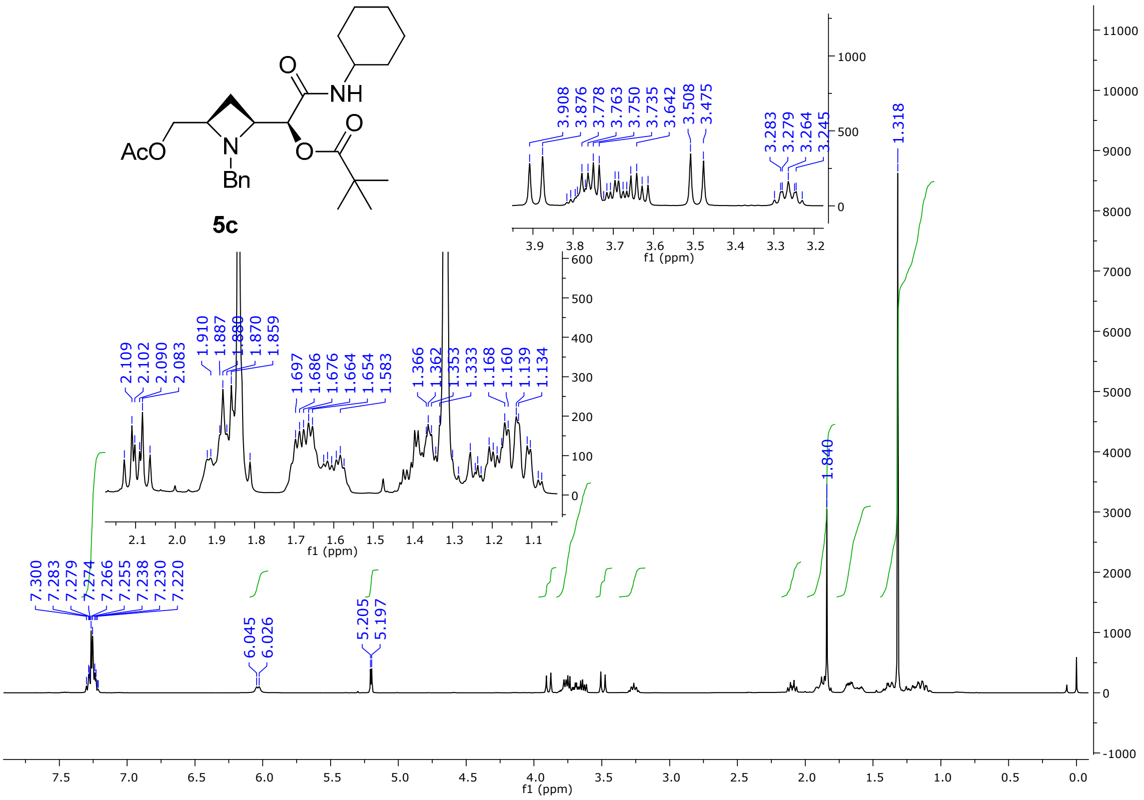

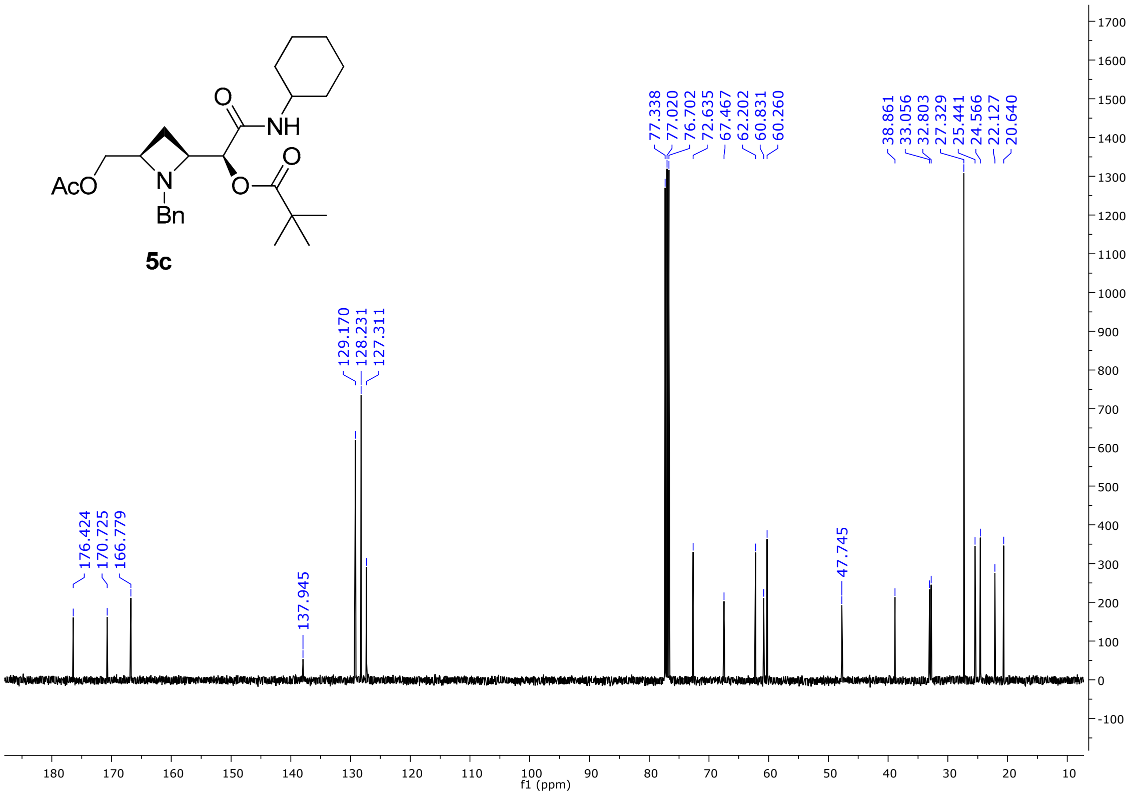

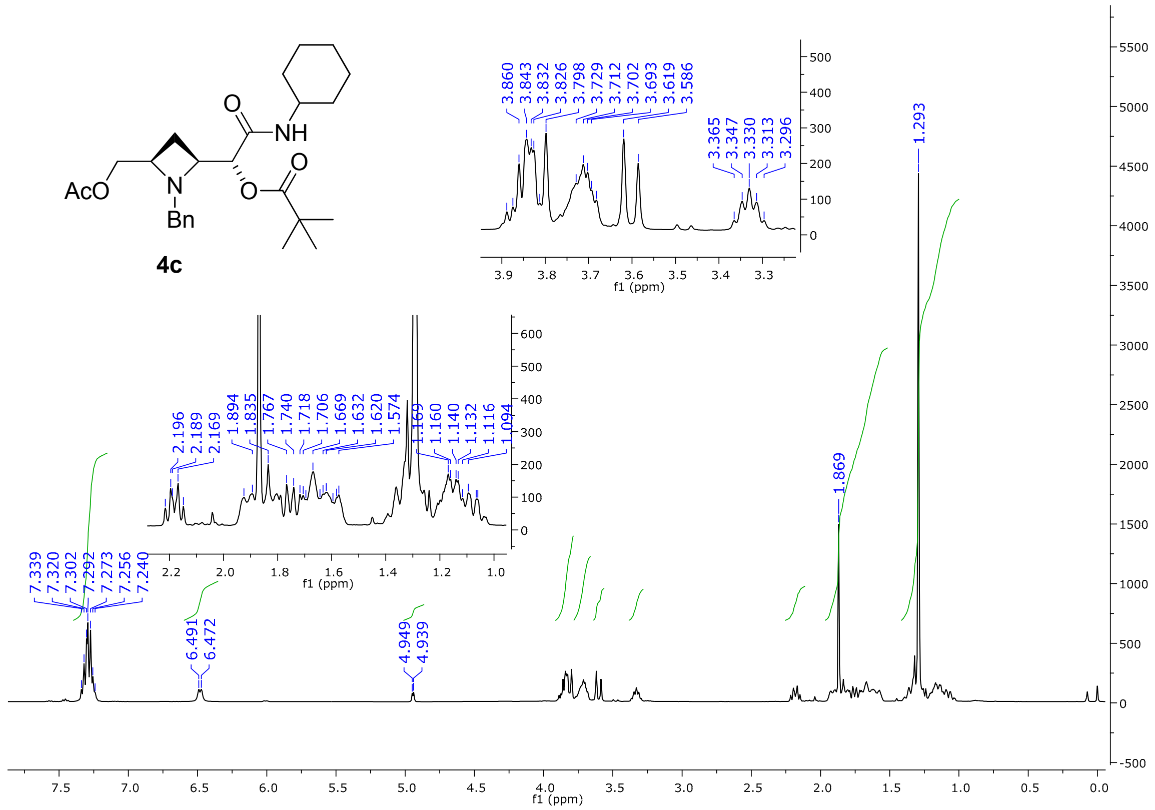

AcO
N
Bn
O
NH
O
4c
3.860 3.843 3.832 3.826 3.798 3.729 3.712 3.702 3.693 3.619 3.586
3.365 3.347 3.330 3.313 3.296
1.293
2.196 2.189 2.169 1.894 1.835 1.767 1.740 1.718 1.706 1.669 1.632 1.620 1.574 1.169 1.160 1.140 1.132 1.116 1.094
1.869
7.339 7.320 7.302 7.292 7.273 7.256 7.240
6.491 6.472
4.949 4.939
f1 (ppm)

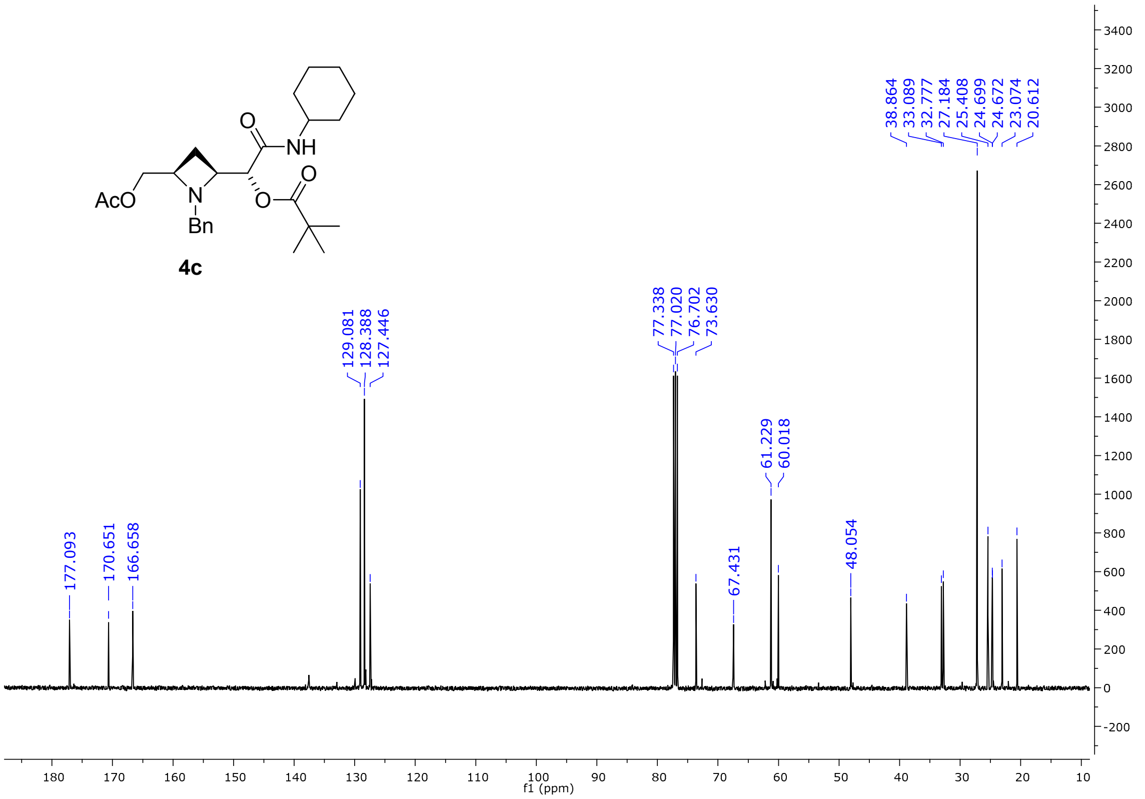

AcO
N
Bn
O
NH
O
4c
38.864 33.089 32.777 27.184 25.408 24.699 24.672 23.074 20.612
129.081 128.388 127.446
77.338 77.020 76.702 73.630
61.229 60.018
177.093
170.651
166.658
67.431
48.054
f1 (ppm)

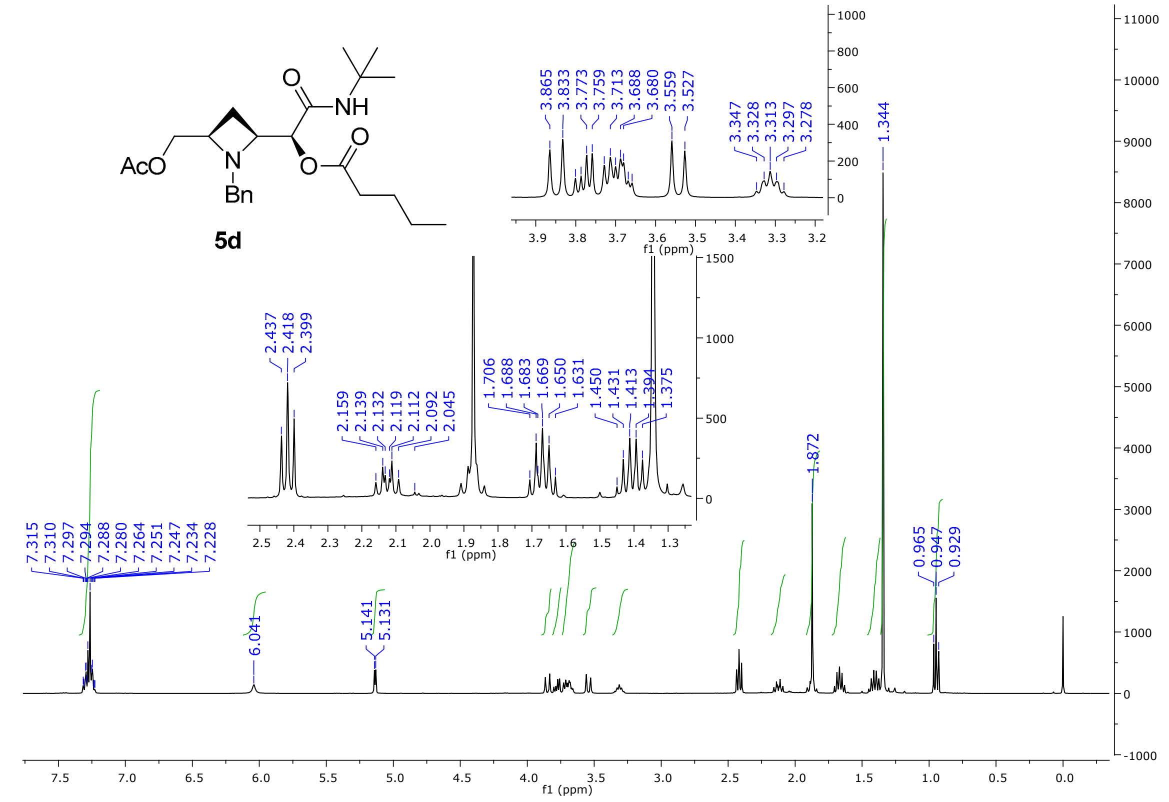

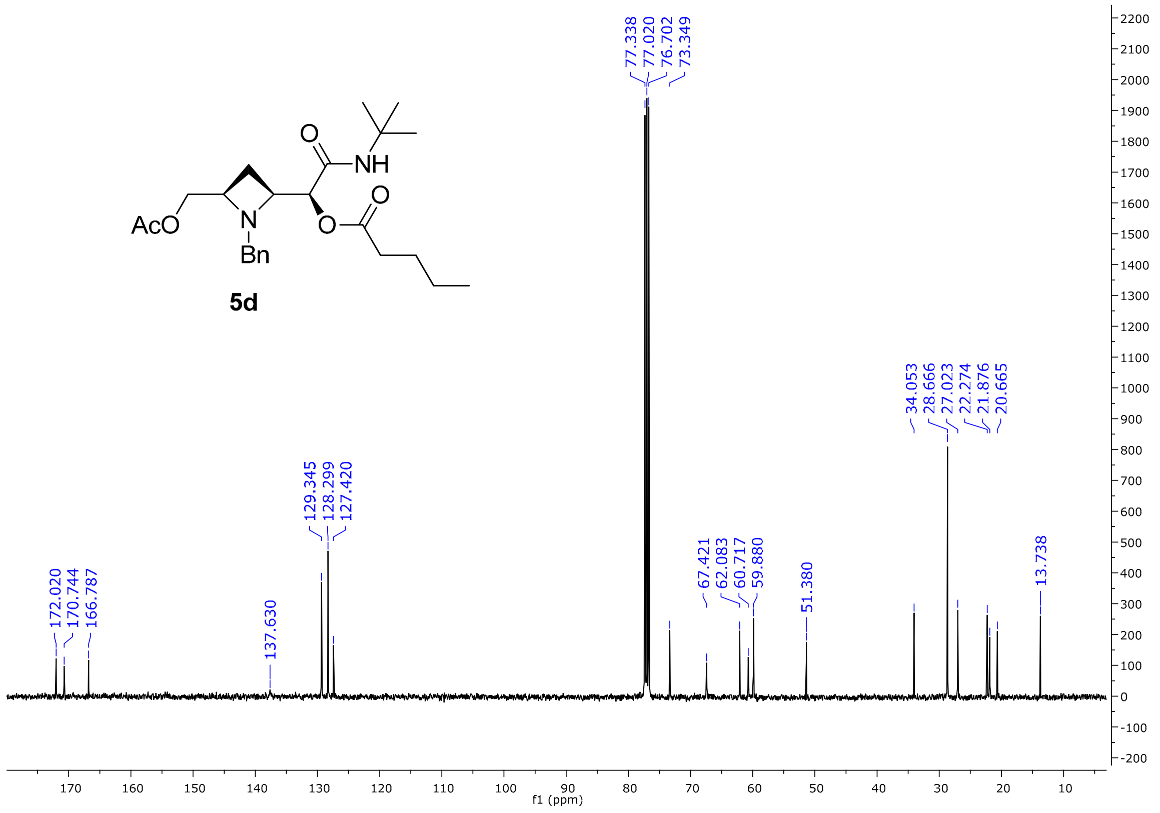

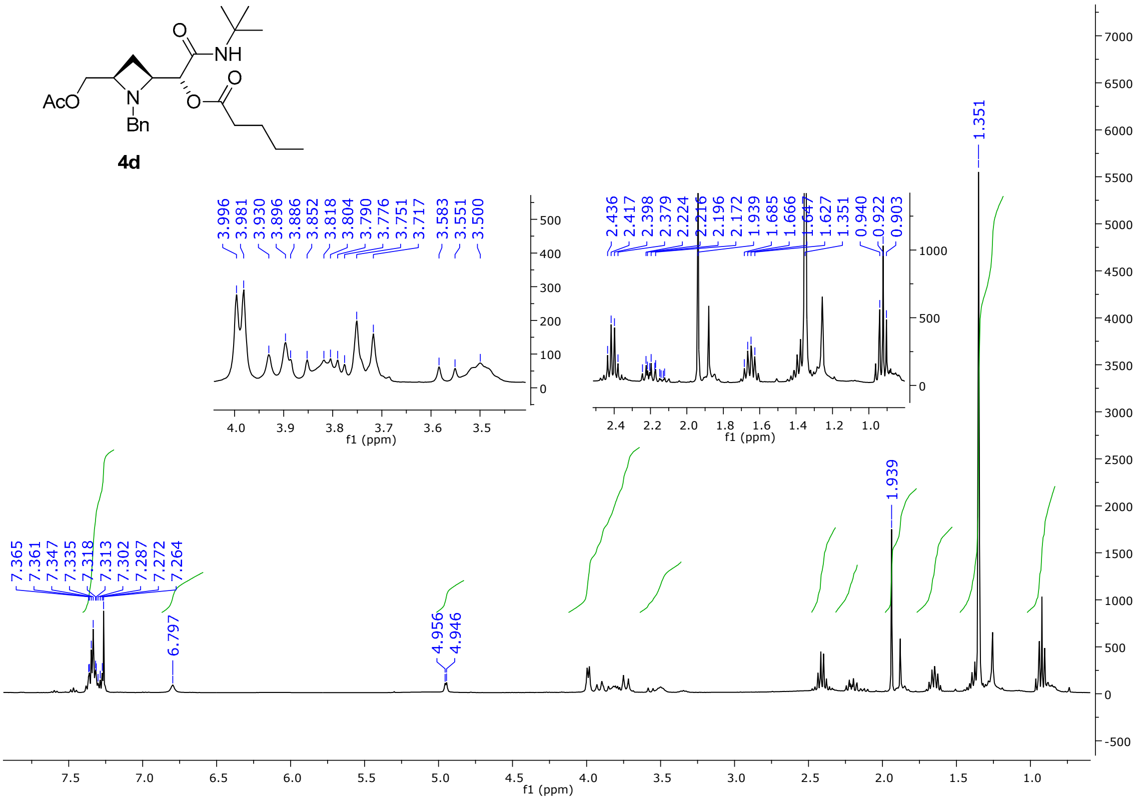

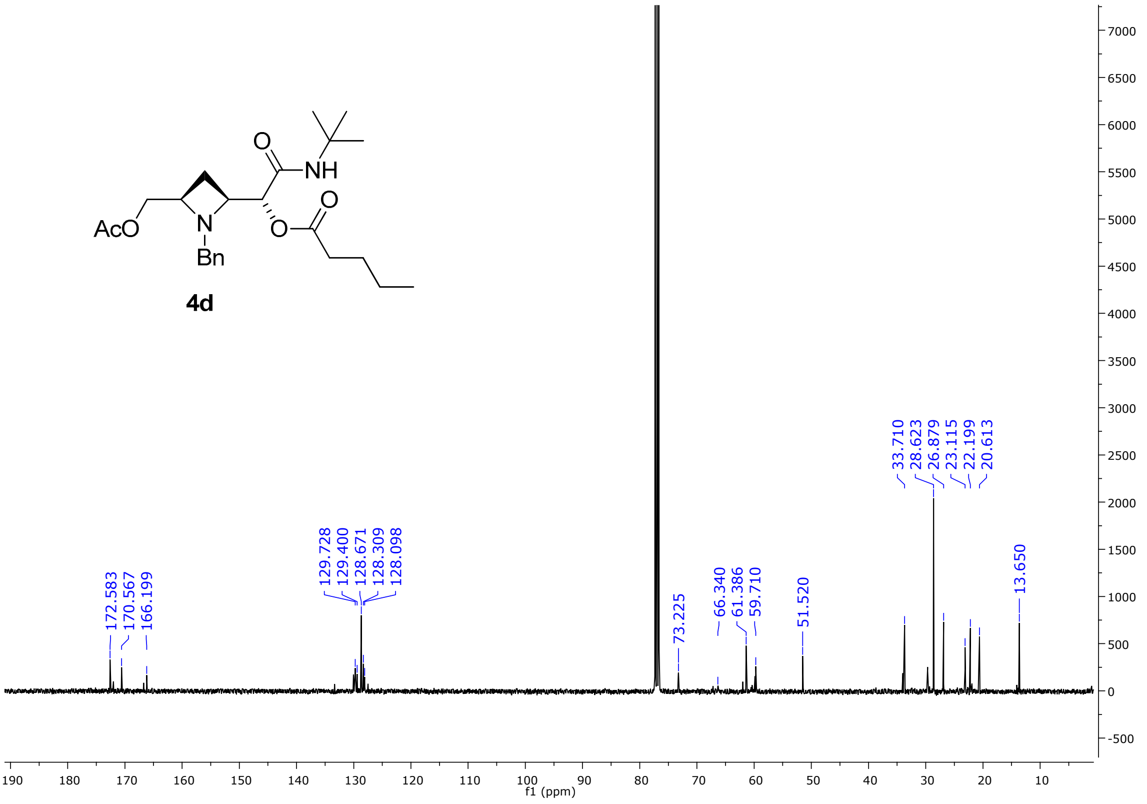

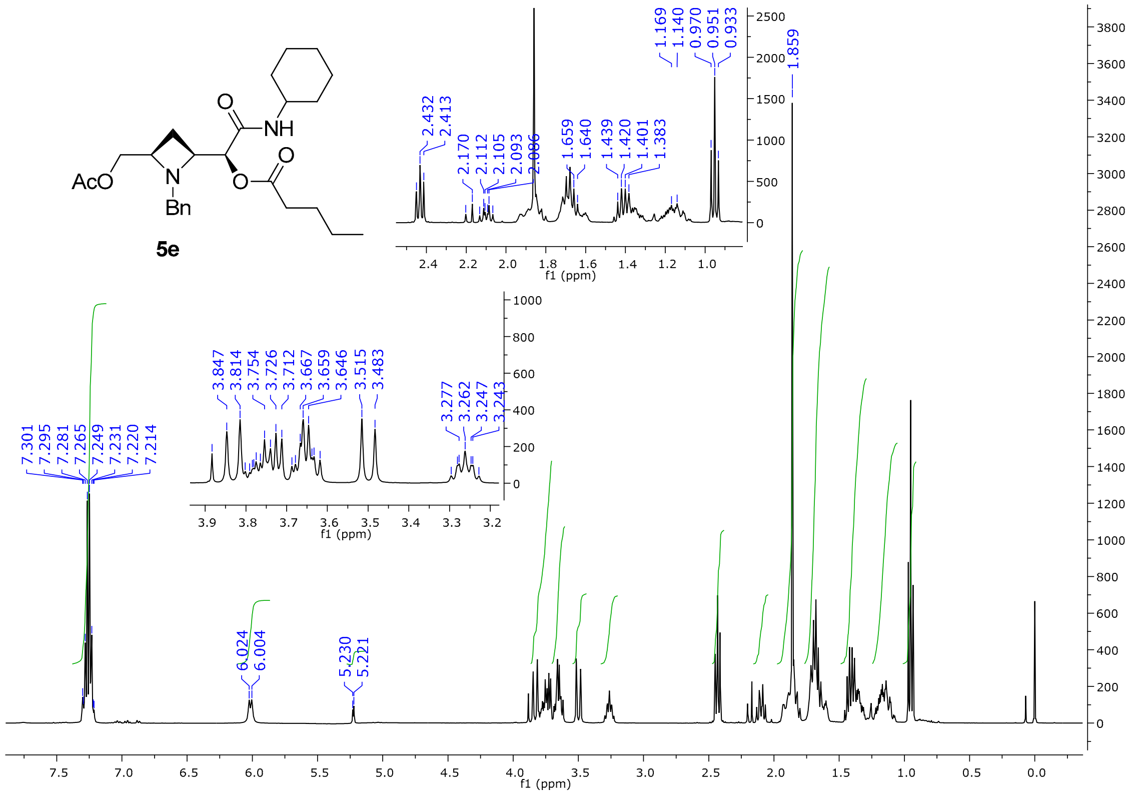
AcO
N
Bn
O
NH
O
O
5e
f1 (ppm)

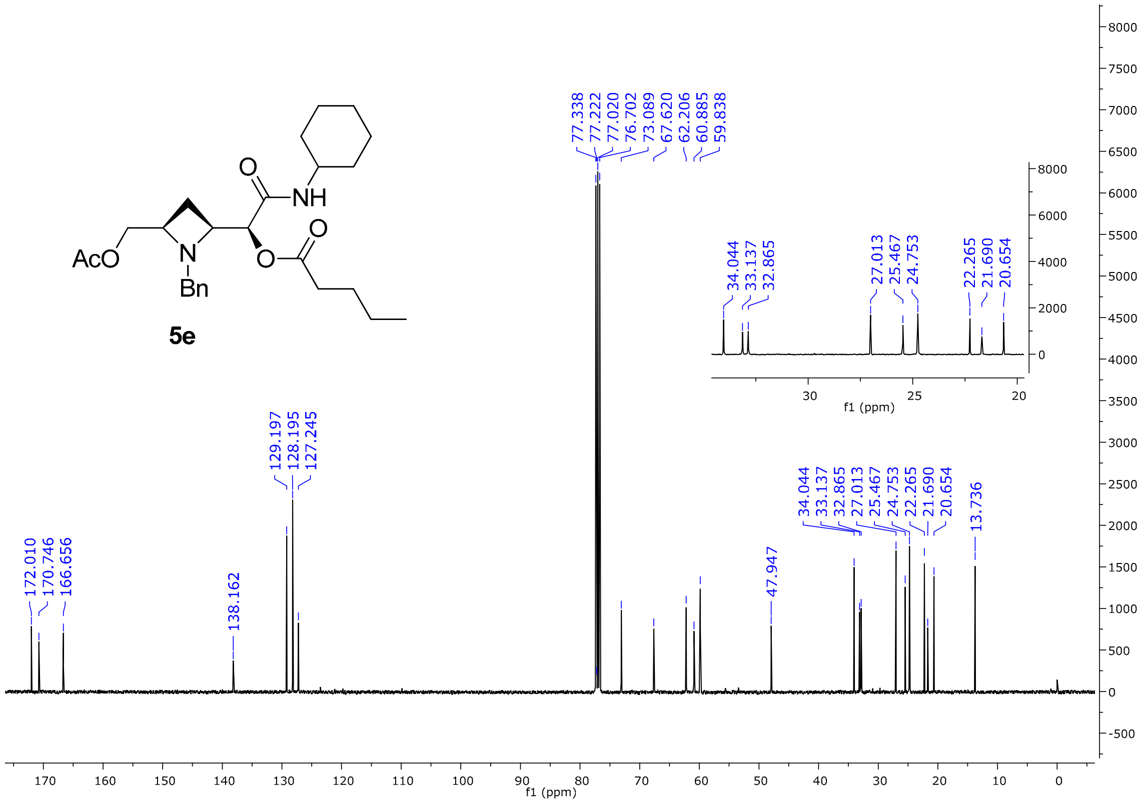
AcO
N
Bn
O
NH
O
O
5e
f1 (ppm)

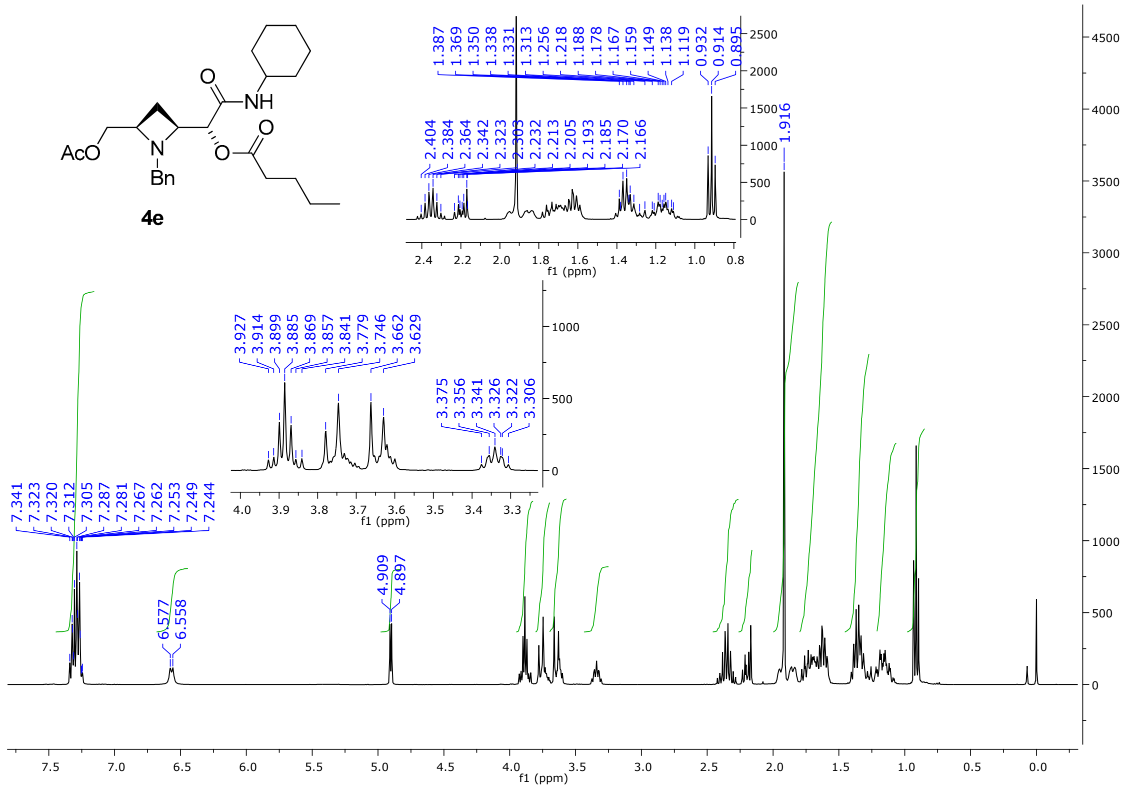
AcO
N
Bn
O
NH
O
O
4e
7.341
7.323
7.320
7.312
7.305
7.287
7.281
7.267
7.262
7.253
7.249
7.244
6.577
6.558
4.909
4.897
1.916
f1 (ppm)

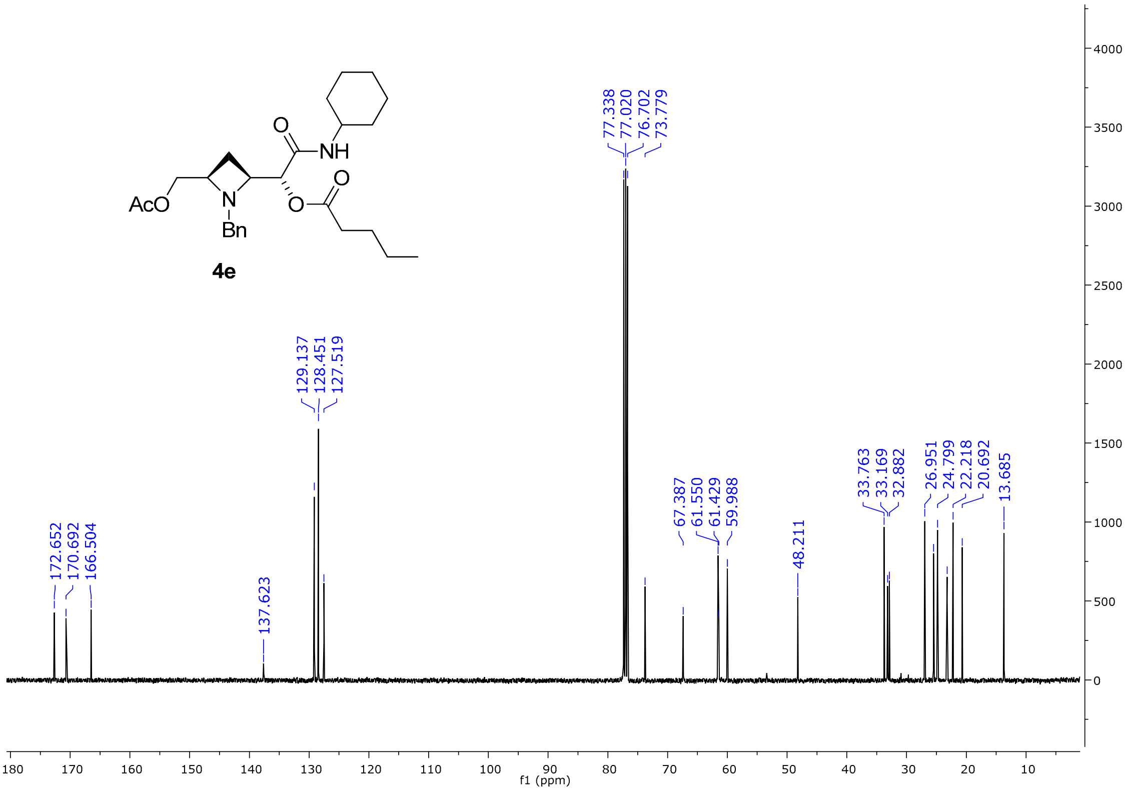
AcO
N
Bn
O
NH
O
O
4e
172.652
170.692
166.504
137.623
129.137
128.451
127.519
77.338
77.020
76.702
73.779
67.387
61.550
61.429
59.988
48.211
33.763
33.169
32.882
26.951
24.799
22.218
20.692
13.685
f1 (ppm)

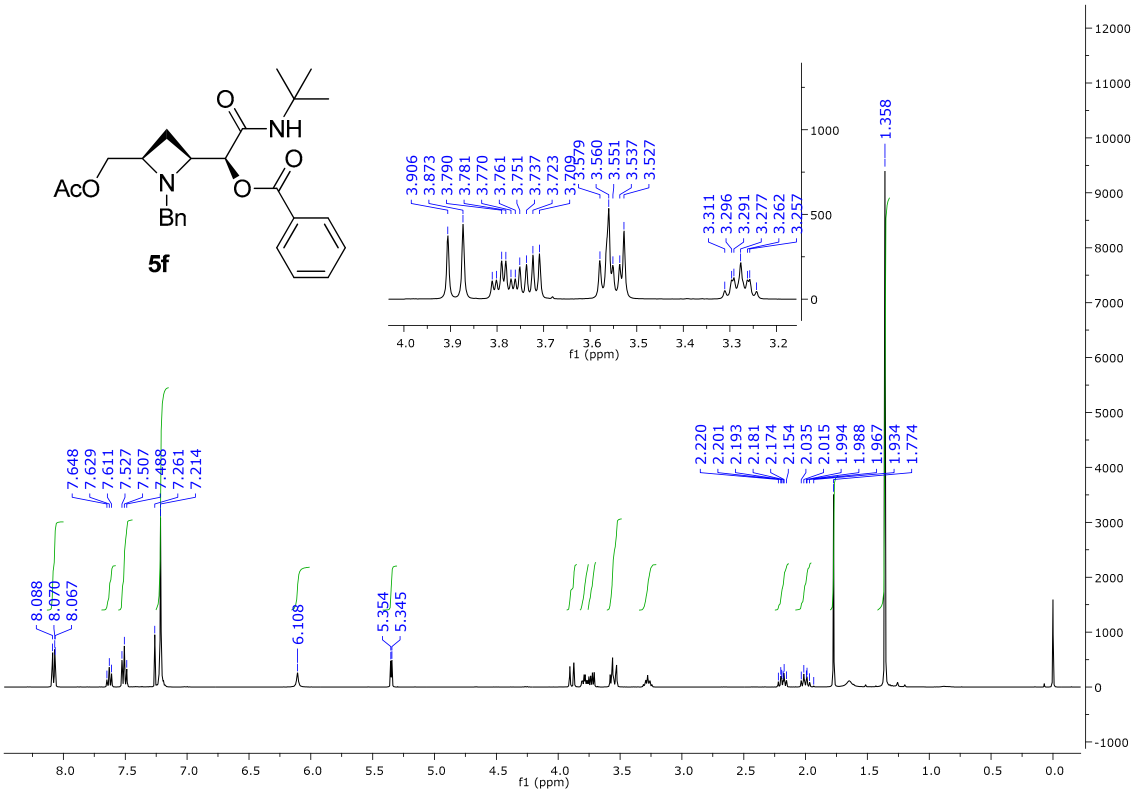

AcO
N
Bn
O
NH
O
O
5f
3.906
3.873
3.790
3.781
3.770
3.761
3.751
3.737
3.723
3.709
3.579
3.560
3.551
3.537
3.527
3.311
3.296
3.291
3.277
3.262
3.257
1.358
7.648
7.629
7.611
7.527
7.507
7.488
7.261
7.214
8.088
8.070
8.067
6.108
5.354
5.345
2.220
2.201
2.193
2.181
2.174
2.154
2.035
2.015
1.994
1.988
1.967
1.934
1.774
f1 (ppm)

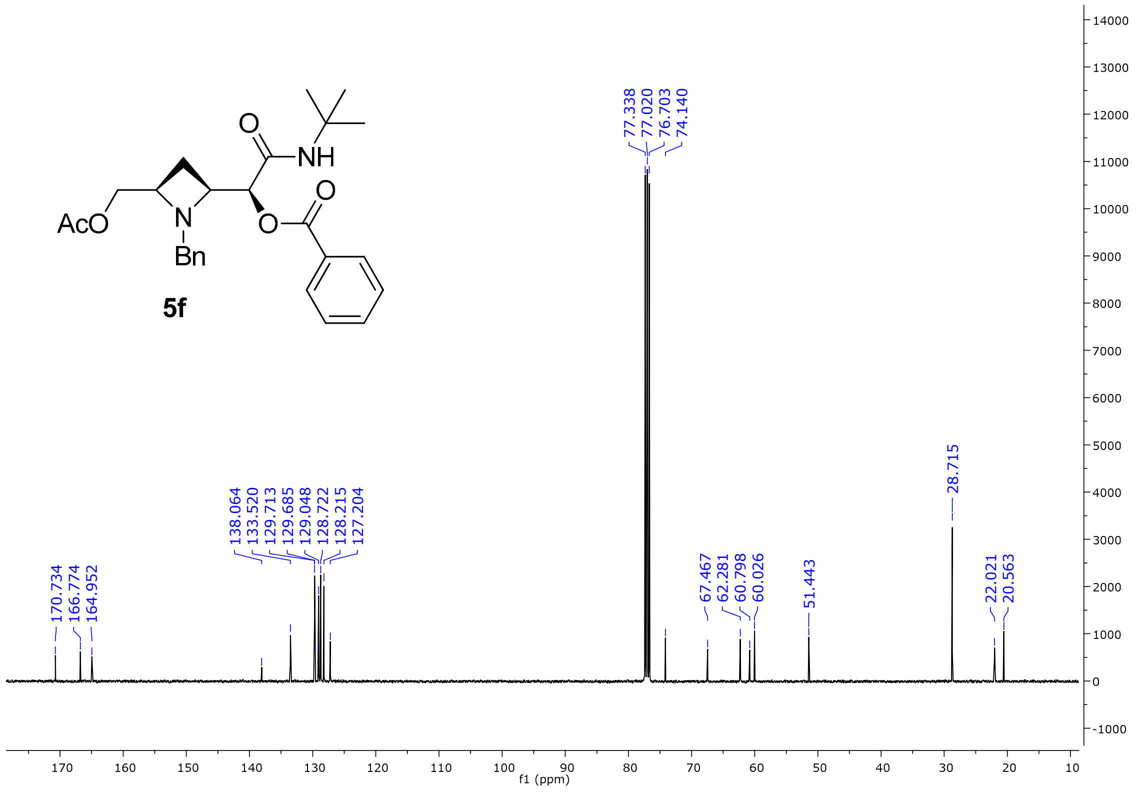

AcO
N
Bn
O
NH
O
O
5f
77.338
77.020
76.703
74.140
138.064
133.520
129.713
129.685
129.048
128.722
128.215
127.204
170.734
166.774
164.952
67.467
62.281
60.798
60.026
51.443
28.715
22.021
20.563
f1 (ppm)

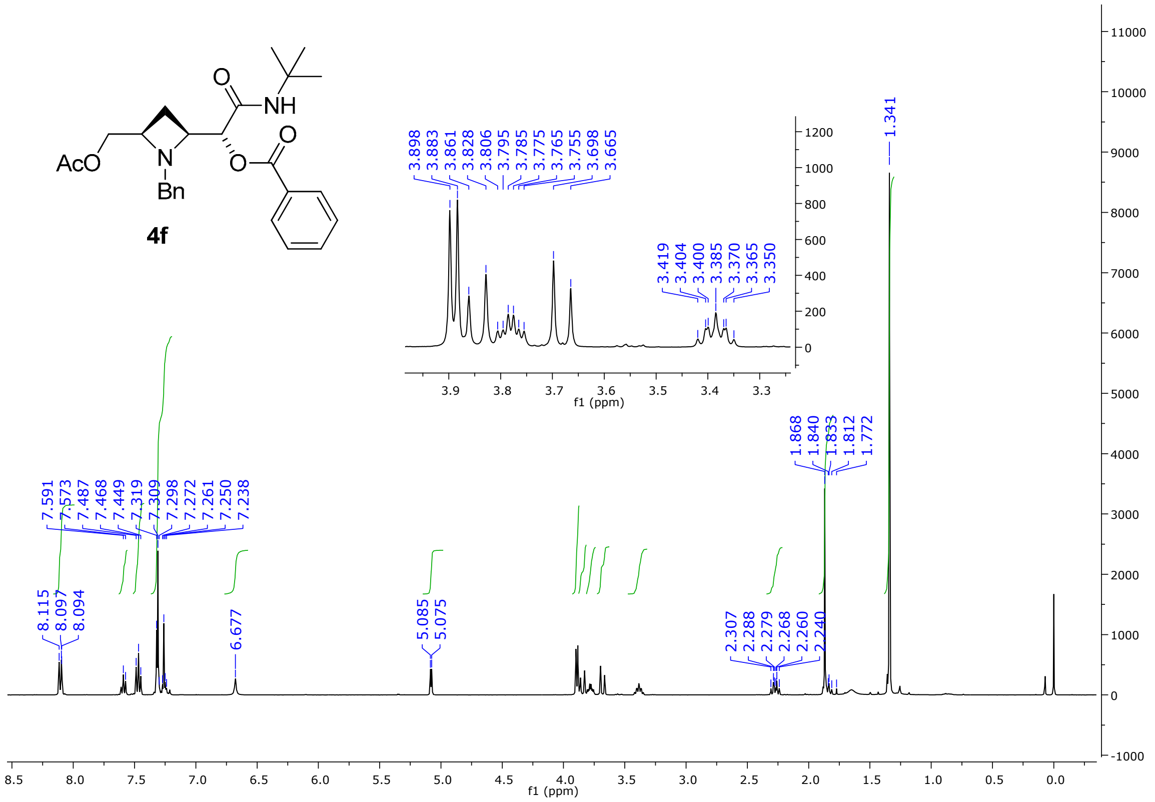

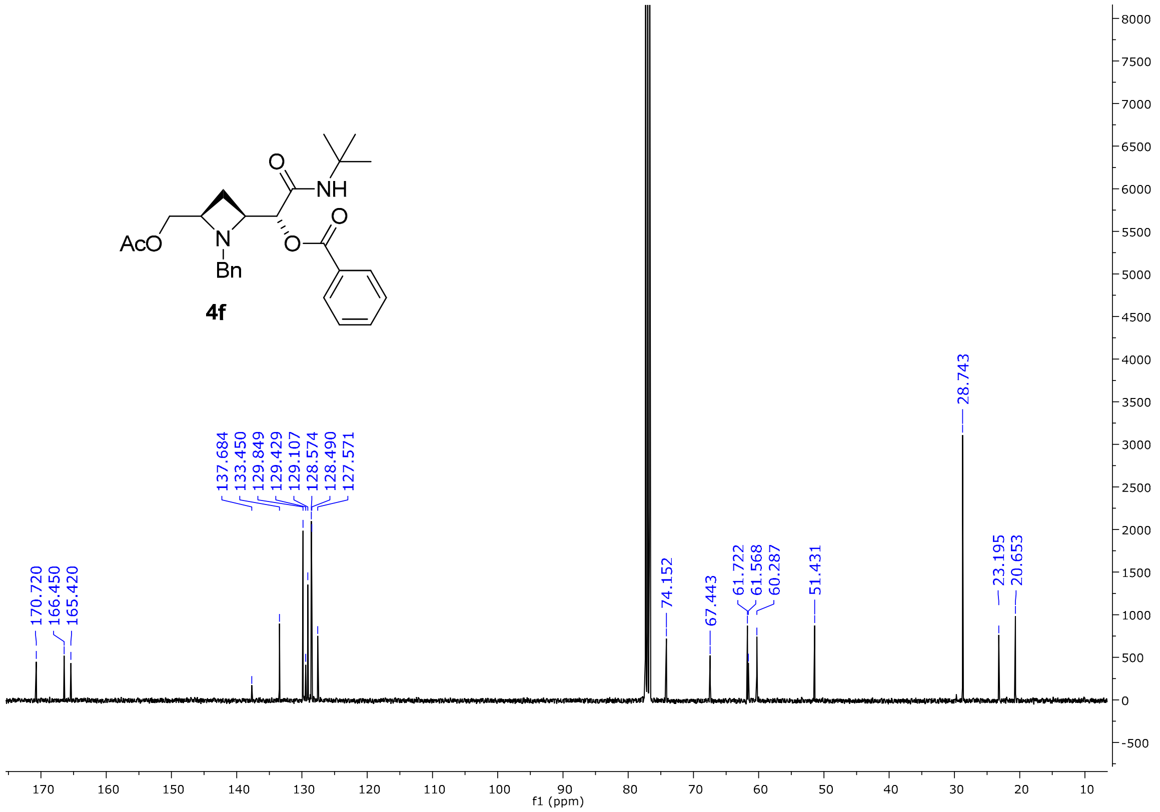

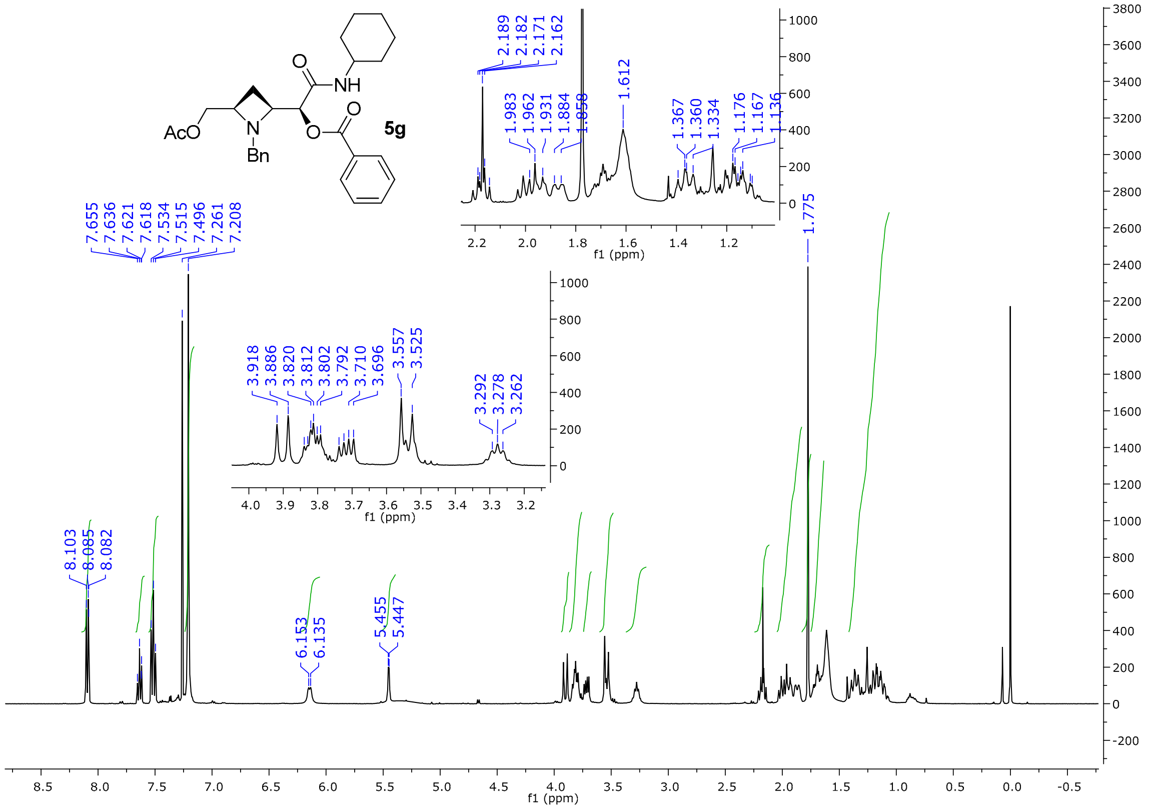

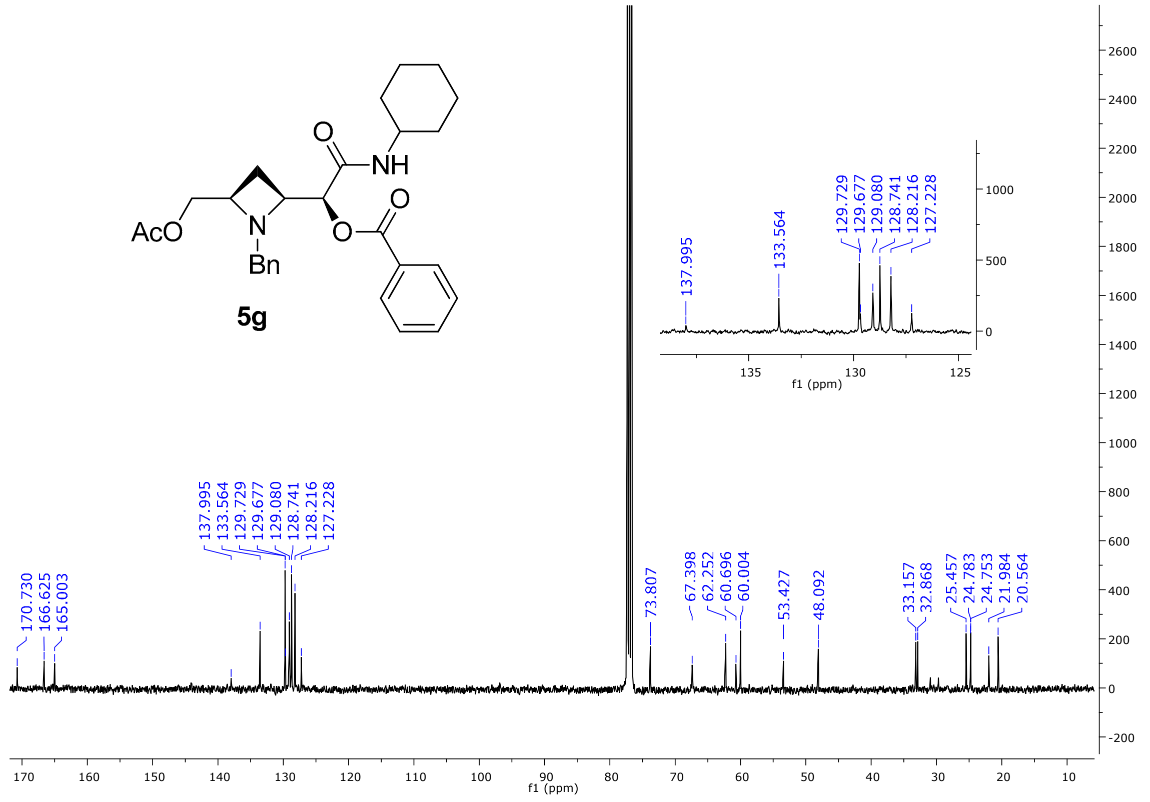

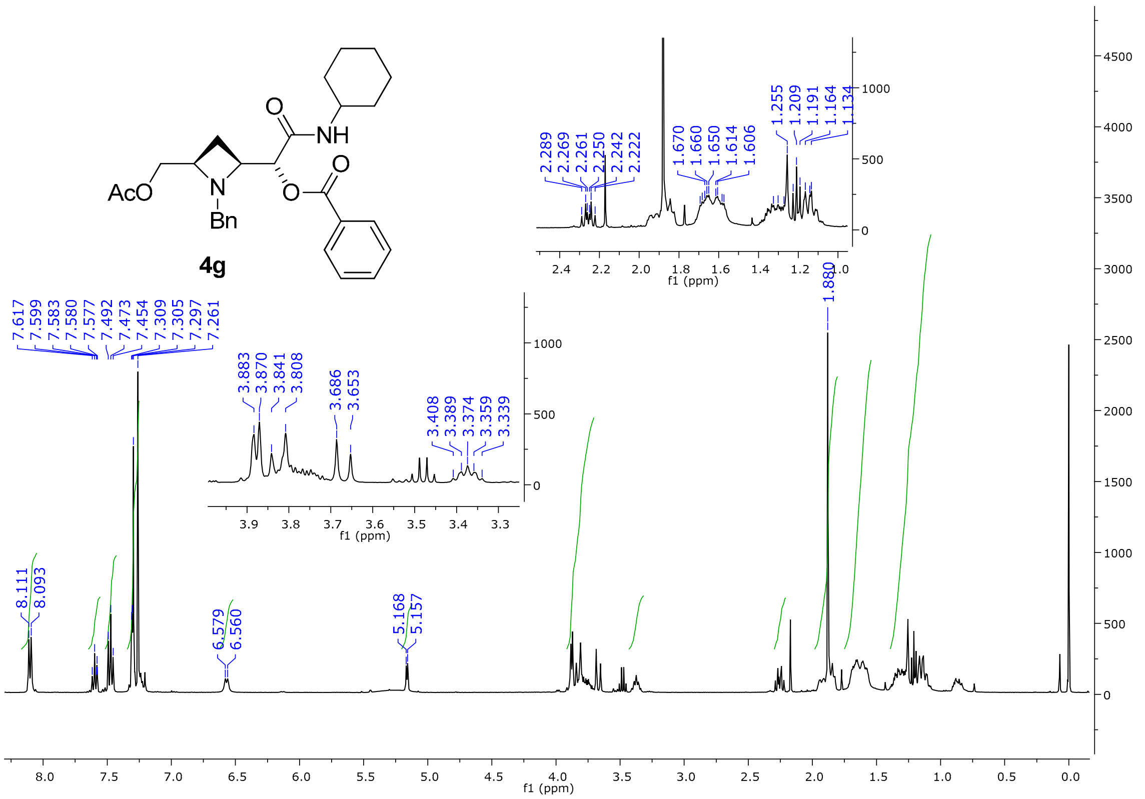
4g
AcO
N
Bn
O
NH
O
O
f1 (ppm)

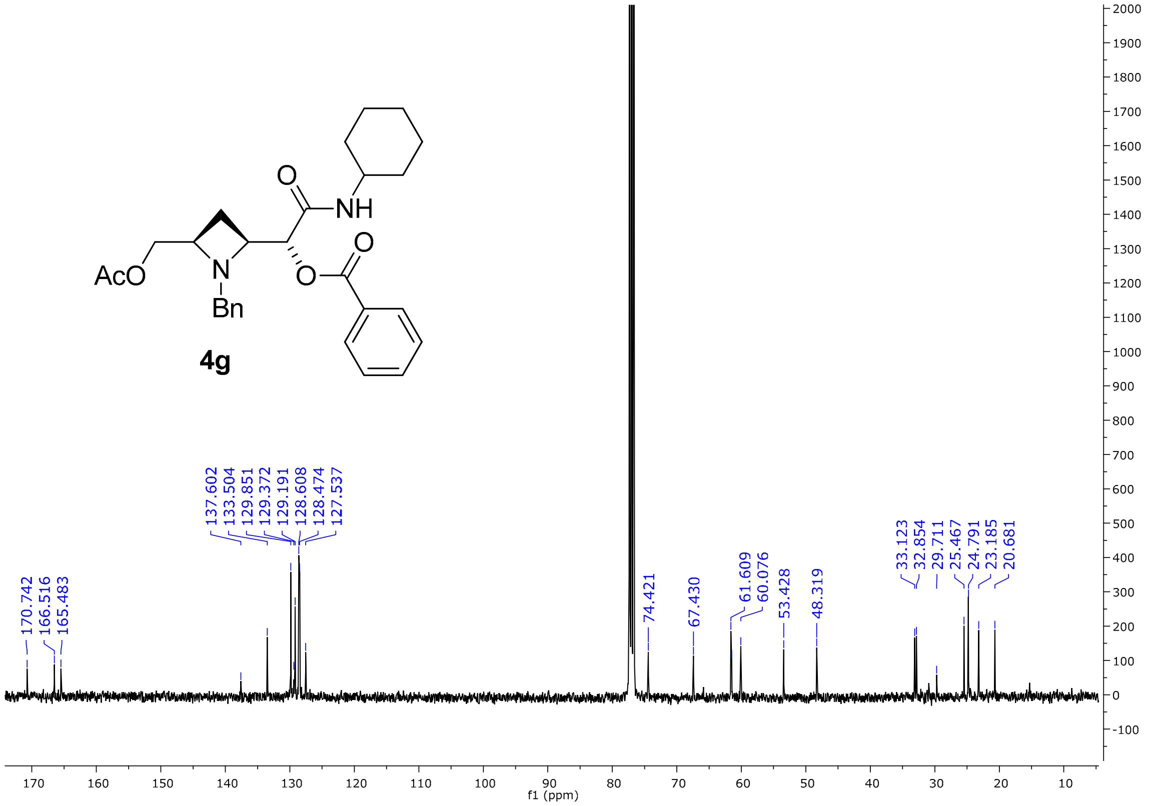
4g
AcO
N
Bn
O
NH
O
O
f1 (ppm)

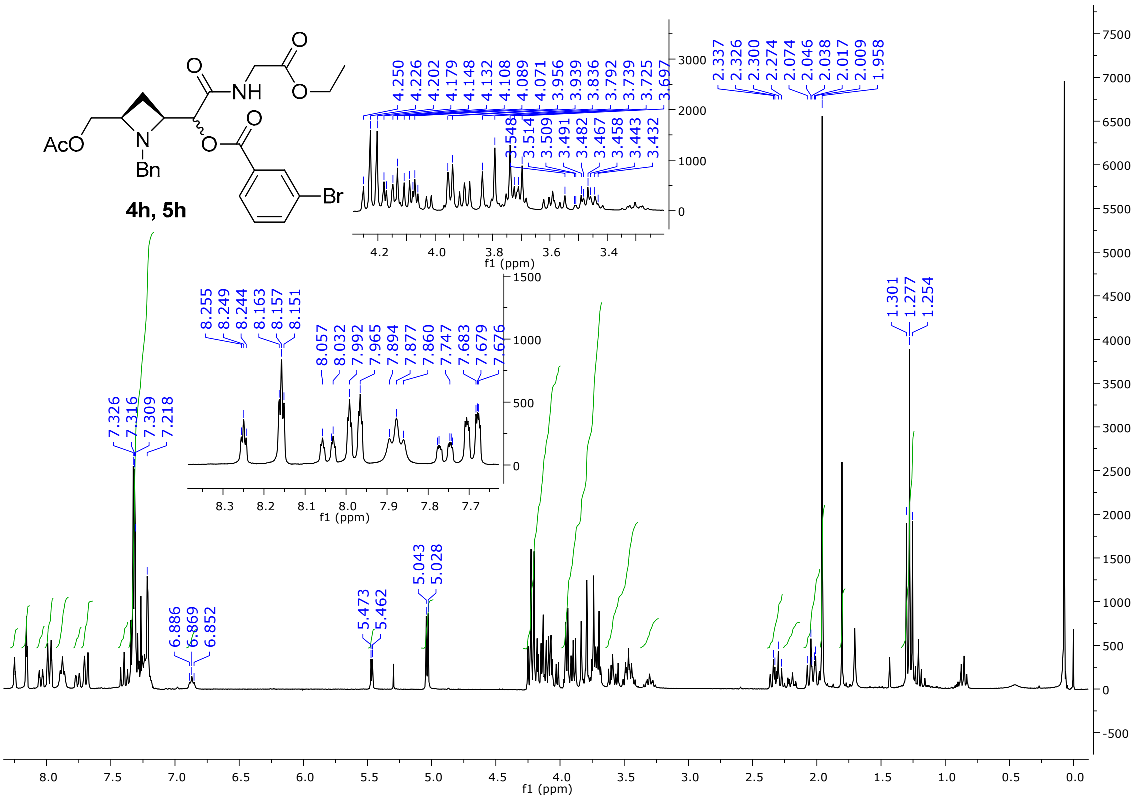
AcO
N
Bn
NH
O
Br
4h, 5h
f1 (ppm)

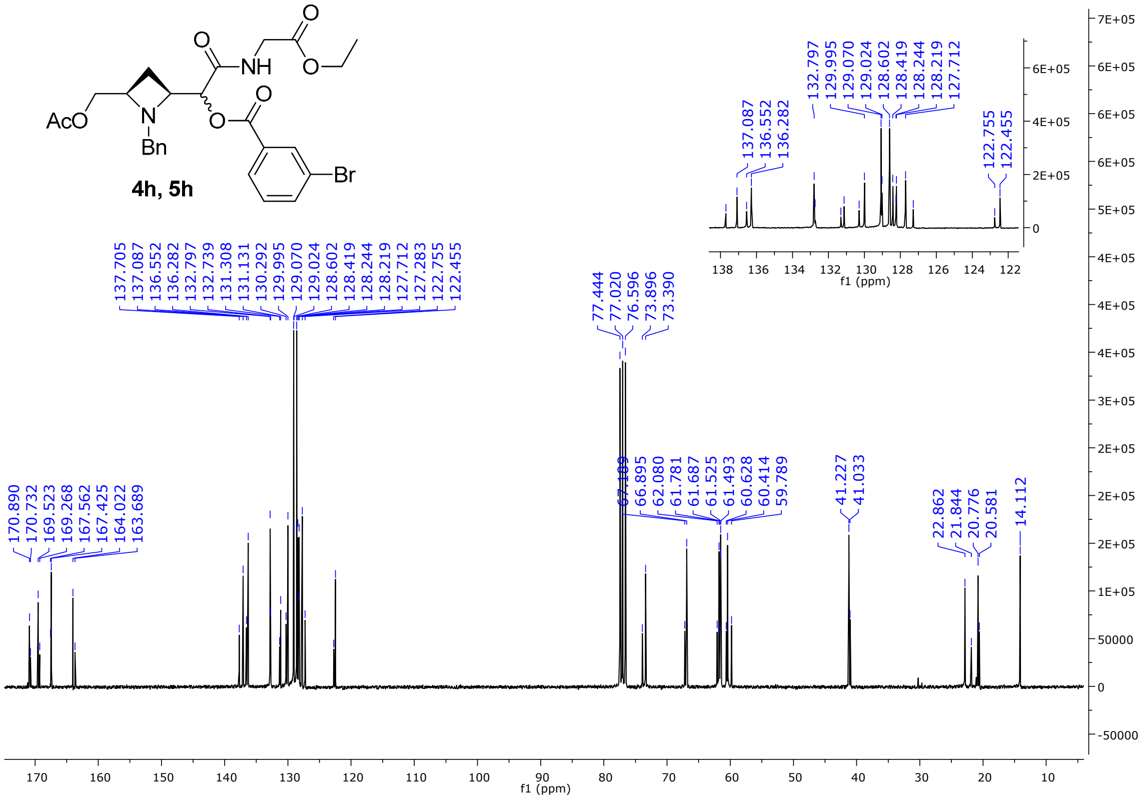
AcO
N
Bn
NH
O
Br
4h, 5h
f1 (ppm)

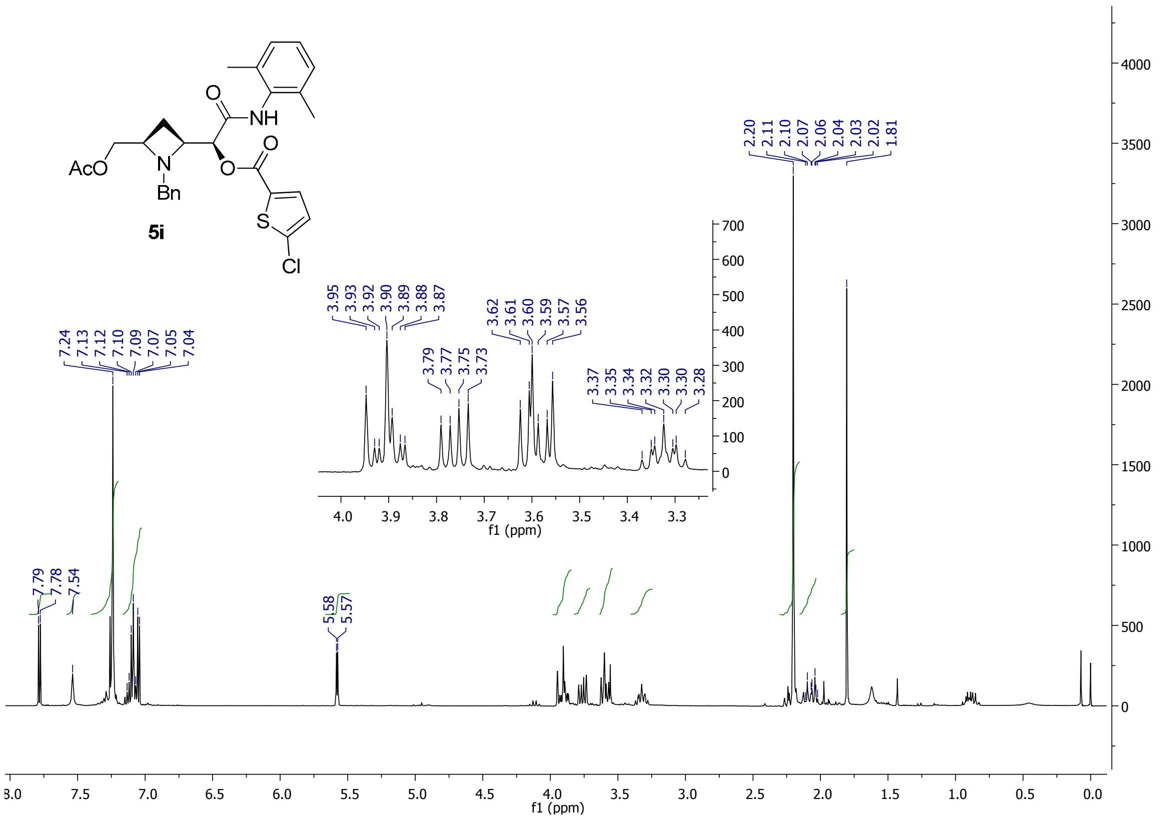
AcO
N
Bn
NH
O
S
Cl
5i
7.79
7.78
7.54
7.24
7.13
7.12
7.10
7.09
7.07
7.05
7.04
5.58
5.57
3.95
3.93
3.92
3.90
3.89
3.88
3.87
3.79
3.77
3.75
3.73
3.62
3.61
3.60
3.59
3.57
3.56
3.37
3.35
3.34
3.32
3.30
3.30
3.28
2.20
2.11
2.10
2.07
2.06
2.04
2.03
2.02
1.81
f1 (ppm)

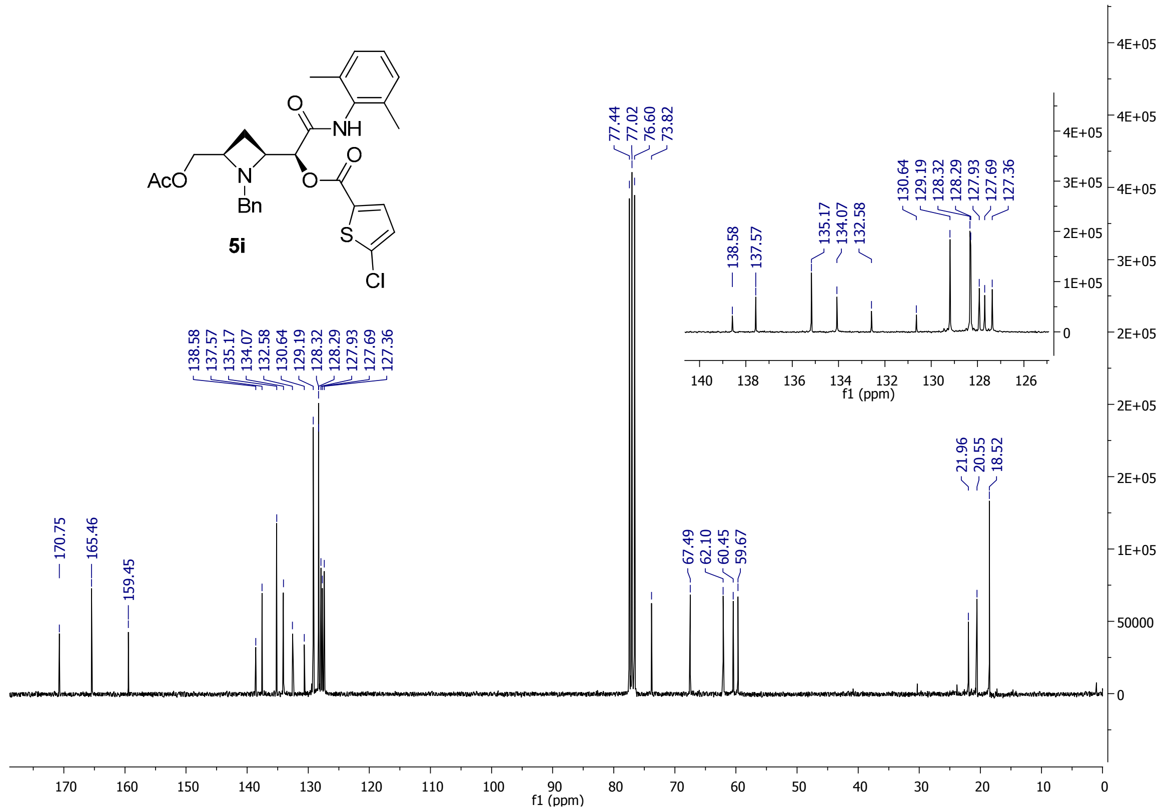
AcO
N
Bn
NH
O
S
Cl
5i
170.75
165.46
159.45
138.58
137.57
135.17
134.07
132.58
130.64
129.19
128.32
128.29
127.93
127.69
127.36
77.44
77.02
76.60
73.82
67.49
62.10
60.45
59.67
21.96
20.55
18.52
f1 (ppm)

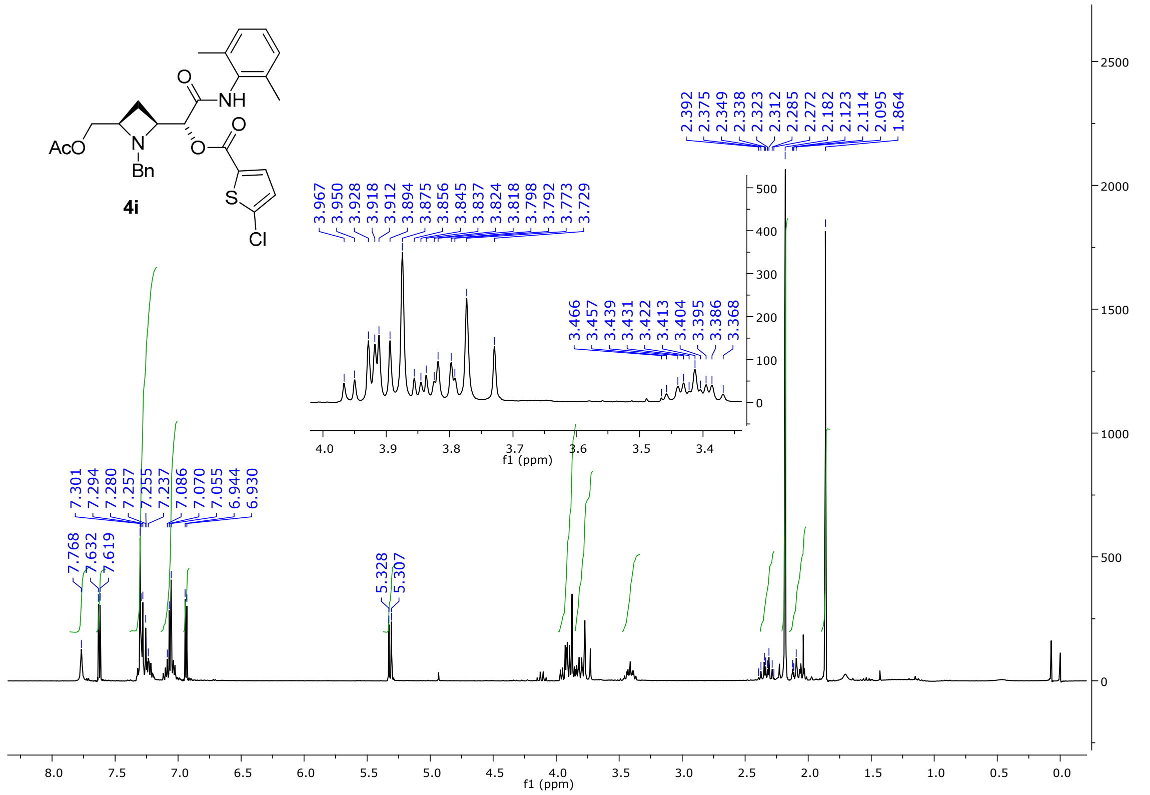

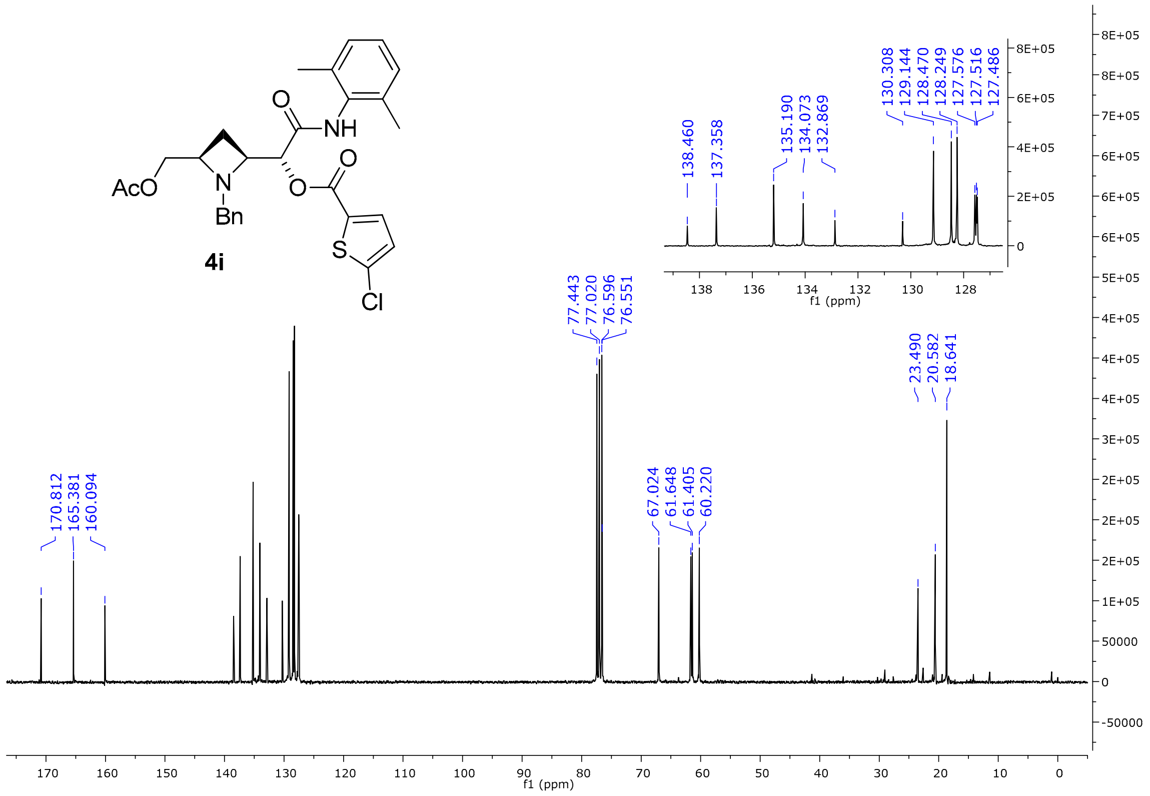

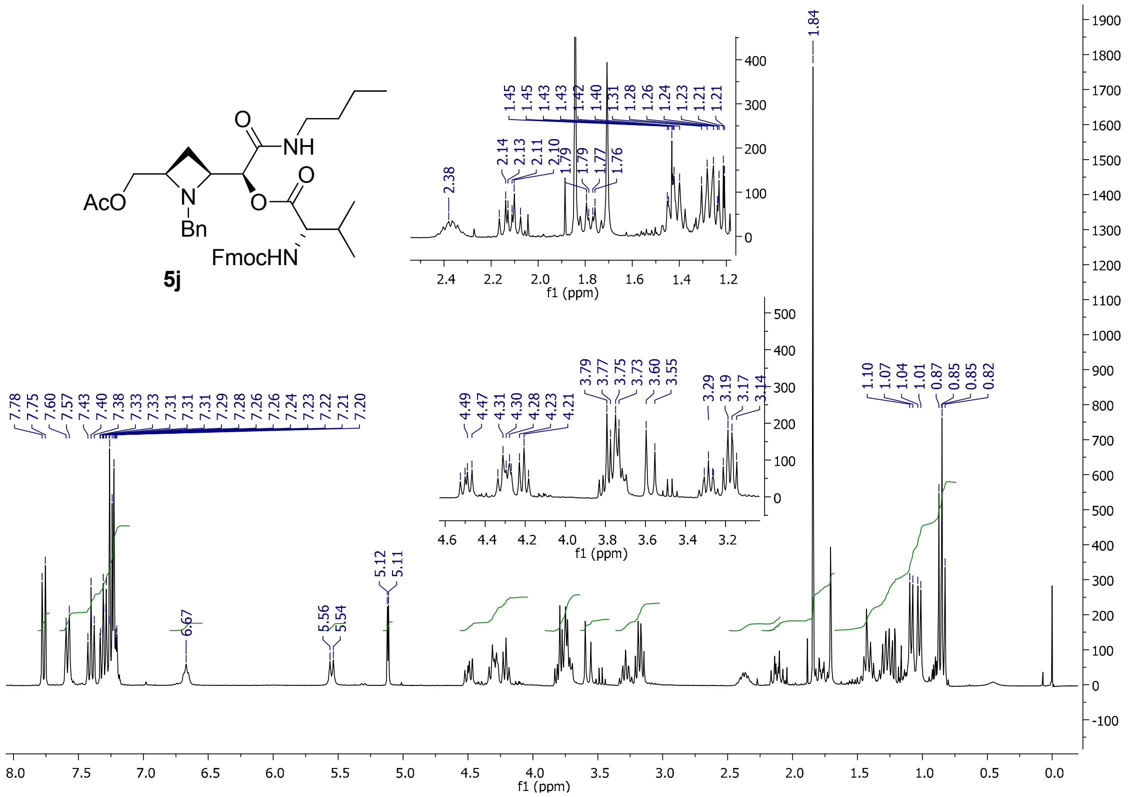
AcO
N
Bn
O
NH
O
O
FmocHN
5j
7.78 7.75 7.60 7.57 7.43 7.40 7.38 7.33 7.33 7.31 7.31 7.31 7.29 7.28 7.26 7.26 7.24 7.23 7.22 7.21 7.20
6.67
5.56 5.54
5.12 5.11
4.49 4.47 4.31 4.30 4.28 4.23 4.21
3.79 3.77 3.75 3.73 3.60 3.55
3.29 3.19 3.17 3.14
2.38
2.14 2.13 2.11 2.10 1.79 1.79 1.77 1.76
1.45 1.45 1.43 1.43 1.42 1.40 1.31 1.28 1.26 1.24 1.23 1.21 1.21 1.21
1.84
1.10 1.07 1.04 1.01 0.87 0.85 0.85 0.82
f1 (ppm)

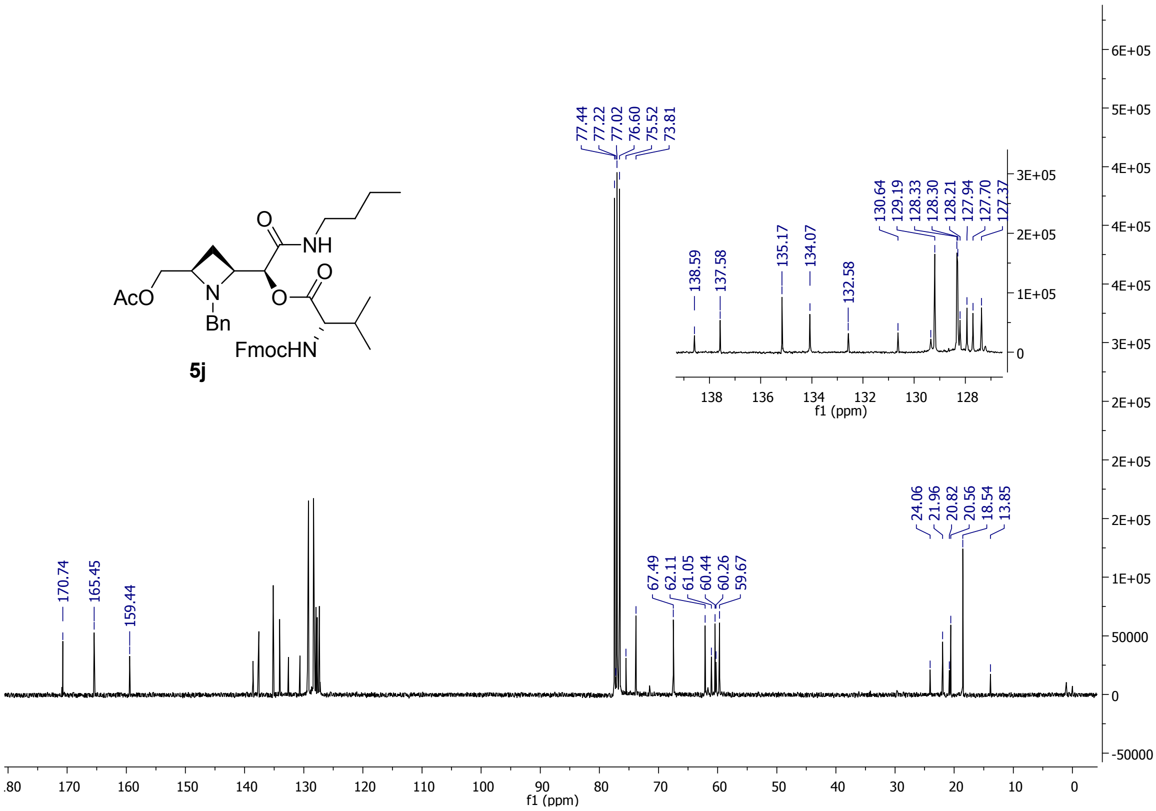
AcO
N
Bn
O
NH
O
O
FmocHN
5j
170.74
165.45
159.44
138.59
137.58
135.17
134.07
132.58
130.64 129.19 128.33 128.30 128.21 127.94 127.70 127.37
77.44 77.22 77.02 76.60 75.52 73.81
67.49 62.11 61.05 60.44 60.26 59.67
24.06 21.96 20.82 20.56 18.54 13.85
f1 (ppm)

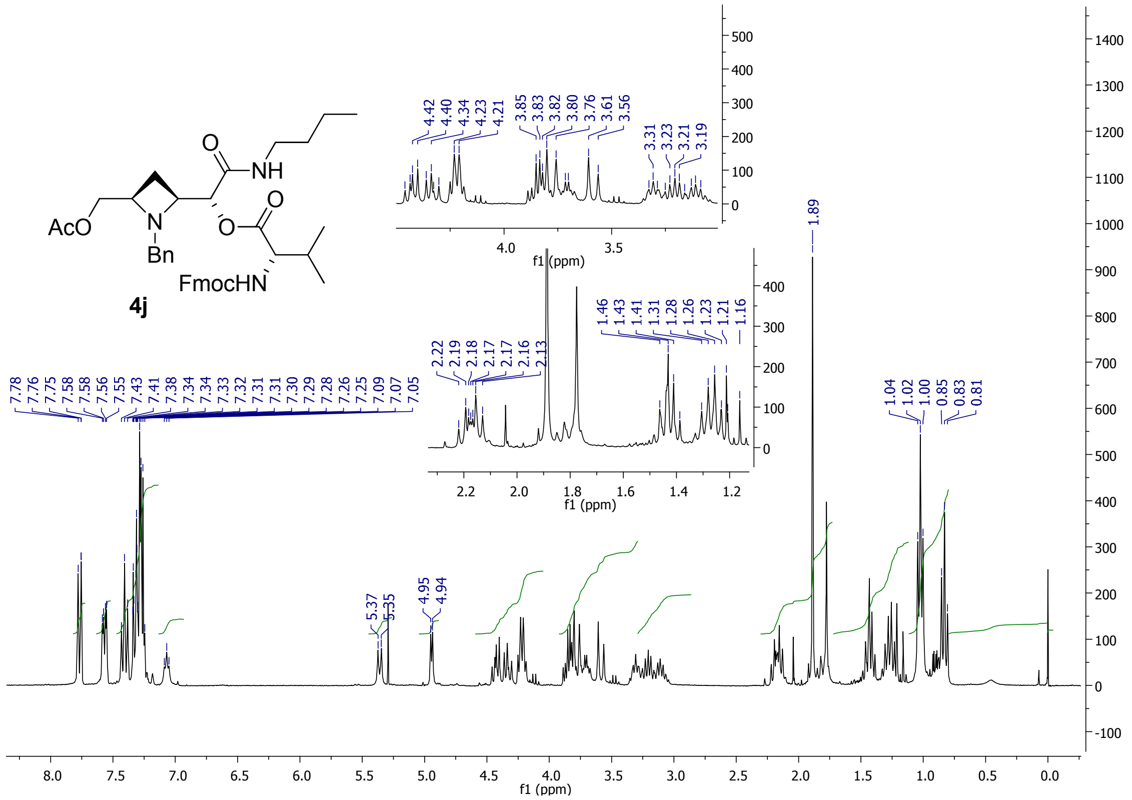

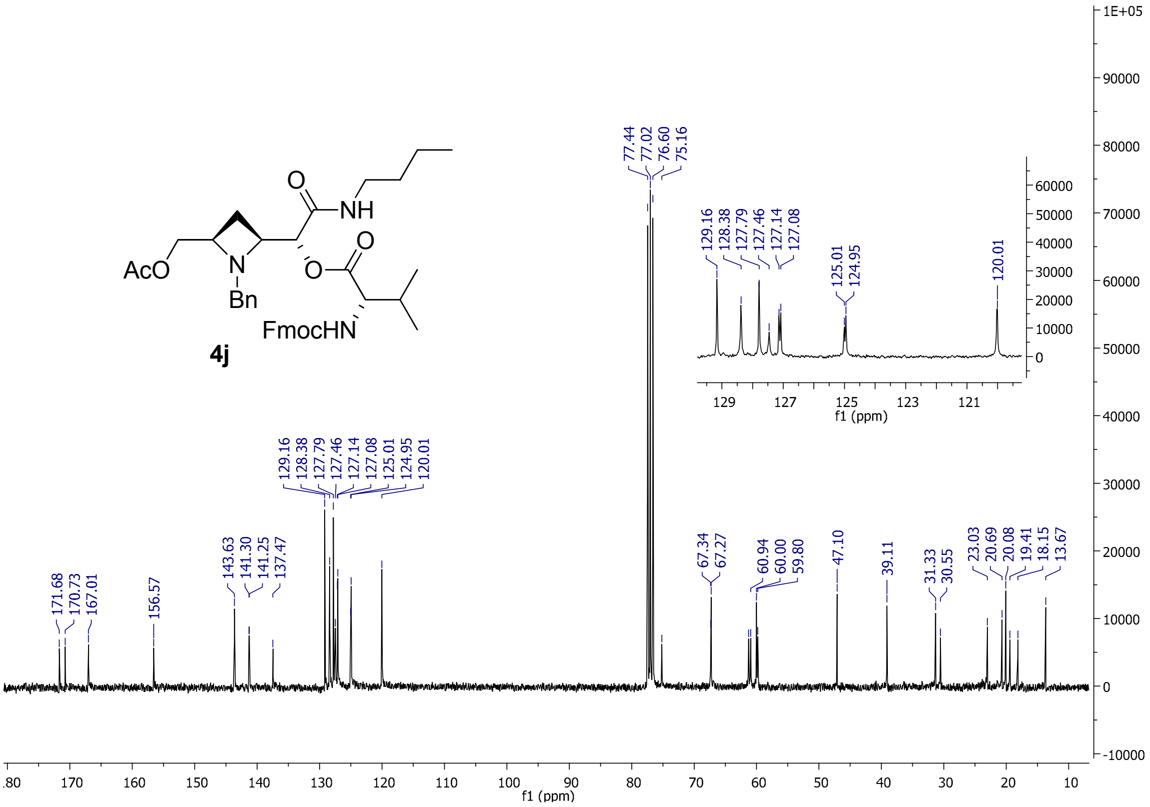

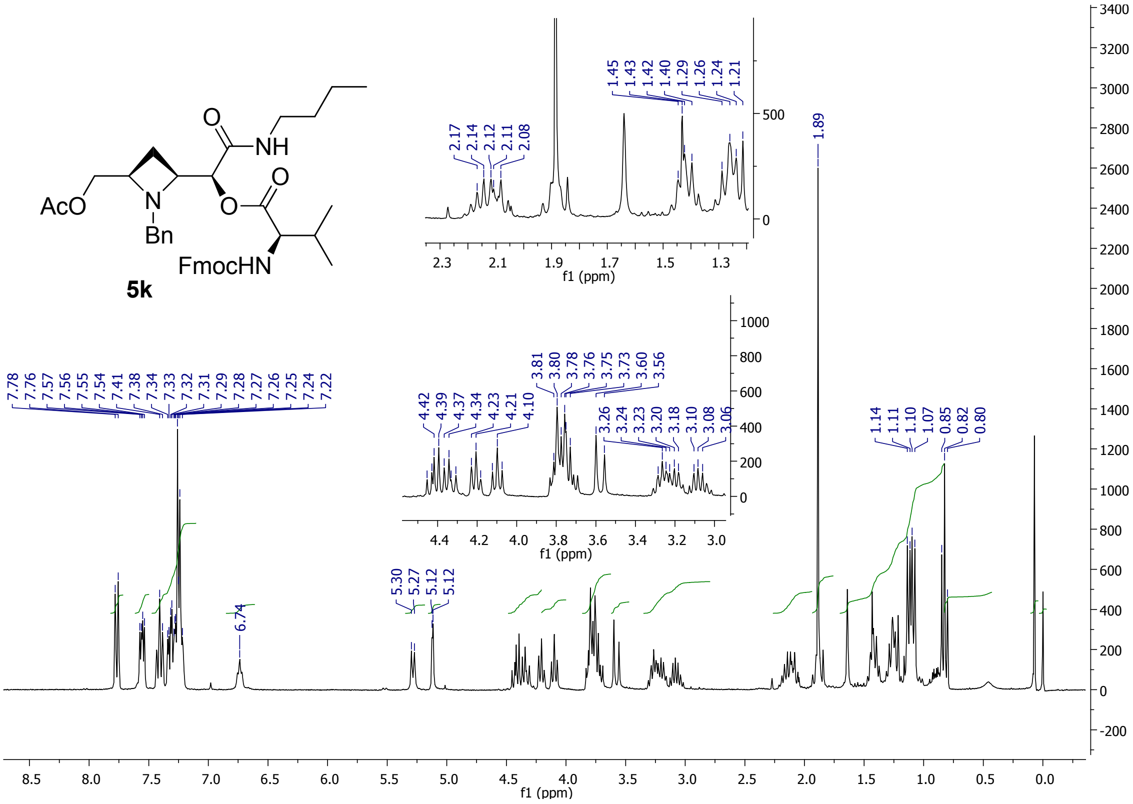

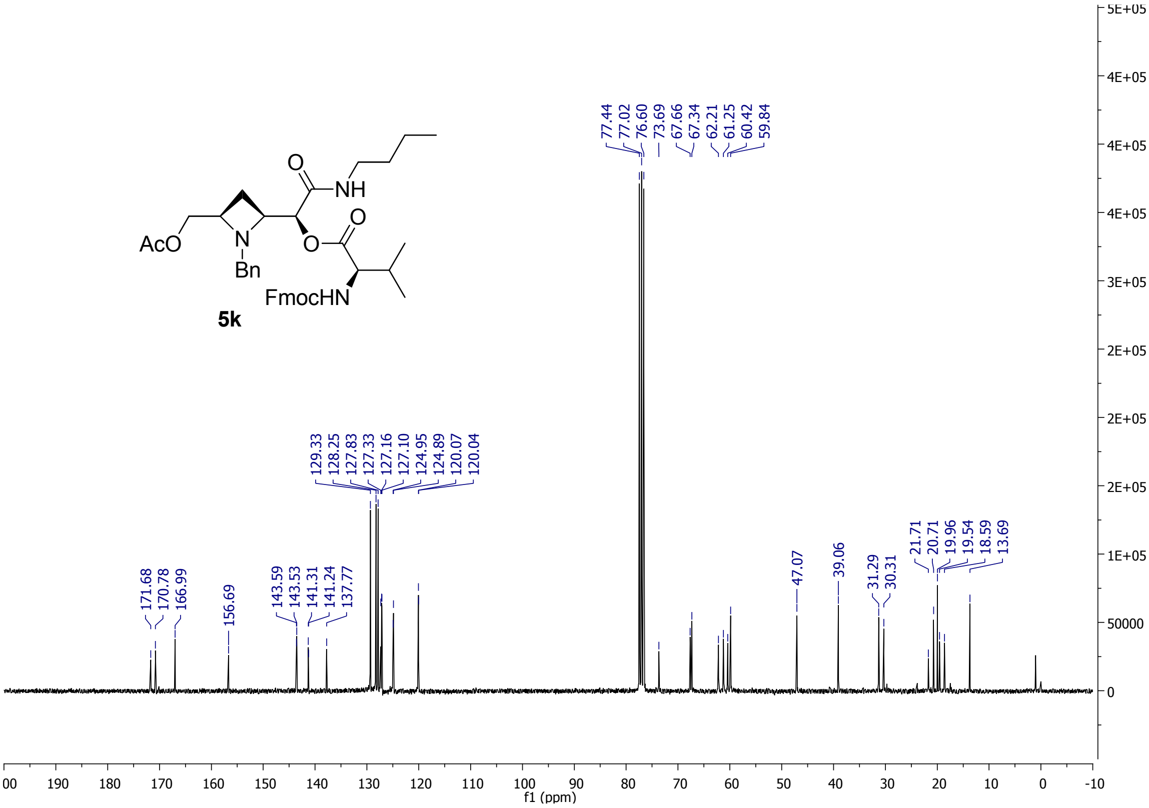

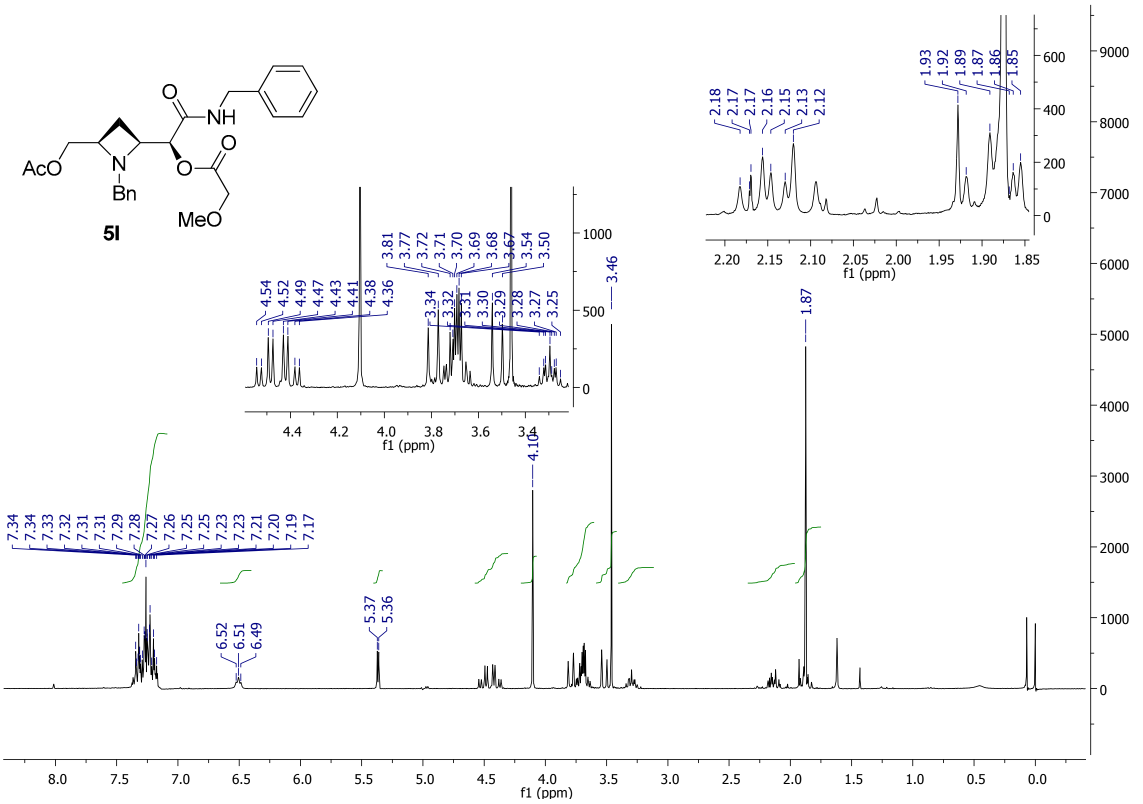
O
NH
O
AcO
N
O
Bn
MeO
5l
f1 (ppm)

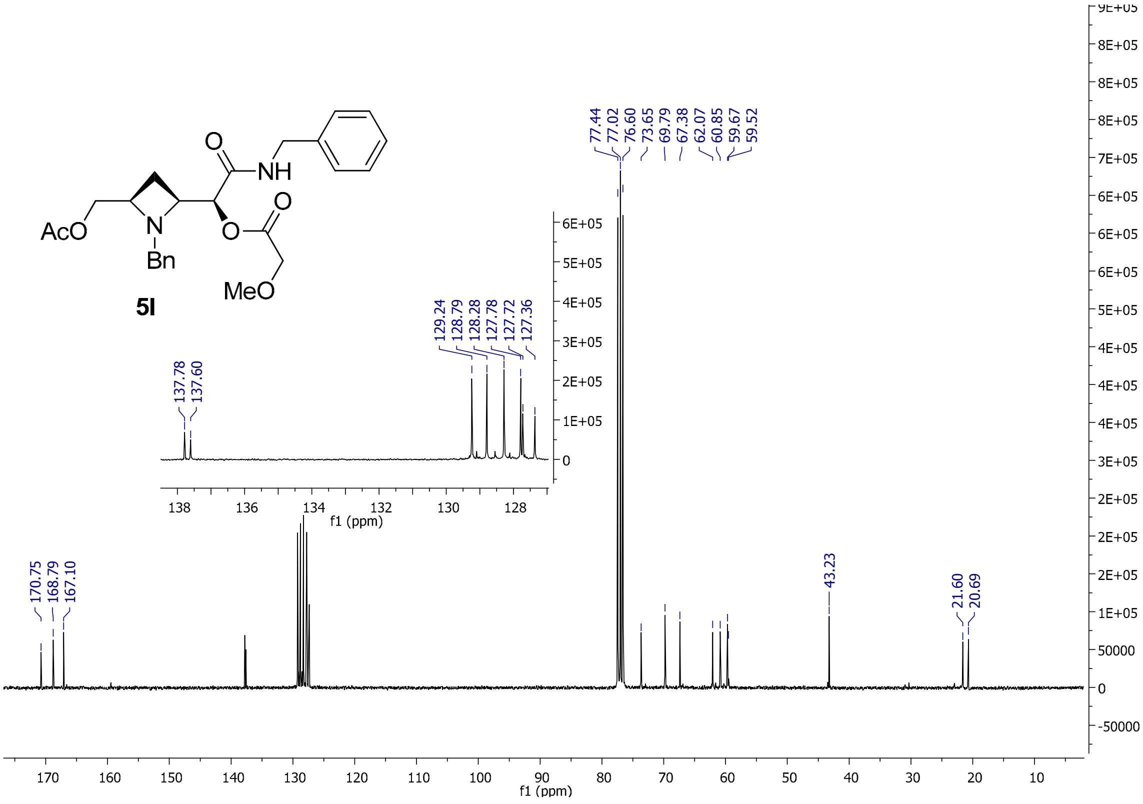
O
NH
O
AcO
N
O
Bn
MeO
5l
f1 (ppm)

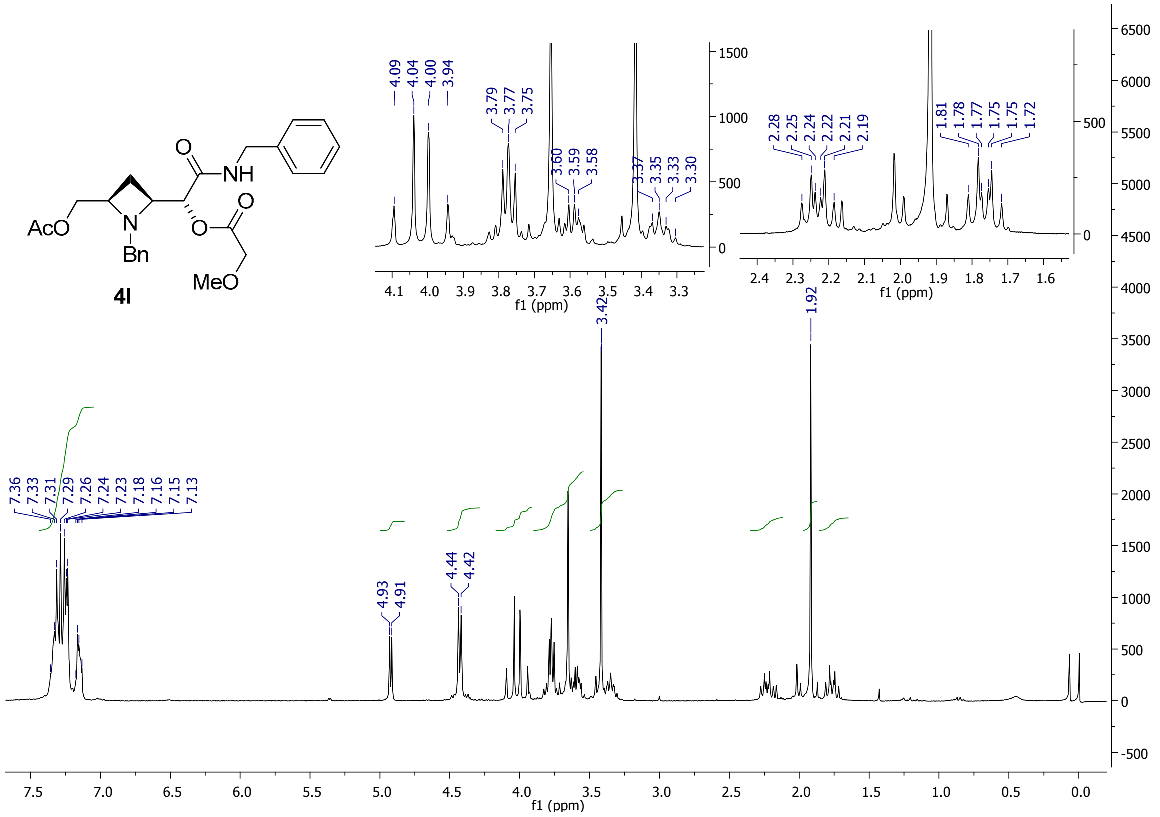
AcO
N
Bn
O
NH
O
O
MeO
4l
f1 (ppm)

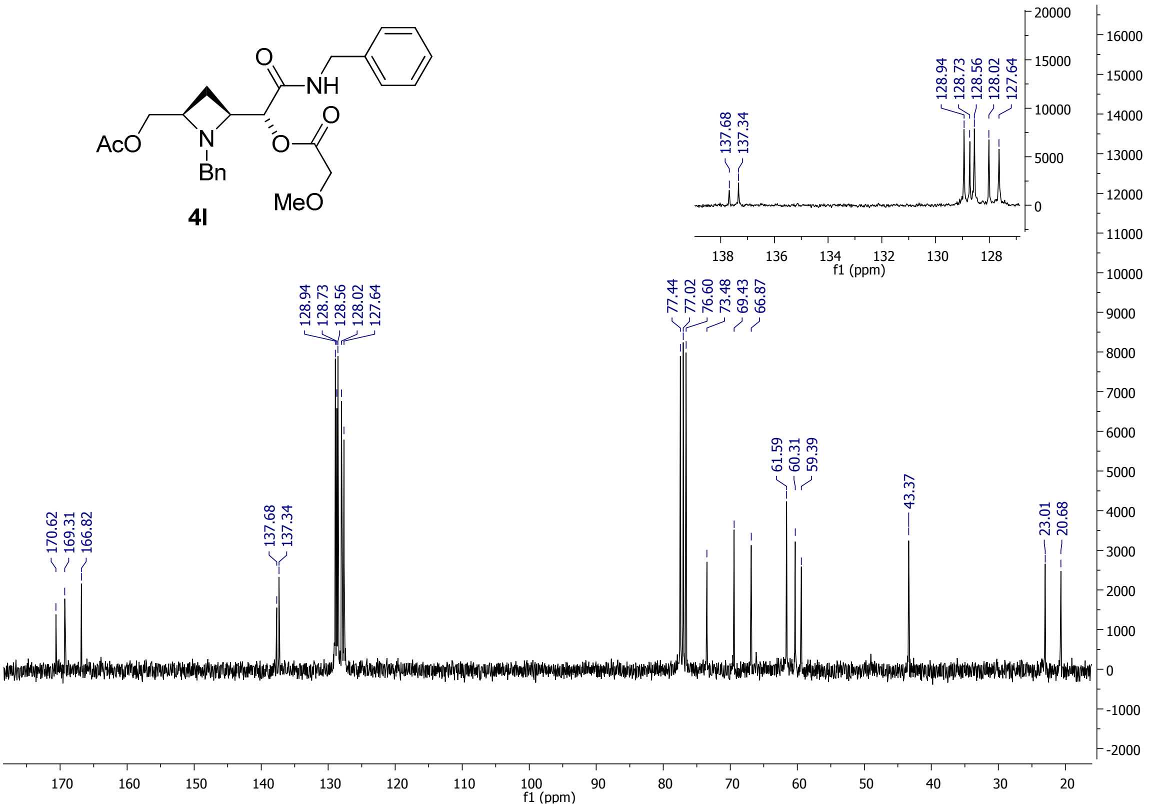
AcO
N
Bn
O
NH
O
O
MeO
4l
f1 (ppm)

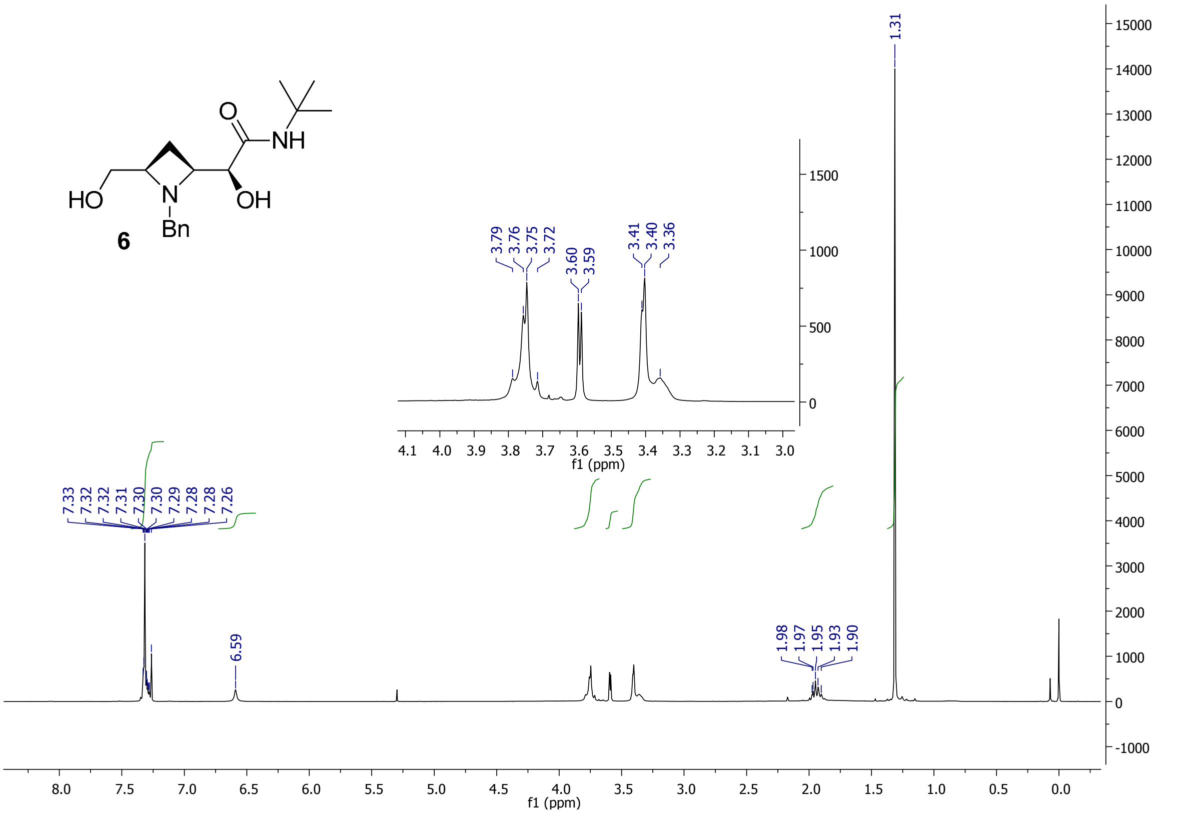
HO
OH
NH
O
N
Bn
6
7.33 7.32 7.32 7.31 7.30 7.30 7.29 7.28 7.28 7.26
6.59
3.79 3.76 3.75 3.72
3.60 3.59
3.41 3.40 3.36
1.98 1.97 1.95 1.93 1.90
1.31
f1 (ppm)

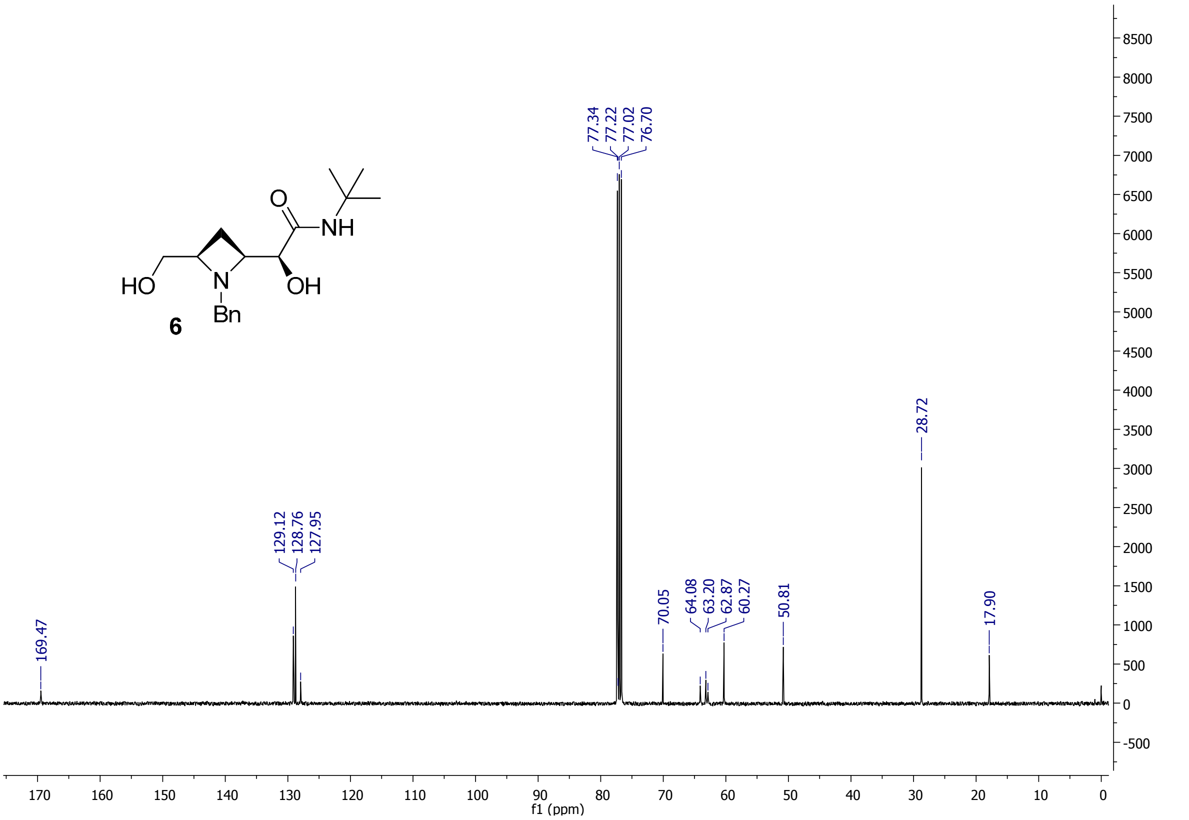
HO
OH
NH
O
N
Bn
6
169.47
129.12 128.76 127.95
77.34 77.22 77.02 76.70
70.05
64.08 63.20 62.87 60.27
50.81
28.72
17.90
f1 (ppm)

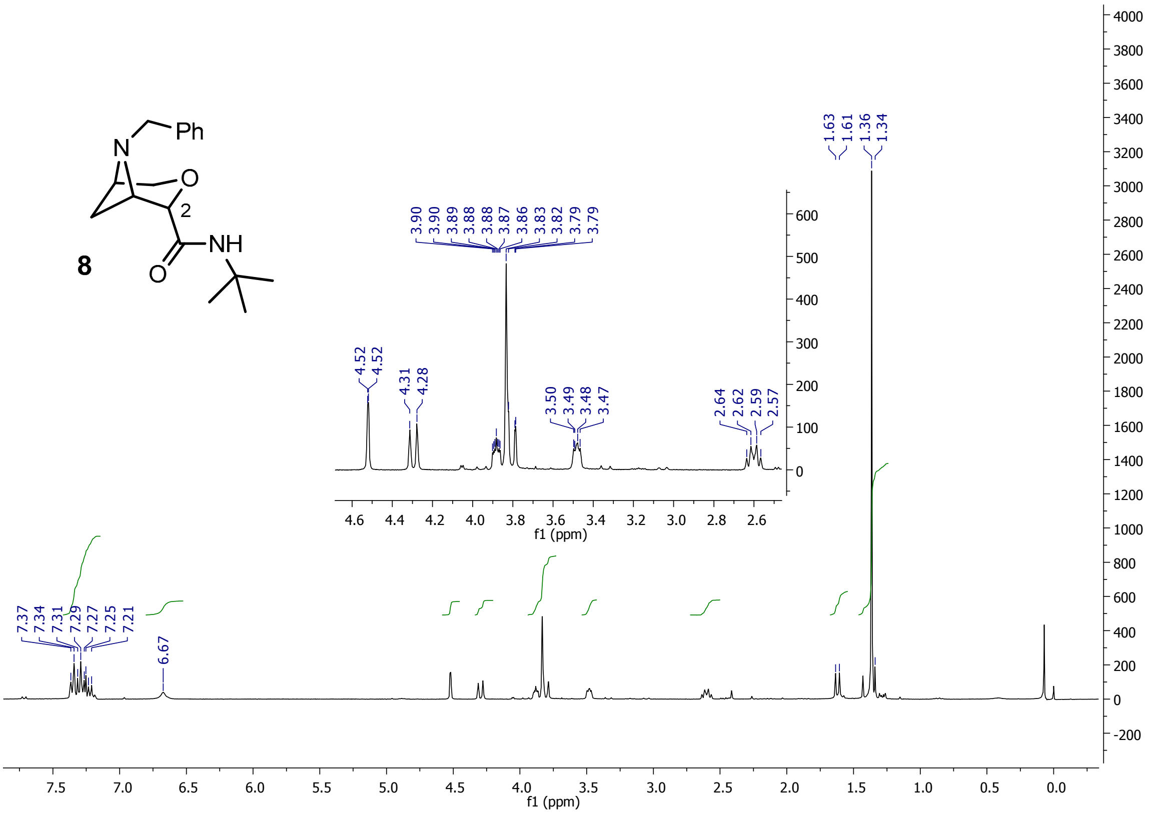
Ph
N
O
2
NH
O
8
3.90 3.90 3.89 3.88 3.88 3.87 3.86 3.83 3.82 3.79 3.79
4.52 4.52
4.31 4.28
3.50 3.49 3.48 3.47
2.64 2.62 2.59 2.57
1.63 1.61 1.36 1.34
7.37 7.34 7.31 7.29 7.27 7.25 7.21
6.67
f1 (ppm)

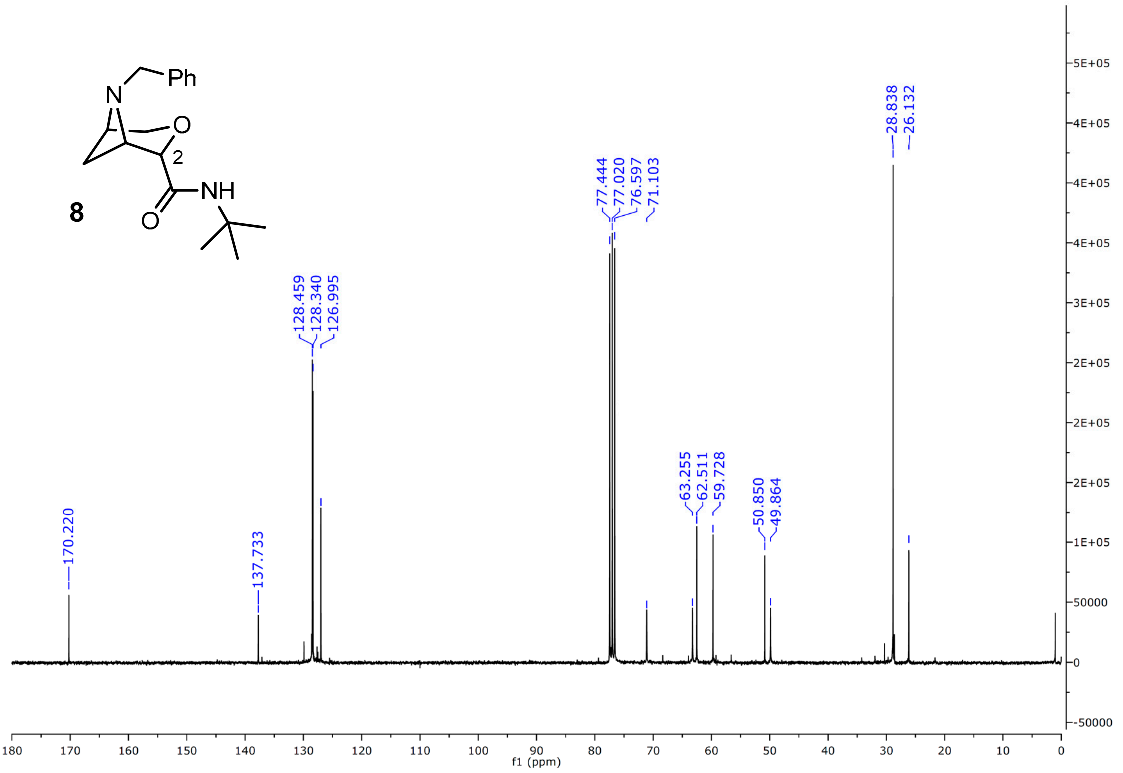
Ph
N
O
2
NH
O
8
170.220
137.733
128.459 128.340 126.995
77.444 77.020 76.597 71.103
63.255 62.511 59.728
50.850 49.864
28.838 26.132
f1 (ppm)
